# GoSynthetic database tool to analyse natural and engineered molecular processes

**DOI:** 10.1093/database/bat043

**Published:** 2013-06-27

**Authors:** Chunguang Liang, Beate Krüger, Thomas Dandekar

**Affiliations:** ^1^Department of Bioinformatics, Biocenter, Am Hubland, University of Würzburg, 97074 Würzburg, Germany and ^2^European Molecular Biology Laboratory, Meyerhofstr. 1, 69012 Heidelberg, Germany

## Abstract

An essential topic for synthetic biologists is to understand the structure and function of biological processes and involved proteins and plan experiments accordingly. Remarkable progress has been made in recent years towards this goal. However, efforts to collect and present all information on processes and functions are still cumbersome. The database tool GoSynthetic provides a new, simple and fast way to analyse biological processes applying a hierarchical database. Four different search modes are implemented. Furthermore, protein interaction data, cross-links to organism-specific databases (17 organisms including six model organisms and their interactions), COG/KOG, GO and IntAct are warehoused. The built in connection to technical and engineering terms enables a simple switching between biological concepts and concepts from engineering, electronics and synthetic biology. The current version of GoSynthetic covers more than one million processes, proteins, COGs and GOs. It is illustrated by various application examples probing process differences and designing modifications.

**Database URL:** http://gosyn.bioapps.biozentrum.uni-wuerzburg.de.

## Introduction

Proteins can form a variety of connections with each other forming different kinds of pathways. Synthetic biology aims to understand those pathways and to manipulate the original function to create new functionalities (1). Information on these is spread over the Internet and literature. Likewise, the actual annotations and interaction records themselves are scattered over a number of public resources (2,3).

Consequently, considerable information is needed for describing pathways and characterizing the function and structure of the pathways. This involves information stored in dedicated databases such as AmiGO (GO) (4,5), MIT BioBricks (6), COG and KOG (7,8), IntAct (9) and different organism-specific databases. Furthermore, algorithms (10–12) and tools such as STRING (13) have been devised that allow various interaction predictions.

Given all these distinct types and sources of pathway association information, it is highly desirable for users to have an integration that can be easily searched and browsed, at one single site.

The heart of GOSynthetic is to study molecular processes and their interactions: Molecular processes provide a cellular as well as molecular function, and GOSynthetic wants to understand, design and modify molecular processes. A broad gene ontology vocabulary analyses processes, but it is newly classified both from a molecular as well as an engineering and design perspective. The user can easily switch between both perspectives (‘molecular’ and ‘technical view’), and a simple top level classification is further refined regarding process timing, structure and interaction on a more detailed second level after which the detailed ontology classification follows. We specifically validated that the gene ontology vocabulary of GOSynthetic and its hierarchical classification is broad enough to specifically classify the COG/KOG families (7,8) (as a golden standard classification of well conserved ortholog molecular functions) and MIT BioBrick functionalities (6) (as a golden standard for synthetic biology design) for improved process analysis and design by GOSynthetic. A classification independent from AmiGO (4,5) is thus derived for GOSynthetic that nevertheless performs well on the other golden standards defining molecular functions. The same philosophy applies to process interaction predictions: Shared ontology terms suggest such interactions in GOSynthetic but we incorporated IntAct (9) as well as organism-specific databases such as TAIR, Wormbase, Flybase, EcoCyc, MGD, SGD, ZFIN and so on. To form an independent opinion of the quality of the information regarding a specific process interaction, the user can cross check with gene ontology, COG, MIT BioBricks for the logical consistency, and again independently, via the integrated Uniprot links.

In addition, our software provides an opportunity for interdisciplinary learning from both natural biology and biological engineering perspectives (for scientists of diverse backgrounds).

The GoSynthetic software resource integrates biological and synthetic biology knowledge.

## Results

The main strengths of GoSynthetic to analyse proteins and molecular interaction networks (natural, designed) in general are its unique comprehensiveness, standardization and interdisciplinary transfer possibilities. Standardization and clustering aspects build the basis to identify and define involved molecular processes for any protein or protein-interaction network of interest, predict their synthetic biology capabilities and finally enable easy and time saving queries. Standardization and interdisciplinary usage is achieved comparing a two-level module hierarchy for the classification of structural and functional aspects of natural biological versus engineered processes. Modules describe the process type (e.g., ‘regulation’ if a process is involved in regulation). The modules within the hierarchy cluster functional and structural information to achieve an easier comparison. GoSynthetic now covers 17 organisms ranging from virus to bacteria, fungi, plants and mammals: *Vaccinia Virus*, *Staphylococcus aureus*, *Listeria monocytogenes*, *Escherichia coli*, *Pseudomonas aeruginosa*, *Saccharomyces cerevisiae*, *Schizosaccharomyce pombe*, *Candida albicans*, *Arabidopsis thaliana*, *Caenrohabditis elegans*, *Drosophila melanogaster*, *Danio rerio*, *Gallus gallus*, *Bos taurus*, *Rattus norvegicus*, *Mus musculus and Homo sapiens.* One additional option is named *All*. It contains the GO-terms classification according to all organisms. Instead of proteins it contains the Uniprot Keyword classification which clusters proteins.

Seven modules (Tables 1 and 2) cover the basic definition of life-stressing emergent and system properties: The starting point of the classification uses typical defining terms for life. The seven modules cover the basic definition of life-stressing emergent and system properties (Table 1): Various definitions of life all converge and agree (details in Supplementary File 2) that living beings undergo metabolism (pathway modules), maintain homeostasis (regulation module), possess a capacity to grow (transformation module), respond to stimuli (sensing module), reproduce (system state module) and, through natural selection (operating on different cellular complexes), adapt (as complete entities / container module) to their environment in successive generations. From a design and engineering perspective, the emergent features of each classification module are the key aspect as well as their inherent options for control and regulation. Our new classification considers also GO terms, COG/KOGs and MIT BioBrick functions but stresses key functions and modules an individual protein may participate in. COG/KOGs provide the repertoire of objectively highly conserved molecular functions in prokaryotes and eukaryotes (7,8) whereas MIT BioBricks provide a strong repertoire to design novel molecular functions according to synthetic biology (6). In particular, we tested whether the gene ontology vocabulary of GOSynthetic and its hierarchical classification is broad enough to specifically classify all COG/KOG families and MIT BioBrick functionalities. This has the advantage that an independent classification is derived for GOSynthetic that nevertheless does not miss important COG or KOG terms (see Supplementary File 2 for the detailed coverage of COG/KOG terms achieved). Similarly, coverage of MIT BioBrick functionalities was achieved. In general a protein has several functions (in gene ontology for instance molecular function, cellular compartment and cellular processes it participates in). Also in our new classification of seven basic function types for living processes (‘modules’) in general a protein participates in multiple modules (or dimensions of emergence typical for the processes it is involved in). Well established technical terms fit into this classification again. The sub-modules of our classification (Table 2) are parts of their parental modules and stress different types of system changes that can occur within one module while after the sub-modules as second level a detailed further ontology classification follows. By this, we made sure that a rich vocabulary of process types and functions according to molecular biology and synthetic biology are available for the user to analyse molecular processes, and it is not necessary for the user to familiarize with the specific vocabulary of each of these different classification schemes (he can do so if he wishes of course, see tutorial and Supplementary Material), but corresponding classification is already directly provided by GOSynthetic and build in auto-completion and hints help the user when in doubt which term to add or to pick for analysis.

**Table 1.** Modules qualify the function, structure and state of a pathway, interaction and sub-network

Biological modules	Explanation
Sensing	Biological entities can be influenced via environment. They need sensors to notice changes in the environment and cause an effect.
Subunits	Transmitter, Receiver, Communication
Regulation	Biological entities have to be controlled and regulated. The regulation is used to maintain special biological settings or to enhance or alternatively silence biological effects.
Subunits	Activation, Inhibition
Pathway	Biological processes are organized within pathways. A pathway is a set of reactions to manage, command, or regulate the behaviour of systems directly or indirectly.
Subunits	Linear, Branching, Cycle, Cascading, Network
Transformation	Energy is converted in different forms of energy. Consequently transformation can deal with energy production or consumption or recycling of biological products.
Subunits	Anabolism, Catabolism, Metabolism, Degradation, Synthesis
Container	Containers are used to contain, store and transport substances or information.
Subunits	Information Storage, Transport Container, Transport Route
System state	Biological entities adapt to different states. In their life cycle they live, multiply, metabolize and die.
Subunits	Active, Dying, Inactive, Out of Control
Complex	Biological entities consist of different units or sub-modules. The complete functionality can only be achieved when all sub-modules are linked to a complex.
Subunits	Adjuvant, Barrier, Subunit, Whole Complex

**Table 2.** Mapping of biological and engineering modules

Module	Engineering term	Circuit example	Control engineering
Complex	Assembly		
Adjuvant	Cooperator		
Barrier	Obstacle	Resistor	
Subunit	Component		
Whole_complex	Entity		
Container	Box		
Information_storage	Data_medium		
Transport_container	Carrier	Capacitor	
Transportroute	Mobility	Wire	
Pathway	Process_structure	Circuit Factory/conveyor belt	
Branching	Splitting	Parallel circuit	
Cascade	Cascade		
Cycle	Circulation	Circuit	
Linear	Conveyor	Series circuit	
Network	Meshwork		
Regulation	Control_system	Diode	Controller
Activation	Stimulation	Positive resistor	
Inhibition	Reduction	Negative resistor	
Sensing	Detection	Inductor	Control variable
Communication	Reporter		
Receiver	Controller		Control element
Transmitter	Sensor		
System_state	Process_phase		State space controls
Active	On		
Dying	Off		
Inactive	Standby		
Out Of Control	Broken		Perturbation
Catabolism	Energy production	Electricity generation	
Degradation	Disassembling		
Metabolism	Remodelling	Transistor	
Synthesis	Production		

More than one million proteins are detailed classified by GoSynthetic into 32 module types. Starting from gene ontology classification, we tried to get a more complete classification of processes by considering also classification according to clusters of orthologous groups (Supplementary file 2 on the GoSynthetic website shows their classification and mapping according to GoSynthetic) and MIT BioBricks.

As a result, all the terms used in these classifications are searchable by the GoSynthetic user-interface: To analyse processes, the user picks single nouns describing the process and combines them with logical operators. In addition, adjectives for processes are searchable (the high number of gene ontology terms incorporated includes many process-classifying adjectives, e.g. inflammatory process or sensory process). Similarly, GoSynthetic distinguishes pathways (full well known pathways such as glycolysis, TCA and pentose biosynthetic process / pentose phosphate pathway) from transformations (no full pathways). In transformations it distinguishes whether they are synthesis (anabolic) or catabolism or just metabolic conversions, ‘metabolism’. For each of these two-level module types, the user can click after a query to see how many keywords or processes applying to the query are found to describe the process and its interactions in detail (see below). GoSynthetic alerts the user if a term or term combination did not yield results (as not contained in the hierarchy of terms describing processes) so that the user can adapt the search (e.g search the term ‘process’ with an adjective as many processes are described in this way in GoSynthetic, e.g. ‘inflammatory process’).

Descriptions for all module types that classify processes such as sensing or pathway can be found in Table 1, and the mapping to technical and engineering terms is presented in Table 2; further details are given in Supplementary File 2 on our website (see module establishment).

To investigate further organisms, a sequence-similarity database maps proteins to the best matching protein and its functional interaction partners and modules, using prokaryotic (*E. coli*, *S. aureus*) and eukaryotic (*H. sapiens*, *B. taurus*) model organisms. Cross-links to other databases are included; these allow an easy switch to further public protein databases, such as Uniprot or the Craig Venter Institute, the Arabidopsis Information Resource (TAIR) (14), WormBase (15), Candida Genome Database (CGD) (16), Flybase (17), EcoCyc and EcoliWiki (formerly known as EcoliHub) (18,19), Mouse Genome Database (MGD) (20), PseudoCAP (21), Rat Genome Database (RGD) (22), Saccharomyces Genome Database (SGD) (23), Sanger GeneDB (24), Zebrafish Model Organism Database (ZFIN) (25). Additional databases such as GO, COG (7,8), IntAct (9), NCBI Mesh terms and MIT BioBricks are integrated as well as the connection to Cytoscape (26).

The current GoSynthetic software and database combines accession links and information from all these databases. In addition it introduces an own classification concept and offers specific searches and visualization possibilities not implemented in the others.

Moreover, the unique and compact user interface and search options enable fast and ad hoc use of the resource, with no need for set-up or installation. Users may scroll the two-level classification of modules (module tree) for different organisms and visit a GoSynthetic encyclopedia to have a first impression. Besides this, GoSynthetic also offers a user-friendly interface for rapid location of processes of interest, including their functions, structures and interactions.

The scrolling option allows smooth translation of a more artificial, technical description (engineering, electronics and synthetic biology) into natural biological processes and *vice versa*. As an example we here show the translation of a technical temperature control device into biological proteins: As a starting point for the translation from a technical to a biological process, all proteins allowing the temperature detection have to be found. With GoSynthetic this is easily done via the module tree by selecting a specific organism and scrolling the appropriate ‘detection’ module. To fully implement a temperature control device using natural proteins, a temperature regulating protein can be found via the modules ‘controller’ and ‘energy production’.

With the organism and module tree, the user is able to search for proteins or COGs in a specific organism and module with the aim of implementing a certain technological process for synthetic biology by expressing the protein for the temperature circuit chosen in the organism of choice (see Supplementary Material file 1 with tutorial Figure T1–3; the Supplementary Material file 1 gives a tutorial on GoSynthetic software and database and further detailed information is available on our website http://gosyn.bioapps.biozentrum.uni-wuerzburg.de).

### Search modes

For an easy use of software and database, three different search scenarios are applicable, starting with a natural or engineered protein, a function or an organism of interest. Searches are based on process terms, on functional modules, or on protein sequence similarity. The results (process and function analysis, modifications and interactions) are visualized by text-highlighting functions, which are supported by graphical output on statistics and interaction networks involving partner proteins. As search item each kind of term (word, word combination, sentence) can be used, but for precise results only single words in each row should be used. Furthermore, the words that can be used are limited by the contents of the hierarchical tree of process terms and descriptions.

These cover nouns of gene ontology classification, COGs and MIT-Biobricks as well as adjectives for detailed process descriptions, e.g., ‘inflammatory process’. Thus, a search with ‘Transient receptor potential cation channel subfamily V member 4’ will retrieve no results, whereas the short protein name TRPV4 is fine. GoSynthetic allows to search four keywords at the same time, they can be connected to each other with three logical operators (‘and’, ‘or’, ‘not’). These keywords are ranked from top to bottom: The first keyword has always to be filled out and is going to be searched as primary keyword. The other terms follow automatically as further constraints. Furthermore, the user is alerted in the result page by accurate statistics on the hits obtained what happens by connecting the single term searches by the logical operators and whether any of the search terms did not yield a hit.

#### Keyword search (examples: keywords ‘sensor’, ‘kinase’, ‘smell’)

**Figure 1.**
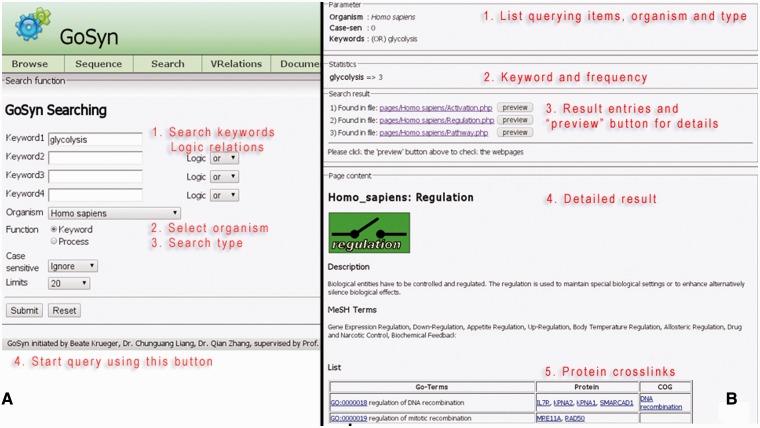
Keyword search. (A, left) *Query interface:* Using the keyword text field, one enters the terms of interest from primary to secondary with desired logical relationships (‘AND’, ‘OR’ and ‘NOT’) (see tutorial). After the searching organism type has been specified in the combo-box, the ‘Submit’ button is clicked next. (B, right) *Result page:* First (annotated by red lettering) the query parameters are listed. Then a brief statistic report summarizes different keywords and their frequencies present in the result, followed by the major process entries connected with the keywords. A button ‘Preview’ enables to visualize the corresponding key modules in more detail (listing involved subprocesses and proteins), the key module involved is shown, followed by the key module description. Then MEDline subject heading terms involved are given with functions and processes involving this cellular module. Finally (bottom) involved proteins are listed in a Table giving function (in GO-Terms), involved proteins as well as connected orthologous groups of proteins and genes.

The keyword search is provided complementary to an AmiGO search: The result is a list of all molecular modules that contain GO-terms that match the highlighted process. This kind of query type bases on text-mining, but it is very general and overall very useful to classify processes in GO-terms. It is possible to get a first impression about the process in question via the listed modules. GoSynthetic provides additional information on process regulation, on involved proteins and interactions, a function mapping according to COGs, and cross-links to many organism-specific databases (Figure 1, detailed usage see Supplementary Material file 1, Tutorial together with Figure T4). Furthermore, we implemented an auto-completion function that will list suggested keywords in a combo box when the user types a word in the text-field. With this feature, proper queries can be very efficiently given by users, e.g. to search ‘sensors of smell perception’, users could type ‘senso’, ‘perce’ and ‘sme’ in three rows, respectively, and GoSynthetic will generate hints automatically to complete them. The more important role this function plays is to avoid usage of inappropriate synonyms, e.g., noun instead of verb, choice of British English or American English term according to terms included in the database. Several different search examples have been prepared (use DEMO buttons), e.g. DEMO1 is a keyword search example ‘serotonin biosynthetic process of tryptophan’, results see application examples, DEMO2 searches ‘sensory perception of smell’ in GoSynthetic database and lists different receptors and signalling processes in the results page.

#### Term search

In addition, we offer an extension module for keyword searches; it will be invoked automatically if there are no strict matches in the result for a regular keyword search. The function can also be executed with the button (‘Term’) from the menu. This tool supports querying long sentences for accurate searches and even the sentence can be easily generated with the enhanced auto-hint feature for this module. However, we strongly suggest to query the primary term firstly. In this way of operation, GoSynthetic can dig out more information by truncating the suffix for the primary keyword, and step by step one can work through the complex sentence combining the different terms so find always the closest-related matches.

Keyword and Term search work for any term and hence organism, but are of course limited to the knowledge stored in the database (see methods: databases used). However, both process search and keyword search work particularly well for the 17 model organisms as for these data were directly incorporated.

#### Process search (example: glycolysis)

**Figure 2.**
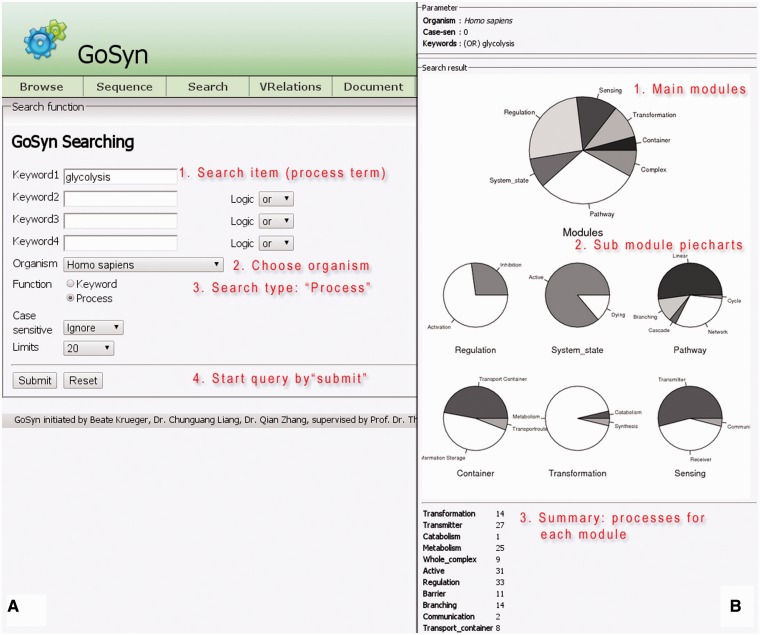
Process search. (A, left) *Query interface:* Any cellular process of interest can be investigated using this search function, once a proper process name is given (multiple process names are also accepted; they can be connected by specified logical relationships), an organism chosen and the query started by clicking the search button (annotation of instructions given in red). (B, right) *results page*: pie charts summarize different cellular modules and types involved in the process of choice and their relative importance in percent counting the number of further processes interacting with the queried process. For each module type, the relative amount of processes for its sub-categories is given (six pie charts in the middle of the page). A text listing (bottom) summarizes the exact occurrence of processes for each sub-category.

The process search is a specific query for a module (bundle of processes) yielding a more complex biological function. This can be a naturally occurring bundle of processes or an artificial engineered module. A process search is performed in the background; on the basis of the specified process it looks for all interactions and connected processes a process is involved in according to GoSynthetic. It first does a keyword search and looks up all ontology identifiers. Next GoSynthetic compiles a list of involved proteins and searches with these proteins in the specified organism for all relevant processes. So you get with the process search all directly interacting processes to a given process. As a result, all the module types covering the process or direct connected processes using this process and their various complex functions are presented in a result window provided as text information. For instance, DEMO4 button in the query page conducts a process search on ‘glycolysis’. Processes identified are listed first. The user obtains a list of processes sharing at least one ontology term with the process (or sharing an interaction, which also implies a shared ontology term) and only then the additional option to analyse the percentage and type of processes involved is invoked. In this second option (press button accordingly) a pie chart analyses the type of involved processes according to the two level classification of GOSynthetic. The types of modules are listed first and involve the module types ‘transformation’, ‘transmitter’ and ‘metabolism’ as top hits. Next the relative amount of processes involved is graphically illustrated in pie charts. The first pie chart gives an overview over all modules and implied functions (either from a biological point of view or, after toggling, according to engineering terms), while additional individual pie charts present the content of the sub-modules (for specific functional categories) within the parent module (Figure 2, usage: Supplementary Material file 1 with Tutorial Figure T5). A result comparison of process searches comparing the process glycolysis between different organisms is summarized in Table 3. Those searches do not only include direct connections to the glycolysis but also functions of the involved proteins which means it also finds indirect connections that cannot be found by text-mining. Consequently the entered search item can find more module types and processes than only the textmining entries. For instance, when we study glycolysis by process search, the module type ‘transmitter’ is covered by processes in *H. sapien*s 27 times but in *E. coli* only once. However, this process search reveals which other processes are connected with the process ‘glycolysis’. While in *E. coli* only sugar binding is identified many more processes and responses are annotated for *H. sapiens*: response to hypoxia, signal transducer activity, hormone activity, sugar binding, glucose binding, signal transduction, Ras protein signal transduction, sensory perception of sound, response to carbohydrate stimulus, response to glucose stimulus, response to zinc ion, response to muscle activity, second-messenger-mediated signalling, response to chemical stimulus, response to starvation, response to peptide hormone stimulus, regulation of transcription from RNA polymerase II promoter in response to oxidative stress, response to copper ion, phosphoinositide-mediated signalling, response to glucocorticoid stimulus. These transmitter-type processes found by GoSynthetic are clearly different from the glycolysis. There is no glycolysis process in the transmitter page of both *H. sapiens* and *E. coli*. Instead you find the process description for ‘glycolysis’ after search with the GoSynthetic keyword search for the organism *H. sapiens* in the module pages activation, regulation and pathway, whereas for *E. coli* it only appears in the pathway module page. Browsing retrieves glycolysis and anaerobic glycolysis in the pathway module type. Each process can be investigated further, for instance anaerobic glycolysis contains seven proteins (B4DJI1, B4DKQ2, LDHA, LDHB, LDHC, LDHAL6A, LDHAL6B). Furthermore, VRelation search (see below) shows for these interacting proteins, for instance LDHB has TBK1 as interaction partner. Here an additional option is named ‘All’. It contains the GO-term classification according to all organisms. Instead of organism-specific proteins it contains the complete Uniprot keyword classification. Process description categories are hence only left blank, if for all 17 model organisms there is no description in this category.

**Table 3.** Result comparison of process search with ‘glycolysis’ between different organisms and involved modules and submodules using GoSynthetic

Glycolysis	*H. sapiens*	*B. taurus*	*D. melangoaster*	*R. norvegicus*	*E. coli*	*S. aureus*	*P. aeruginosa*
Transmitter	27	4	7	44	1	1	0
Transformation	14	5	4	12	4	1	1
Catabolism	1	1	2	4	1	1	2
Metabolism	25	10	4	17	5	6	11
Whole_complex	9	6	4	13	0	0	1
Active	31	14	51	32	3	1	0
Regulation	33	8	13	31	0	0	0
Barrier	11	7	5	10	4	0	0
Branching	14	10	7	9	5	3	3
Communication	2	0	0	2	1	1	0
Transport_container	8	9	8	8	4	2	1
Network	36	15	45	36	5	5	6
Cascade	4	1	2	4	0	0	1
Synthesis	1	0	1	0	0	0	0
Complex	7	1	7	8	3	2	0
Adjuvant	2	1	1	1	0	0	0
Dying	5	2	4	5	0	0	0
Activation	51	20	8	49	1	1	0
Transportroute	1	1	1	1	1	0	0
Subunit	3	2	3	4	0	0	0
Pathway	5	3	3	5	4	4	2
Cycle	2	1	1	1	1	0	0
Receiver	21	9	8	15	0	0	0
Linear	61	36	22	61	26	22	7
Inhibition	19	2	21	19	0	0	0
Information_storage	8	3	8	8	2	1	0

#### Sequence search (Example: GTPase - HRas)

**Figure 3.**
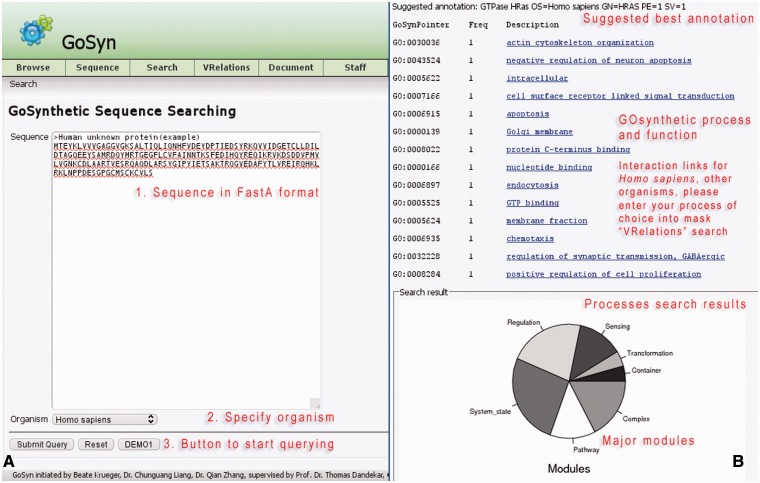
Sequence search. When the sequence of a protein is available, GoSynthetic can also be queried using its sequence searching module. *Entry page* (A, left): Any biological sequence of choice can be used including uncharacterized sequences of proteins with unknown function. The sequence is given in FASTA format entering the text field (see annotated sequence of instructions in red). By sequence comparison using BLAST, the GoSynthetic program performs a homology search on all sequences stored and identifies either the exact sequence as it is stored in the database or the best match closest to the entered sequence. (B, right) *Results page*: The very top gives the suggested annotation according to the identified best sequence match. Next all functions for the protein according to GoSynthetic are given, including GO-pointer (with GO identifier) and GoSynthetic process with link to a detailed listing of all involved proteins. Key modules identified encompass several involved processes. This is summarized in pie charts (further analysis of involved processes is possible e.g. by VRelation search).

This offers the possibility to investigate protein sequences directly (‘Sequence’ in the menu-bar). The user can query any sequence of interest (Figure 3, usage: Supplementary Material file 1 with Tutorial Figure T7). This query option uses a sequence similarity search engine which maps the protein sequence to six model organisms (*H. sapiens*, *R. norvegicus*, *D. melanogaster*, *S. cerevisiae*, *E. coli and S. aureus*). An organism-specific BLAST search is conducted with an E-value threshold of 0.00001. The top hit is automatically selected if a threshold of 75–125% coverage rate and 75% alignment identity is met in addition to the E-value cut-off. This sufficiently ensures that for most searches only orthologs are retrieved. However, in addition, the user can verify this by exploiting the cross-links warehoused in GOSynthetic if by gene onthology terms or according to COG/KOG classification the hit retrieved from the sequence search is a true ortholog or a paralog to the query sequence.

The resulting protein retrieved from the sequence comparison is next analysed regarding process classification and hierarchy (either in biological terms or in technical terms), and a list of processes obtained. Results may furthermore also be analysed for involved process types and their relative distribution in pie charts exactly like in the process search. Next, the user can perform further searches using either the GO-terms to yield functional classifications via keyword or process search (e.g. to design an experiment or to analyse the process structure further). Alternatively, the user can readily inspect the resulting protein–protein interactions by clicking the listed GO descriptions (in the example for the module ‘GO:0006935: chemotaxis’). A figure then reveals the resulting interaction relationships according to information from HPRD (*H. sapiens*). The demo button analyses the example protein sequence shown (GenBank accession CAG47067): On the results page GoSynthetic will give a suggested annotation with a GO term list. Furthermore, the functions, processes, and relevant interactions for the given sequence are calculated by GoSynthetic. For this, users can query (by clicking) the link on top (in the example shown the found function and module concerns ‘chemotaxis’). Next the results in Figure 3 (usage: Supplementary Material Tutorial Figure T6) will appear. They reveal the protein interactions around the query protein (marked by a blue arrow). Additional information concerns further pathways, processes, and interactions relevant to the identified module ‘chemotaxis’. Users have the option to export this interaction network into Cytoscape for further studies and other purposes. Besides analysing processes and functions pertaining to a given sequence, the sequence search can be used for a number of other applications, such as judging manipulated genomes by the classification of the additional or reduced protein interactions (see below), analysing differences between different strains using the classification of the varying proteins and interactions and to analyse artificial and engineered proteins (e.g. if a chimeric sequence is artificially constructed, both sequence parts will lead to different functional classifications).

#### VRelation search (Example: glycolysis and fatty acid)

**Figure 4.**
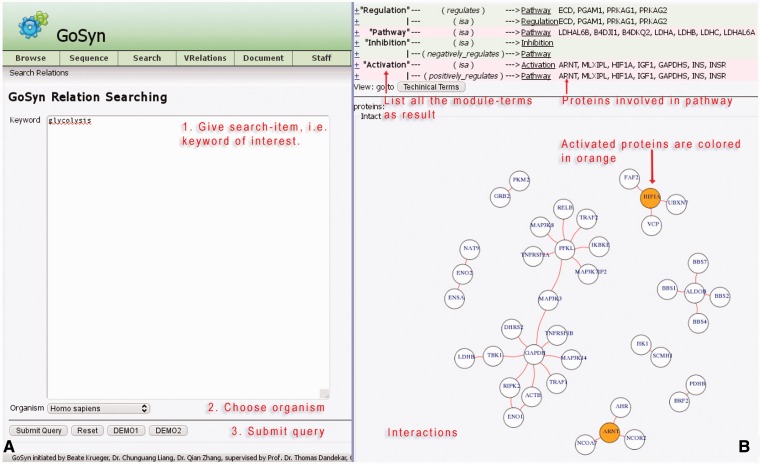
Relation search. (A) *Query interface (left)*: This allows (short instructions given in red) to investigate the interactions for a given keyword, e.g., ‘glycolysis’ (running example) for the organism ‘*Homo sapiens*’. Simple instructions are given in red. Two demonstration examples (DEMO1, DEMO2; bottom two buttons) give a rapid impression. (B) *Results page* (right): Annotation of the page is given in red lettering. A table (top) lists all the modules involved in the process ‘glycolysis’ according to the GoSynthetic database All the proteins known to be involved in the different modules, e.g., regulation, pathways, are listed on the right. A toggle switch changes from representation in technical engineering terms to natural molecular biology terms and back. The panel below shows all interacting proteins within the listed processes. For instance, aryl hydrocarbon receptor nuclear translocator (ARNT) and Hypoxia-inducible factor 1-alpha (HIF1A) proteins have an activating character (orange circles). The network visualization is plotted including data from the IntAct database.

As a further option for sequence search modes, a connection with the interaction database IntAct provides the opportunity to identify interactions and connections to other molecular biology processes including a graphical network visualization (Figure 4). In the current database, we prepared relationship information in detail for the six model organisms *H. sapiens*, *R. norvegicus*, *E. coli*, *S. aureus*, *D. melanogaster* and *S. cerevisiae*. Users can search the keyword of interest in this module directly, e.g. type ‘glycolysis’ in the keyword text-field, then carefully specify a model organism, e.g., *H. sapiens.* This is exactly the application example which can be loaded with the ‘**DEMO1**’ button (for more explanations see Supplementary Material file 1 on our website). As a result, a list is provided containing all modules and involved organism-specific proteins that have been identified using this process. The result list additionally illustrates all the related processes and modules as well as their connections, starting with biological terms. Users can easily switch between biological terms and technical terms using the button above the relationship view. For sequences from any organism, the closest related model organism from the above list should be chosen so that GoSynthetic can start to build the network based on the closest related model organism. For a distant organism, relation search will not show all relations correctly and present them only according to the model organism chosen by the user.

All the proteins involved in the process can be readily imported from GoSynthetic into other popular software such as Cytoscape for further investigations or to plan targeted modifications for synthetic biology and protein design. A second example illustrates the relationship view for processes connected to ‘fatty acid’ metabolism (DEMO2 button).

A process search generates the list of proteins involved in a process and its direct neighbour processes (technical or biological) of interest, visible in the upper part of the results area. Followed by a network figure, the relation search shows all interactions between the proteins found according to the IntAct database. Activating proteins are highlighted in orange, while inhibitory proteins are highlighted in blue. Protein–protein interactions identified according to the database are shown as red lines. The header of the relation search view lists in addition in which different modules the proteins shown are involved. Several modules may be involved depending on the proteins retrieved by the process search. Furthermore, this allows also to examine in more detail a certain pathway (an encyclopedia-like module enabled this additional option for GoSynthetic)

In Figure 4, the protein glyceraldehyde-3-phosphate dehydrogenase (GAPDH) appears as a central protein in lower glycolysis (using the option relation search). Besides its linear metabolic function in the pathway, there are a couple of regulatory interactions specific to higher animals, e.g. with mitogen-activated protein kinase 14 (MAPK14) and tumour necrosis factor receptor (TNFR54B).

GoSynthetic analyses cell processes in detail, integrating information from different genes and protein databases as well as several protein–protein interaction databanks. This allows to better investigate cellular processes including their modification in protein design and synthetic biology. Thus non-trivial additional information to the glycolysis in humans includes regulatory interactions such as aryl hydrocarbon receptor nuclear translocator (ARNT) and hypoxia-inducible factor 1-alpha (HIF1A), which both have activating character. Genetic interactions such as aldolase B (ALDOB) with the Bardet-Biedl syndrome proteins 1, 2, 4, 7 (BBS1, BBS2, BBS4, BBS7) and thus a connection between the glycolysis and the ciliopathic human genetic disorder of the Bardet-Biedl syndrome are easily detected via the interaction view relation search (VRelation) of GoSynthetic (steps: see tutorial in Supplementary Material).

A key advantage of GoSynthetic is the ability to immediately identify the involved structure of the pathway (process structure), for instance whether it is linear, branching or circular. This is directly shown by the process classification on top (in the example: ‘control system’ as well as stimulatory nodes are specifically listed). Further analysis for process structure is possible by looking at individual trees of the interaction network. The process comparison with other organisms is easily possible by just choosing another organism for the same process and search settings. The process structure is shown either in technical / engineering terms or in biological terms.

The visualized result and protein assembly can be readily exported into the open-source Cytoscape platform for the dynamic visualization of molecular interaction networks (26). This furthermore allows to modify the network and to assign additional data (gene expression metrics; any other comprehensive data) and/or detailed functionalities to each network element.

In summary, realization of processes and molecular modules in biological organisms and protein interaction networks are analysed by the three search modes including their function. This allows easy construction of user-defined new networks containing desired artificial or biological modules. The GoSynthetic portal can be used as a creative tool for process engineering or for the design of new experiments.

## Application examples

We illustrate the usage of the GoSynthetic software in the following examples, comparing artificial, engineered processes versus natural biological processes according to current knowledge.

(i) *Comparative analysis of process design in different organisms*

**Figure 5.**
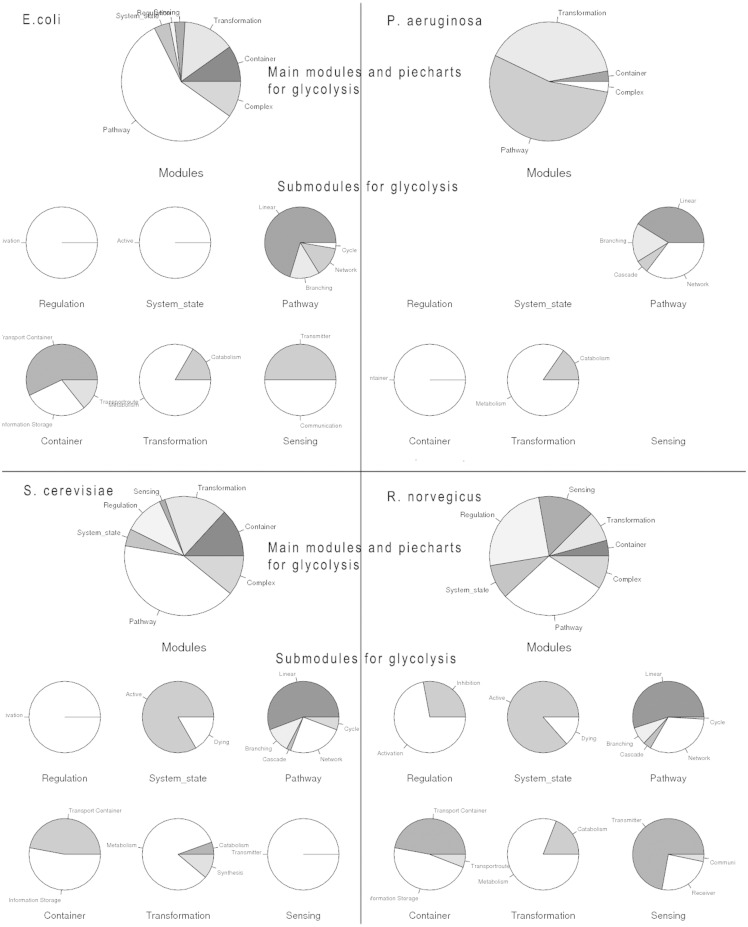
Analysis of the process ‘glycolysis’. Glycolysis is investigated and compared for different organisms (*E. coli, P. aeruginosa*, *S. cerevisiae, R. norvegicus*) with the GoSynthetic process search. For each organism a process search for the term ‘glycolysis’ was carried out; afterwards a pie chart regarding the types of modules involved in the process was generated. The pie chart for the main modules is firstly displayed, followed by the pie charts for the sub-modules. If a detailed list of regulation in glycolysis (for example) is desired, users can next perform a keyword search by typing ‘glycolysis’ in the text field (see Figure 1). All the proteins involved will be sorted according to their GO-terms, and then displayed in a result table.

Processes in different organisms can be compared readily and in a user-friendly manner via the process search. Figure 5 compares the glycolysis and directly interacting processes in *E. coli, P. aeruginosa, S. cerevisiae* and *R. norvegicus.* Differences in processes interacting with glycolysis become readily apparent (a larger comparison includes *B. taurus, D. melanogaster and S. aureus*, see Supplementary Material Figure T8a–g). Processes in *P. aeruginosa* occur especially in transformation modules; yeast and rat have a high involvement in regulation modules. In general, higher developed organisms seem to focus more on activation and transmitter modules while lower organisms centre on metabolism (Table 3). This is apparent for eukaryotes compared with prokaryotes; specific differences in individual organisms are also visible and can be further analysed. Statistical analysis is possible applying R; for rapid and easy analysis, we offer an easy-to-use *t*-test module (data of two samples with a sufficient number of observations are entered in the text-field, the significance value will be calculated, in this case a sufficient number of eukaryotic against prokaryotic organisms can be compared for each module-type and t-values collected). The function can be performed with the ‘analysis’ button in the menu. Two datasets are required. For instance, these are observed frequency lists comparing process-search results between different organisms such as glycolysis process search comparing *H. sapiens* and *B. Taurus* (the DEMO button runs such an example for the user). Results appear in a separate page and include *p*-value, *t*-value as well as percent confidence interval and mean values.

(ii) *Engineering a metabolic process*

In general, for engineering there are several options in GoSynthetic: (i) the choice between the technical/engineering view and the molecular biology view. (ii) Furthermore, it is possible to compare lists and process structure in different organisms using different comparison windows and then to derive from this process comparisons suggestions for engineering. This includes direct statistics and term counts as well as *t*-test comparisons between different process queries. (iii) Furthermore, cross-links to other databases allow readily further comparisons regarding organism-specific proteins, protein families etc.

As an example for engineering a metabolic process, we use the tryptophan synthesis (entered search term is ‘tryptophan’) and compare this in the organisms *E. coli* and *H. sapiens* applying keyword as well as process searches. Combining always and only single terms, in this example ‘tryptophan’ with ‘synthesis’, results are as follows: ‘tryptophan’ 7 terms, ‘synthesis’ 8 terms, dissection with the logical operator ‘AND’ yields 1 shared term, whereas union with ‘OR’ operator yields 14 terms.

For process analysis of tryptophan synthesis in *E. coli*, we hence refer to the ‘tryptophan biosynthetic process’ (the entered search keywords are ‘tryptophan’ and ‘biosynthetic’; the logical relationship is ‘AND’). The key word search shows two module types the process is involved in (text mining reveals all processes where either tryptophan or trp genes or tryptophan gene ontology terms play a role). The second module type (‘Network’) includes metabolic networks and includes thus as a result GO: 0000162, containing the tryptophan synthase proteins TrpB, TrpC, TrpD, TrpE, TrpL. This prokaryote has the capability to synthesise tryptophan on its own (Supplementary Material Figure S1).

In *H. sapiens*, tryptophan is an essential amino acid, and there is no tryptophan synthesis present. However, tryptophan can also be used for secondary metabolism in humans, such as serotonin production. For instance, there is the process ‘serotonin biosynthetic process of tryptophan’ (this is readily found by keyword search combining the keywords ‘serotonin’, ‘tryptophan’, ‘biosynthetic’, logical relationships are two ‘AND’ operators). Users can rely on the auto-hint system to have the three keywords completed (for illustration click ‘DEMO1’ button). For more information, the user clicks on it (go to module type ‘network’, there to ‘serotonin biosynthetic process from tryptophan’ and then you see that this process involves the human tryptophan hydroxylase proteins TPH1 and TPH2). Similar secondary processes such as melanin or dopamine synthesis are shown by GoSynthetic and can also be investigated for modifications. Biotechnological production of secondary metabolites in *E. coli* is sped-up by such comparisons; required enzymes and interactors can be easily chosen and expressed; involved regulatory processes are also rapidly identified.

(iii) *Analysis of process structure around a central hub*

Central networks are redundantly constructed and involve a number of processes which does not make modification easy. We show this with the help of the process search (for instance) for inflammation in mouse. Besides pie charts compare in detail all the processes involved in modules and sub-modules; it is also possible to show a relation-view. This relation-view is generated by selecting the organism ‘mouse’ (as example, the user can of course choose the organism of choice) and entering ‘process’ and ‘inflammatory’ as keyword; the logical operator used is ‘AND’. As already mentioned in the introduction, GoSynthetic has characterized many processes, but uses for these adjectives (‘inflammation’ as key word will retrieve nothing).

**Figure 6.**
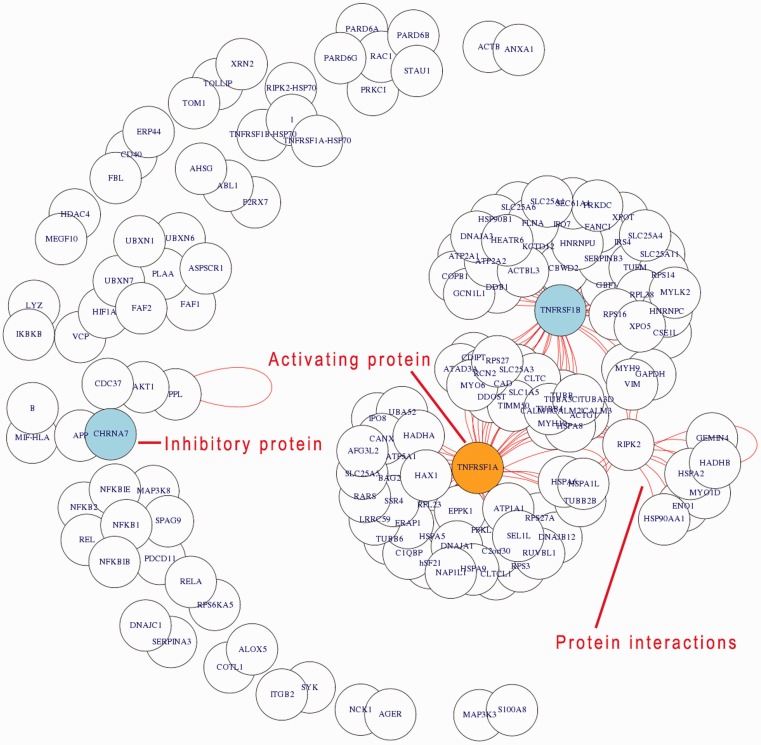
Relation-view. The application example shown concerns the analysis of inflammatory processes in humans. Querying used the keyword ‘inflammatory’ and ‘process’ for the selected organism ‘*Homo sapiens*’. The generated network visualization shows the interacting proteins within the inflammation process. Interactions are indicated by red lines (short lines covered in the crowded output, but clearly visible for the central hubs as indicated in the result figure). Activating proteins are coloured in orange, whereas inhibitory proteins in blue.

Figure 6 shows in a relation-view that the inflammatory process in humans is highly complex and contains a large amount of interacting proteins, which indicate a strongly regulated process. All the listed proteins are functionally or physically connected to the inflammatory process. Actual physical protein–protein interactions are shown by red lines. Tumour necrosis factor receptor protein 1 and 2 (gene names: TNFRSF1 and TNFRSF2) are central hubs within the inflammation process mediating activation and inhibition processes. Between these counterparts the receptor-interacting serine/threonine-protein kinase 2 (RIPK2) is placed functionally ‘connecting’ CASP8-mediated apoptosis and the activation of the transcription factor NF-kappa-B. This kind of result (relation-view, exact usage see tutorial in Supplementary Material) shows the possibility of recovering signalling pathways (like the NF-kappa-B) by GoSynthetic and its combination of functions and interactions. In fact, if a complete or partial pathway is directly associated with an interaction or process it will always turn up, if the connection is looser (two or more mediating functions); this is only the case if all components are member of a common interaction network or part of an overarching process. Hence, the example view shows hubs and interacting proteins including process and type of interaction. Design and engineering experiments can build on this information. GoSynthetic allows to compare now this view with the inflammatory process in other organism in a second window. Statistics are given regarding the number of connected processes, proteins and modules. Furthermore, a *t*-test window allows to identify statistical significant differences.

This relation-view is automatically generated from the database for the inflammatory process. The database allows rapid switching and comparing different organisms, and again specific processes and sub-processes can be analysed. For more detailed analysis the relation-view can be exported to Cytoscape. These options are helpful for more carefully planning protein engineering experiments or other designed modifications around central hub proteins.

(iv) *GoSynthetic: Artificial design in protein engineering*

Introduction of artificial new circuits with new properties is a central effort in synthetic biology. For instance, two-component systems from *E. coli* were engineered applying artificial modified *E. coli* strains, which are able to sense light and establish growth patterns accordingly (27).

GoSynthetic allows further planning of such artificial design experiments including search for drug targets, novel pathways as well as modify regulatory input critical for certain processes. GOSynthetic compares processes from various organisms. We illustrate this for *A. thaliana* and *H. sapiens.* Supplementary Table S2 shows the table-view. This lists the module and protein distribution generated with the process search. In this example, please use logical operator (‘AND’) to combine again two single terms: ‘reaction’ as first keyword and ‘light’ as second keyword. In total, GoSynthetic currently identifies different processes regarding reaction to light for 17 organisms.

From the plant data, artificial design on processes involved in reaction to light could focus on catabolism modules (e.g. change downstream the amount of starch production). Modifying other network modules is an alternative, for instance scrutinizing the process around light harvesting to improve energy generation in engineered plants. In *H. sapiens* processes regarding light occur only in the module type ‘sensing’ (sensors for the stimulus light) or in the type ‘receiver’ as a subsequent reaction to light. Modification of transmitter modules in *H. sapiens* would concern completely different processes and possibilities (e.g. by new drugs, modification of circadian rhythm). GoSynthetic shows that human processes involve further such target points in addition to the well known rhodopsin (e.g. excitatory amino acid transporter, COP9 signalosome complex, dopamine receptor, fech-protein, gap-junction proteins, methionine adenosyltransferase and Synembryn-A; see Supplementary Material Figure S2).

(v) *GoSynthetic: Design of a synthetic organism*

Large-scale designs in synthetic biology can easily be planned with the GoSynthetic software. For example streamlining an organism in biotechnology for optimal yield, engineering of oncolytic viruses or even nuclear transfer experiments can be designed, carefully considering involved processes and their structure.

The next example analyses experiments concerning growth hormone. A first experiment would modify the organism (in body size and maybe overall) by overexpression of growth hormone. This has been done, for instance in mice (28). The phenotype created by over-expression of growth hormone in transgenic mice is surprisingly complex, as the growth hormone influences a number of pathways and networks. GoSynthetic is easily capable to handle this. We show the classification of affected processes according to GoSynthetic. Table 4 is edited from the full results obtained. It summarizes interesting modules identified (doing a keyword search combining the two terms ‘growth’ and ‘hormone’ with the logical operator ‘AND’). The actual list on the GoSynthetic webpages resulting from this query has more details and is five pages long (displayed on the GoSynthetic webpages after querying GoSynthetic as just described). Table 4 right column indicates from which page and module type the highlighted entry was taken. The transgene phenotype has changes regarding the reaction to cocaine, drug, food, light stimulus, morphine and smell. Additionally, it is involved in the activation of appetite, cell proliferation, insulin-regulated processes, inhibition of blood pressure and cell migration. The whole complex list was generated from querying GoSynthetic with ‘growth’ and ‘hormone’, logical operator ‘AND’, setting mouse as organism, in a process search over the database. Further information on each process is available using the above query options looking at each connected process and keyword by new searches and analyses in GoSynthetic. As is seen in natural adaptation of animals in changes to body size, further biomedical research effort has to consider balancing these complex changes seen from the analysis by GoSynthetic. This may imply a new experiment with further modified transgenic mice. However, the GoSynthetic process and interaction analysis is also helpful in examining new therapeutic options (or molecular targets) in growth hormone disorders. GoSynthetic delivers by process searches always results of direct process interactions; however, it considers the full hierarchy of process description terms and hence provides rich information about connected processes. In this example, ‘sensory perception of smell’ is a neighbour process directly influenced by tempering with growth hormone in the transgenic animal. With the user interface and the single keyword combination by logical operators, it is also clear what to do if one now wants to investigate this neighbour process: Firstly, users could type ‘sensory’, ‘perception’ and ‘smell’ as keywords, logical operators could be either ‘AND’ (intersect, recommended) or ‘OR’ (union) for different searching purposes as criteria.

**Table 4.** Listing of all processes and modules involved with the growth hormone in mouse

GO_name	Module
activation of adenylate cyclase activity by G-protein signaling pathway	Activation
activation of MAPK activity	Activation
activation of phospholipase C activity by dopamine receptor signaling pathway	Activation
Appetite	Activation
cAMP biosynthetic process	Activation
neuroblast proliferation	Activation
neuron differentiation	Activation
peptidyl-tyrosine phosphorylation	Activation
Translation	Activation
organ growth	Activation/Active/Regulation
insulin secretion	Activation/Branching/Inhibition
insulin-like growth factor receptor signaling pathway	Activation/Communication
cell proliferation	Activation/Inhibition
hormone secretion	Activation/Inhibition
multicellular organism growth	Activation/Inhibition/Regulation
growth hormone secretion	Activation/Inhibition/Transmitter
signal transduction	Activation/Inhibition/Transmitter
dopamine uptake	Activation/Regulation
Synaptogenesis	Activation/Regulation
cell migration	Inhibition
cortisol secretion	Inhibition
inhibition of adenylate cyclase activity by dopamine receptor signaling pathway	Inhibition
prepulse inhibition	Inhibition
protein kinase B signaling cascade	Inhibition
dopamine receptor signaling pathway	Inhibition/Receiver
blood pressure	Inhibition/Regulation
behavioral response to cocaine	Transmitter
behavioral response to ethanol	Transmitter
cellular response to insulin stimulus	Transmitter
hormone metabolic process	Transmitter
hormone-mediated signaling	Transmitter
intracellular signaling cascade	Transmitter
response to amphetamine	Transmitter
response to cocaine	Transmitter
response to drug	Transmitter
response to food	Transmitter
response to light stimulus	Transmitter
response to morphine	Transmitter
sensory perception of smell	Transmitter

The user is also guided by the availability (frequency in the result page) of each term. In fact, the different terms describing a process have been optimized to cover as much as possible according to gene ontology, different COG classes (Supplementary file 2) and the MIT BioBricks. Classification is, however, accordingly to these major classification schemes; hence, for example, GO:0007231, osmosensory signalling pathway, is categorized as sensing module type. For this example case a keyword search allows rapid location of the process, using single keywords: ‘osmosensory’, ‘signaling’, ‘pathway’, connected with the logical operator ‘AND’. GoSynthetic reports then the following frequencies: pathway: 19, osmosensory: 2, signalling: 14. Involved processes cover two module types in *H. sapiens*, these are ‘inhibition’ and ‘sensing’. For retrieving the same by browsing Go-Synthetic, one has to search in the correct module. Here, one has to browse the sensing module, and the first process identified is ‘osmosensory signaling pathway’ (GO:0007231).

## Design and Implementation

This is an enhanced database resource to study and design molecular processes including a number of navigation options between database pages as well as user-friendly software features such as automatically generated hints for a higher user-friendliness. This reduces typing mistakes (including term dialects) and improves the efficiency (auto-completion retrieves successful and rapidly information). Furthermore, the ‘term’ search module supports not only keywords but also phrases and short sentences. Furthermore, we included an extended list of Gene Ontology terms. The database is regularly updated. Protein identifiers from organism-specific databases (latest versions) are merged into each updated GoSynthetic database.

### Implementation

GoSynthetic contains different organisms and different data sources. Supplementary Table S1 lists the association counts. The data source for this analysis was originally adapted from the Gene Ontology Server of 2012; we kept it being continuously updated to be consistent with the latest release, including recently corrected GO numbers and descriptions (Feb. 2013). The IntAct database (version 2010) as well as the latest sequences and functional annotations from KOG (eukaryotic orthologous groups) in addition to COG (Clusters of Orthologous Groups of proteins), and the MIT standard biological parts (29) are all integrated and considered when querying the GoSynthetic database.

**Figure 7.**
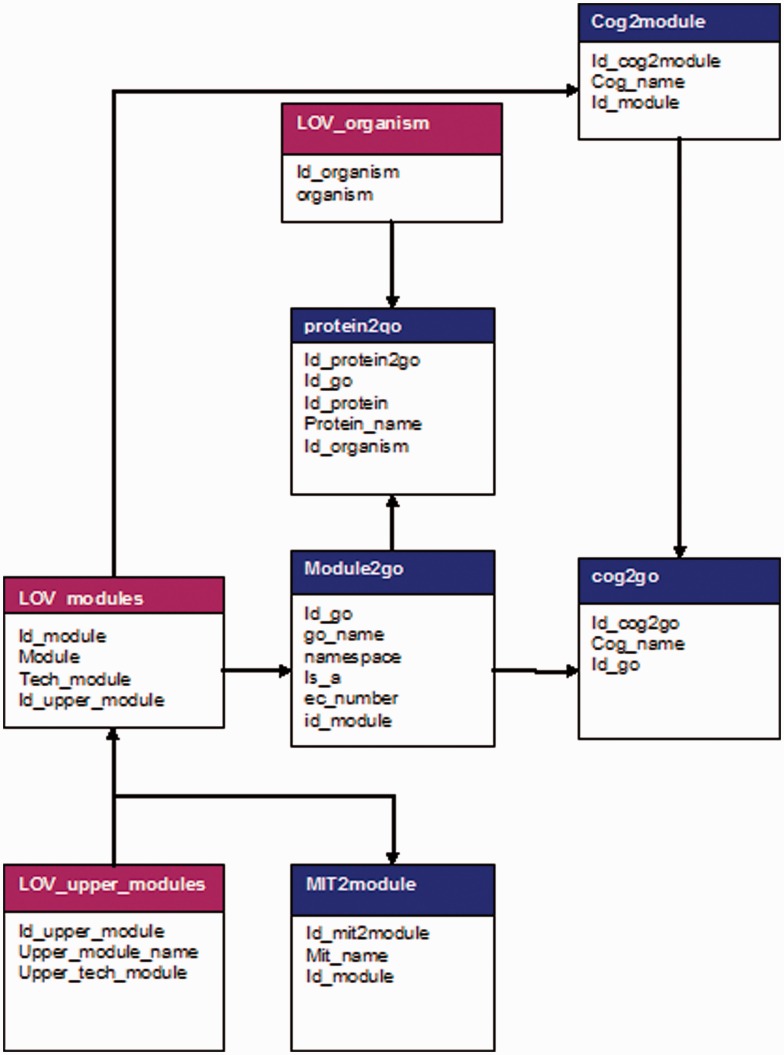
Entity–relationship diagram of GoSynthetic database. The Entity–relationship model of GoSynthetic is illustrated in the figure. Different types and sources of data covering COG/KOG, organism-specific proteins and interactions have been merged and organized into GoSynthetic database.

All the data were extracted and processed using Perl (version 5.16.0) scripts with help of BioPerl modules (version 1.6.901) (30); the collected processes, interactions and terms for both biological and technical are warehoused into a MySQL database (version 5.5). The design of the data repository has been described in the ER diagram (Figure 7); more details on data maintenance can be found in Supplementary Material. Data source integration is achieved comparing existing protein identifiers. The mapping to GoSynthetic processes and modules itself was achieved by text-mining scripts and manual validation, i.e., we changed the mapping until the respective coverage of the different databases both by the biological or technical classification or any of the database classifications was as high as possible (numbers and percentages see Supplementary Material).

Several visualization elements, e.g., tree-menu, are deposited in XML. Statistic figures were generated using Postscripts and then converted into images with GD libraries. A user-friendly interface was established; the code was implemented in PHP (PHP Hypertext Preprocessor) using AJAX (Asynchronous JavaScript and XML). JQuery libraries (version 1.9) are generally applied for efficient data retrieval operations, i.e., dynamic previews, combo boxes with autocomplete feature, treelist visits according to the respective organism, biomolecules derived from, and multiple tabbed-views. We warehoused the knowledge tree (pages and sub-modules) into XML files. Once users search these modules or even browse the encyclopedia from the tree-menu, pages will be loaded, selected and previewed in the content view. In addition, there is auto-completion of terms (hints for the user). All of these are implemented with AJAX. The layout and user options work for popular browsers tested (i.e., mozilla firefox and internet explorer for different OS respectively).

### GoSynthetic Design

In addition to browsing the module encyclopedia, a search module for rapid locating processes and modules of interest was also enabled. Regarding interacting processes or processes a certain protein is involved in process type and relative usage is summarized by different pie charts. The integrated relation search allows the user to inspect the relationships between related proteins when taking into account the engineered synthetic modules. Here we integrated different interaction databases such as the IntAct database. Users can easily switch functional interpretation between engineering terms and biological terms. Pathway analysis relies on a modified VRelation search on the pathway of interest. A resulting interaction network is computed and plotted using the statistic tool R with the iGraph library. The Fruchterman Reingold algorithm can be applied to obtain an optimal layout. In the illustration, orange circles indicate the activation processes, blue indicate inhibitory processes, whereas green circles are metabolism related. Other options for further exploration of the molecular data were also enabled, e.g., via Cytoscape, which is an open source bioinformatics software platform for visualizing molecular interaction networks. Homology mapping of protein interactions is performed on the basis of the interactome relationships in six organisms (human as well as *H. sapiens, B. taurus, R. norvegicus, D. melanogaster, E. coli* and *S. aureus*) The interaction profile can be readily downloaded below the figure and imported into Cytoscape workbench, enabling users to modify network relationships, and including layouts, adding or removing certain proteins from the system.

Querying any protein sequence of interest involves first a sequence-to-sequence comparison. This yields (within seconds) the most related protein sequence (either *E. coli, S. aureus, R. norvegicus, D. melanogaster,* or *H. sapiens, B. taurus*), determines the involved GO-terms, analyses the type of modules (biological classification first) involved and delivers a pie chart according to the process types implicated by this protein according to the knowledge from these model organisms. With this output, in particular the GO-terms in the description list, further analysis by the user is possible. In particular, an interaction chart can be illustrated by simply clicking the description (top of the page) with the GO-terms, or typing them in the GoSynthetic keyword and the relation search for more details.

## Availability and Future Directions

The present GoSynthetic version is publicly available at http://gosyn.bioapps.biozentrum.uni-wuerzburg.de; its integrated database includes 17 model organisms as well as more than one million proteins, including their cross-links to external databases. Six organism-specific protein interaction networks (*E. coli, S. aureus, R.norvegicus, D. melanogaster, H. sapiens, B. taurus)* have been data-mined and warehoused in the current database. Furthermore, it allows the analysis of any protein sequence of interest (including artificial engineered sequences) regarding function, interaction and processes, both in biological processes and for design in protein engineering and synthetic biology.

Our example cases demonstrate GoSynthetic at work. Approaches such as these are expected to gain further power, as the efforts in synthetic biology will get momentum. We will further extend the database with focus on organisms of interest for synthetic biology and biotechnology such as mollicutes, adenoviruses, different yeasts and further rodent genomes.

Major advantages of GoSynthetic as compared with currently available tools include: For process and function analysis in molecular biology, GoSynthetic provides more searching and visualization possibilities than others. By including GO-terms as well as COG information, MIT Biobricks, IntAct and cross-links to further databases it delivers more information on function, and the information is better connected than from many text-mining tools such as AmiGO (5). Competitive protein-interaction tools [e.g. STRING (13)] are extended in GoSynthetic by information about protein function and modules, sub-modules and functional processes, both in a natural and an artificial engineering classification. Comparisons with other organisms or engineered processes are easily possible, and new perspectives based on the various levels of comparison stimulate research efforts in synthetic biology or biotechnology.

## Supplementary Data

Supplementary data are available at *Database* Online.

Supplementary Data

## References

[1] Camacho DM, Collins JJ (2009). Systems biology strikes gold. Cell.

[2] Galperin MY, Cochrane GR (2011). The 2011 Nucleic Acids Research Database issue and the online Molecular Biology Database Collection. Nucleic Acids Res..

[3] Ulrich LE, Zhulin IB (2010). The MiST2 database: a comprehensive genomics resource on microbial signal transduction. Nucleic Acids Res..

[4] The Gene Ontology Consortium (2012). The Gene Ontology: enhancements for 2011. Nucleic Acids Res..

[5] Balsa-Canto E, Banga JR (2011). AMIGO, a toolbox for advanced model identification in systems biology using global optimization. Bioinformatics.

[6] Shetty RP, Endy D, Knight TF (2008). Engineering BioBrick vectors from BioBrick parts. J. Biol. Eng..

[7] Kaufmann M (2006). The role of the COG database in comparative and functional genomics. Curr. Bioinform..

[8] Tatusov RL, Fedorova ND, Jackson JD (2003). The COG database: an updated version includes eukaryotes. BMC Bioinformatics.

[9] Aranda B, Achuthan P, Alam-Faruque Y (2010). The IntAct molecular interaction database in 2010. Nucleic Acids Res..

[10] Yu Q, Li GH, Huang JF (2012). MOfinder: a novel algorithm for detecting overlapping modules from protein-protein interaction network. J. Biomed. Biotechnol..

[11] Qin G, Gao L (2012). An algorithm for network motif discovery in biological networks. Int. J. Data Min. Bioinform..

[12] Suravajhala P, Sundararajan VS (2012). A classification scoring schema to validate protein interactors. Bioinformation.

[13] Szklarczyk D, Franceschini A, Kuhn M (2011). The STRING database in 2011: functional interaction networks of proteins, globally integrated and scored. Nucleic Acids Res..

[14] Swarbreck D, Wilks C, Lamesch P (2008). The Arabidopsis Information Resource (TAIR): gene structure and function annotation. Nucleic Acids Res..

[15] Harris TW, Antoshechkin I, Bieri T (2010). WormBase: a comprehensive resource for nematode research. Nucleic Acids Res..

[16] Inglis DO, Arnaud MB, Binkley J (2012). The Candida genome database incorporates multiple Candida species: multispecies search and analysis tools with curated gene and protein information for *Candida albicans* and *Candida glabrata*. Nucleic Acids Res..

[17] McQuilton P, St Pierre SE, Thurmond J (2011). FlyBase 101 - the basics of navigating FlyBase. Nucleic Acids Res..

[18] Keseler IM, Collado-Vides J, Santos-Zavaleta A (2011). EcoCyc: a comprehensive database of *Escherichia coli* biology. Nucleic Acids Res..

[19] McIntosh BK, Renfro DP, Knapp GS (2011). EcoliWiki: a wiki-based community resource for *Escherichia coli*. Nucleic Acids Res..

[20] Eppig JT, Blake JA, Bult CJ (2012). The Mouse Genome Database (MGD): comprehensive resource for genetics and genomics of the laboratory mouse. Nucleic Acids Res..

[21] Winsor GL, Lo R, Ho Sui SJ (2005). *Pseudomonas aeruginosa* Genome Database and PseudoCAP: facilitating community-based, continually updated, genome annotation. Nucleic Acids Res..

[22] Shimoyama M, Smith JR, Hayman T (2011). RGD: a comparative genomics platform. Hum. Genomics.

[23] Cherry JM, Hong EL, Amundsen C (2012). *Saccharomyces* Genome Database: the genomics resource of budding yeast. Nucleic Acids Res..

[24] Logan-Klumpler FJ, De Silva N, Boehme U (2012). GeneDB-an annotation database for pathogens. Nucleic Acids Res..

[25] Bradford Y, Conlin T, Dunn N (2011). ZFIN: enhancements and updates to the Zebrafish Model Organism Database. Nucleic Acids Res..

[26] Cline MS, Smoot M, Cerami E (2007). Integration of biological networks and gene expression data using Cytoscape. Nat. Protoc..

[27] Levskaya A, Chevalier AA, Tabor JJ (2005). Synthetic biology: engineering *Escherichia coli* to see light. Nature.

[28] Hammer RE, Brinster RL, Rosenfeld MG (1985). Expression of human growth hormone-releasing factor in transgenic mice results in increased somatic growth. Nature.

[29] Galdzicki M, Rodriguez C, Chandran D (2011). Standard biological parts knowledgebase. PLoS One.

[30] Stajich JE, Block D, Boulez K (2002). The Bioperl toolkit: Perl modules for the life sciences. Genome Res..

