# Supplementary material for: GoSynthetic database tool to analyse natural and engineered molecular processes
Source: Database (Oxford). 2013 Jun 27;2013:bat043. doi: 10.1093/database/bat043 (PMC3694605; doi:10.1093/database/bat043)
Supplement: Supplementary Data [file supp_2013_bat043_index.html]

GoSynthetic database tool to analyse natural and engineered molecular processes — Supplementary Data 

# GoSynthetic database tool to analyse natural and engineered molecular processes

## 

files

**Files in this Data Supplement:**

- Supplementary Data - doc file
- Supplementary Data - doc file
