# Supplementary material for: GoSynthetic database tool to analyse natural and engineered molecular processes
Source: Database (Oxford). 2013 Jun 27;2013:bat043. doi: 10.1093/database/bat043 (PMC3694605; doi:10.1093/database/bat043)
Supplement: Supplementary Data [file supp_bat043_Database_GoSynthetic-supplmaterial-file1-f.doc]

### Database Tool

**GoSynthetic database tool to analyze natural and engineered molecular processes**

**(supplementary file 1)**

Chunguang Liang^1,§^, Beate Krüger^1,§^, Thomas Dandekar^1, 2,*^

^§^ both authors contributed equally to this work

^1^dept of bioinformatics, biocenter, Am Hubland, University of Würzburg, 97074 Würzburg, Germany

^4^European Molecular Biology Laboratory, Meyerhofstr. 1, 69012 Heidelberg, Germany

^*^corresponding author

**Contents:**

1. **Declarations and database core attributes**
2. **A tutorial of GoSynthetic**
3. **Additional Tables**
   1. S-Table 1: Association counts and data sources.
   2. S-Table 2: Processes involved in reaction to light (“Table view” of GoSynthetic)
4. **Research applications**

We give here details to two examples mentioned also in the manuscript:

**a) GoSynthetic results on processes around “Tryptophan” in *E. coli***

**b) GoSynthetic results on processes around “light sensing” in *Homo sapiens*.**

# Declarations and database core attributes

• GoSynthetic is freely available to all via the web without the need to register or login.

• GoSynthetic will be maintained under the same URL for at least 5 years after the publication data (actually we expect even growing demand as synthetic biology takes up more and more momentum)

• All underlying data are available, after acceptance we will also offer the primary data for download. The download will be integrated in the URL of GoSynthetic.

• We include here a supplementary file listing the **database core attributes in accordance with the BioDBcore** standards (http://biocurator.org/biodbcore.shtml).

- Database name : GoSynthetic database
- Main resource : http://gosynthetic.bioapps.biozentrum.uni-wuerzburg.de
- Contact information (e-mail; postal mail)
- Prof. Thomas Dandekar: [dandekar@biozentrum.uni-wuerzburg.de](mailto:dandekar@biozentrum.uni-wuerzburg.de)
- Postal Address: Bioinformatics, Biocenter,
  University of Wuerzburg Am Hubland,
  D-97074, Bayern, Germany
- Date resource established (May 25, 2008)
- Conditions of use
- Free and publicly open
- Scope: data types captured, curation policy, standards used
- publication
- protein interactions
- Standards: MIs, Data formats, terminologies
- GO terms
- Novel established module terms
- Taxonomic coverage
- *Candida albicans*
- *Escherichia coli*
- *Listeria monocytogenes*
- *Rattus norvegicus*
- *Arabidopsis thaliana*
- *Gallus gallus*
- *Saccharomyces cerevisiae*
- *Bos taurus*
- *Danio rerio*
- *Homo sapiens*
- *Mus musculus*
- *Schizosaccharomyces pombe*
- *Caenorhabditis elegans*
- *Drosophila melanogaster*
- *Pseudomonas aeruginosa*
- *Staphylococcus aureus*
- *Vaccinia virus*
- Data accessibility/output options
- Plain-text
- HTML
- Celldesigner xml input/output
- Data release frequency
- Monthly updated
- Versioning period and access to historical files (not provided)
- Documentation available
- Supplmental files and tutorial online available
- User support options
- Authorized user support
- Data submission policy
- free and authorization
- Relevant publications
- Resource's Wikipedia URL
- Tools available

# A Tutorial of GoSynthetic workbench

The aim of the GoSynthetic is to transfer biological processes and functions of different organisms into technical processes. These processes are subsequently divided into smaller subunits – so-called modules – in order to dissect and simplify the process. The simplification is a prerequisite for the generation and manipulation of new biological processes.

GoSynthetic includes 17 model organisms including eukaryotes, prokaryotes and viruses. All the data described are additionally available as an easy to use synthetic biology workbench in the internet under the following link:

[http://gosyn.bioapps.biozentrum.uni-wuerzburg.de](http://gosyn.bioapps.biozentrum.uni-wuerzburg.de/index.php)

A statistical survey displays the distribution of biological modules within different organisms in an almost complete list of literature references (according to PubMed) exclusively in technical literature (IEEE journals) versus synthetic biology literature and between COG and Go classification.

Select organism of interest

Module tree


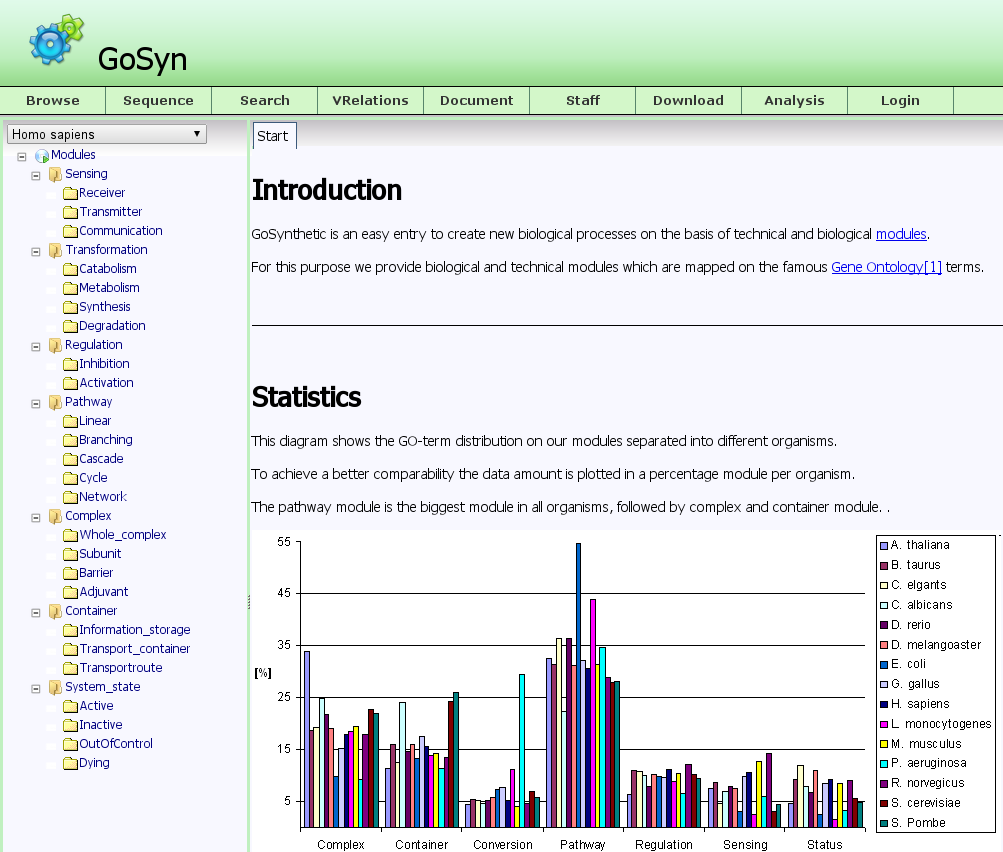
**Figure T1:** Startpage of GoSynthetic: Shows a statistical overview and gives access to the module tree.

All processes and functions are assigned to 30 modules. Within GoSynthetic those modules are described with clear examples and visualisations. Moreover the assignment between technical terms and biological modules is provided.


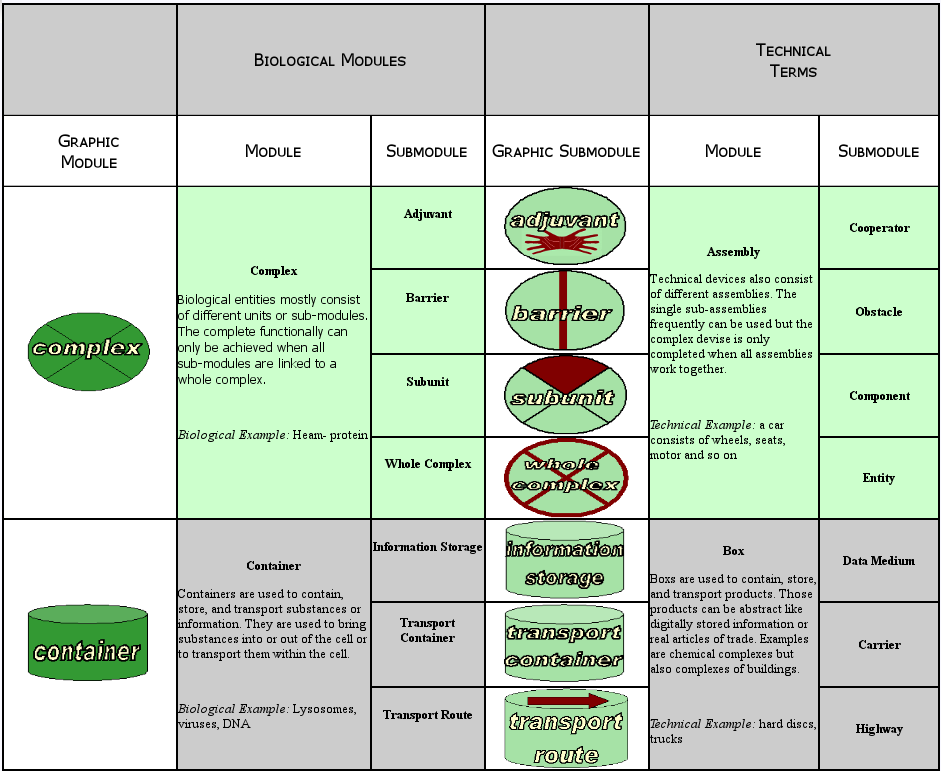


**Figure T2:** GySynthetic provides a detailed list about all modules, short descriptions with all sub-modules and practical examples in biology and technology.

Starting GoSynthetic user can visit the hierarchical module tree with description, corresponding proteins, mesh-terms and COG identifiers. The user can select the desired organism (man, mouse, *E. coli*), 7 different main modules and 23 sub modules. For the chosen organism and module, a separate page is provided with a description of the module, a list of MESH-terms and a table with the corresponding GO-terms, proteins and COG families. Further information and sequences are available as database cross-links.


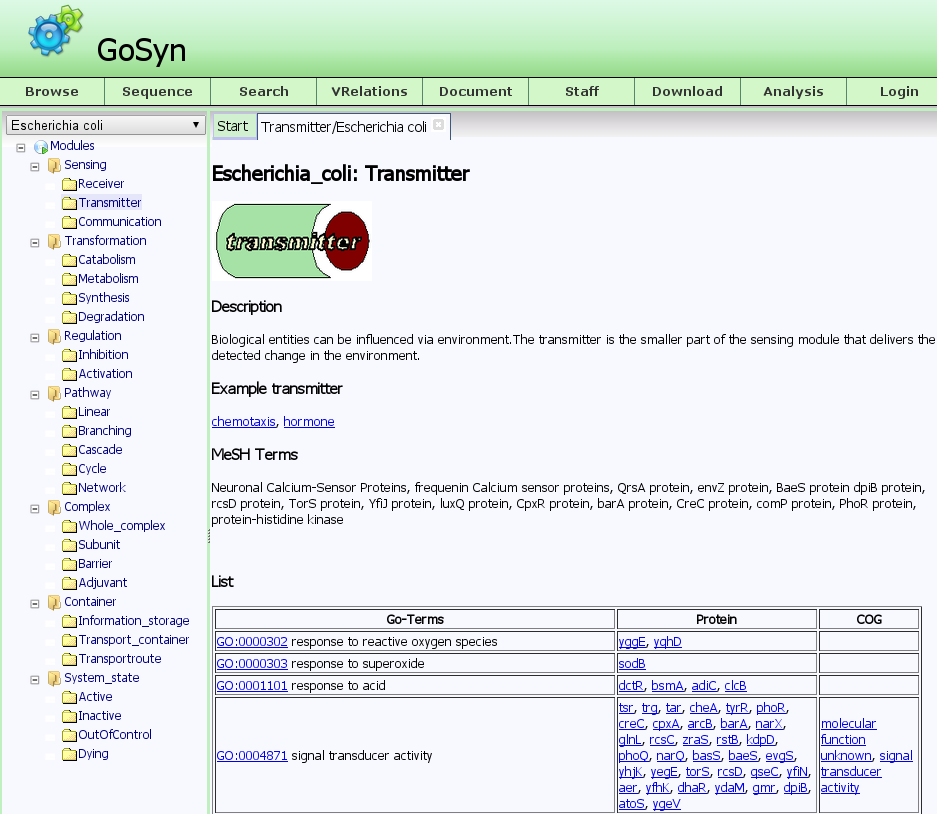


Module tree

Module

tree

Organism

GO-list Protein COG descriptions

**Figure T3:** Module page for one module in one organism. Those pages contain information about the module, the available GO-annotations, proteins and COG families.

Note that our classification compares various different methods, e.g. according to GO-Terms (middle), *Escherichia coli* base (middle right) but also clusters of orthologous groups (very right, scroll down software page for further COGs). The mapping of the modules was optimized so that for each classification possibility a maximum of coverage regarding processes and functions could be achieved.

For an easier access, four different search scenarios are applicable, either based on keyword terms or on process search. The results are visualized by text-highlighting functions, which are supported by graphical statistics and interaction networks to other partner proteins.

1. *Keyword-search*: is an AMIGO like search. The keyword search is resulting in a list of all modules which contain GO-terms with the search-keyword highlighted in red. The keyword search is trivial but useful for GO terms searches.
2. *Process-search*: is a search for biological and technical processes. Based on the buzzword entered, a regular keyword search is performed in the background. For the identified GO-terms all proteins connected to these processes are selected. The modules of the protein’s functions are presented as results. The result is provided as text information and as graphical pie charts (press the pie chart botton; first the different module types involved are given, then pie charts represent their relative importance). One pie-chart provides an overview over all modules while individual pie-charts are additionally shown per module. In addition, the detailed number summary is provided below the figure. Additionally the result is also available as table.
3. *Relation-search*: is a relationship search module for interested term in a specified organism, e.g., *Homo sapiens.* GoSynthetic will search through all the involved proteins and calculate their relationships, eventually painted them in a network. A database IntAct is used as reference.
4. *Sequence-search*: is a rapid search for user-specified protein sequence. Users are allowed to input an interested known or unknown protein sequence, e.g., a human sequence by clicking the “DEMO1” button. Afterwards GoSynthetic will generate a BLAST search. The best match is used to search the involved processes/modules according to its homolog in four organisms, i.e., *Homo sapiens, Bos taurus, Escherichia coli and Staphylococcus aureus*. As result, the suggested annotation is provided, and all the matching processes including the Gene ontology ID and their descriptions are listed in a table. Following the result table, the involved modules are counted and painted as pie-charts; in addition, the detailed module numbers are also shown.

To clarify how to use the different search types in the following they are listed will all their input data and results. Screenshots will bring further information.

***a) Keyword search example:***

Entered keyword: glycolysis

Selected organism: *Homo sapiens*


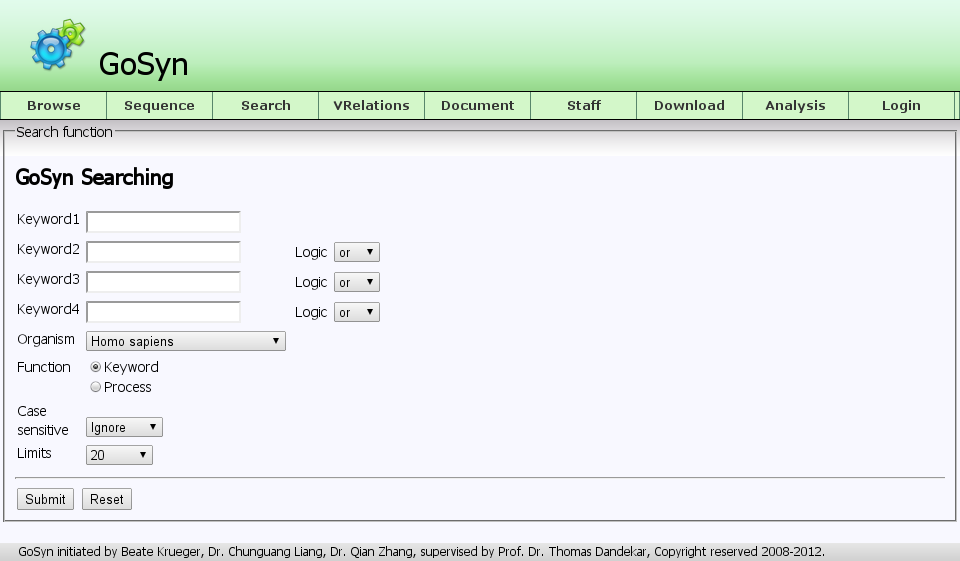


(A1) Enter search-item, and Specify logic operator (or, and, not).

(A2) Select organism

(A3) Select keyword search

(A4) Press submit query

**Figure T4a:** Entry mask for keyword search.

(A1) Enter search item in the input mask, and specifiy its logic operator (or, and, not)

(A2) Select the organism from the combo box

(A3) Select keyword search which is the first radio button, when necessary, choose the threshold, limits and whether case-sensitive.

(A4) Submit the query by clicking on the button

As a result all modules that can be found for glycolysis in *Homo sapiens* are presented as an interactive list. In this case the modules Activation, Pathway and Regulation can be found.

The modules can be opened by the Preview function which shows the module tree page for the selected organism.

**Figure T4b:** Result page for keyword search.


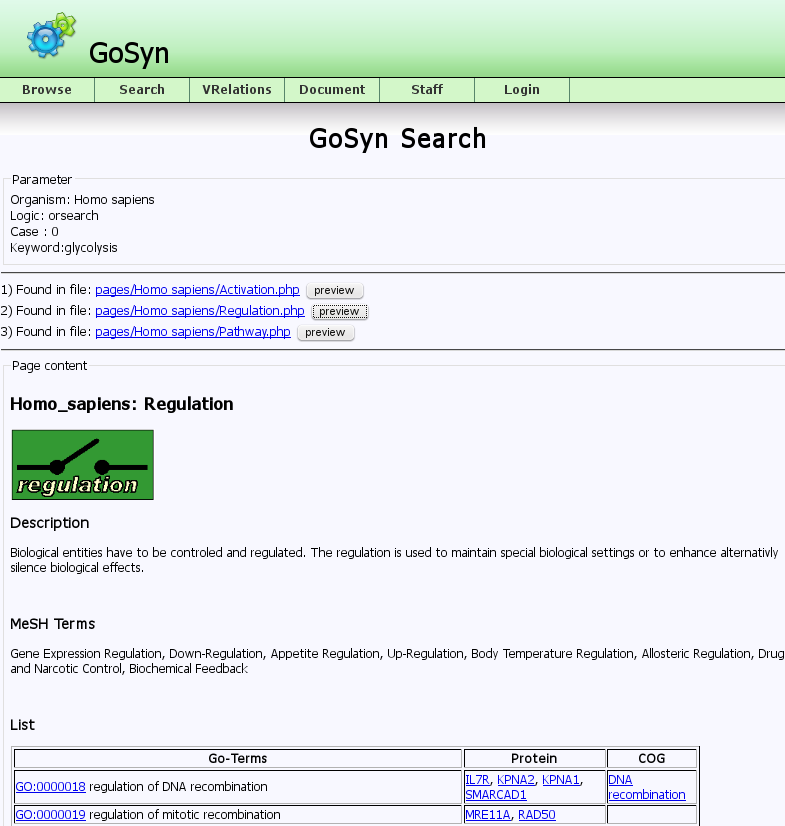


(B1) Search items

(B2) List of all modules containing the keyword

(B3) Click on “preview” to read the detailed module information

(B1) The result pages shows the entered search items

(B2) Additionally a list of all modules containing the keywords is listed.

(B3) By clicking on the preview button the module page for the organism is displayed containing some general module information, interesting Go-terms, proteins and COGs.

***b) Process search example:***

Data have to be inserted similar to the keyword search.

Investigated process: Glycolysis

Selected organism: *Homo sapiens*

(A1) Enter search item in the input mask

(A2) Select the organism from the combo box

(A3) Select process search which is the second radio button

(A4) Submit the query by clicking on the button

Result: The upper bigger pie-chart shows the main module pie-chart. The glycolysis in *Homo sapiens* is dominantly a strongly regulated pathway (largest sections of the pie-chart).

Additional below the smaller sub-module pie charts are shown. They describe further information about the main module. For example the regulation is mainly positively regulated and is linear within an active cell.


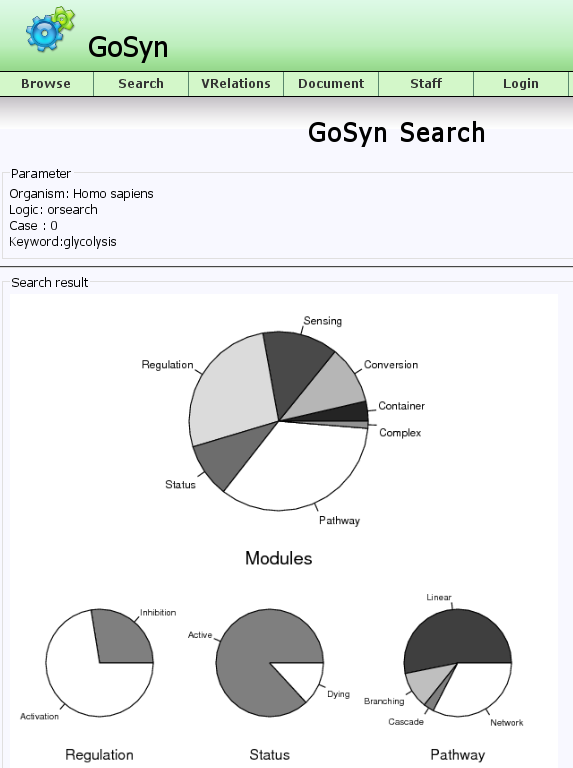


(B2) Main module pie-chart and the corresponding sub-module pie-charts (B3)

(B1) search entries

**Figure T5:** Result page for keyword search.

(B1) The result pages shows the entered search items

(B2) Additionally a pie chart containing the main-module distribution for the selected search item

(B3) Below the main pie-chart, all processes of the sub-modules are displayed as piecharts. To get a more detailed list of processes, a summarized list is given below the figure. In addition, users can also query them using GoSynthetic keyword search function and check all the related information. Furthermore, there is a VRelation option (Visual. Relation option) for studying their relationships in a network.

***c) Relation-search (VRelations):***

A connection with the interaction database IntAct provides the opportunity to identify interactions and connections to other biological processes including graphical network visualization. Direct translation of processes and modules in biological organisms and protein interaction networks may be retrieved including gene ontology searches. This allows easy construction of own novel networks containing desired technical or biological modules. The GO synthetic software can be used as an intuitive and creative tool for process engineering as well as for the design of new experiments.

Investigated process: Glycolysis (directly by “DEMO1” button)

Selected organism: *Homo sapiens*


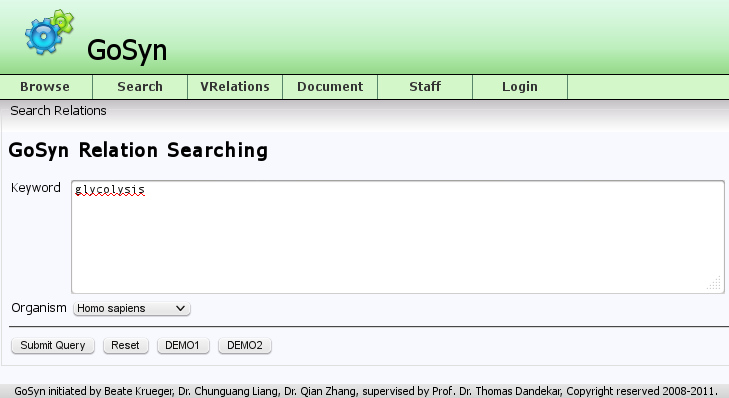


(A1) Enter keyword

(A2) Select organism

**Figure T6a:** Entry mask for relation search.

(A1) Enter keyword

(A2) Select the organism

As a result a list is provided which contains all modules that have been identified within the process. The list additionally displays the related processes and the connection between these processes. Proteins within the process can be added.

The terminology can be switched between technical and biological modules.

Visualization shows the interaction between the proteins in the process based on the IntAct database. Activating proteins are highlighted in orange, while inhibitory proteins are highlighted in blue.

(B2) Add proteins to module


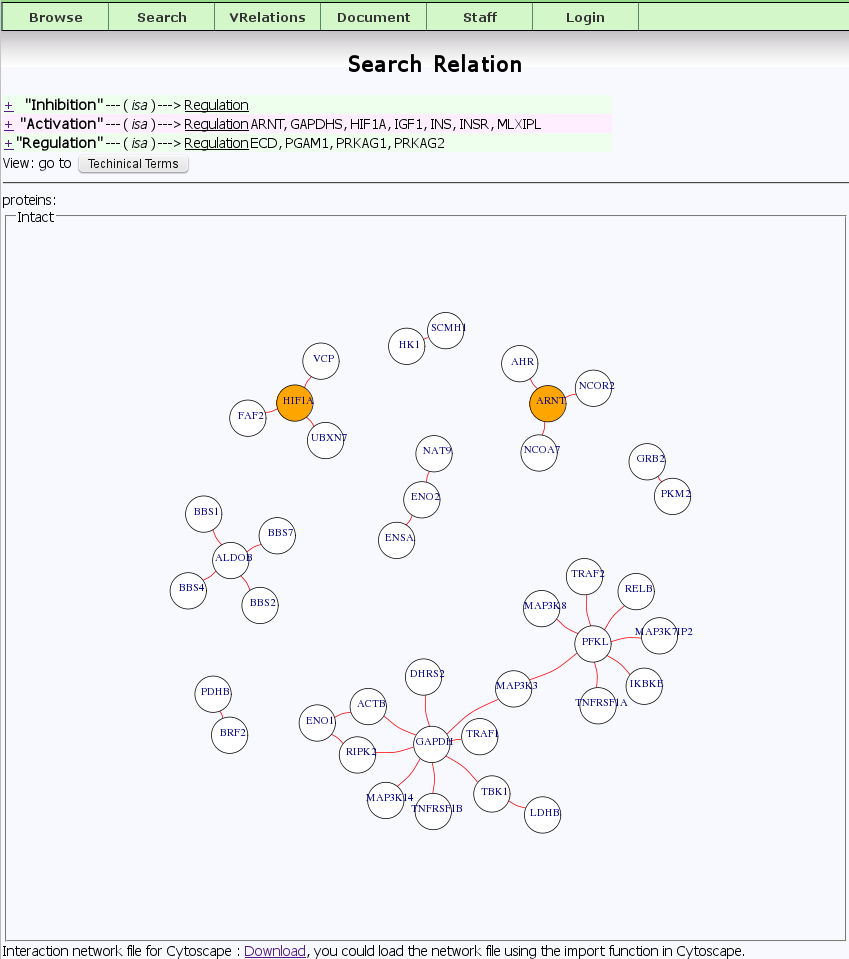


(B1) List of all modules used by the process with the corresponding proteins

Download the model file for Cytoscape

(B3) Found proteins are presented within a network. Interacting proteins are connected with red lines

**Figure T6b:** Result page for relation search.

(B1) As a result a list of all modules containing the search item is displayed

(B2) Proteins involved in the process can by displayed by clicking on the plus symbol

(B3) An interaction network for the involved proteins is displayed. Red lines between the proteins show direct interactions.

The visualized result and protein assembly can be readily exported into the open-source Cytoscape platform (Cline et al., [2007](http://www.ncbi.nlm.nih.gov/pubmed/14597658)) for the dynamic visualization of molecular interaction networks. It is also possible to modify the network further and assign additional data (gene expression metrics; any other desired data sets) and/or detailed functionalities to each network element.

Together with biological expert analysis (such as literature search and interactom search) even more extended examples can be explored with the two-level classification (see supplement information and results).

Another example for using the relation-search is studying the chemotaxis for one human protein (GTPase-HRas partial sequence; indicated by the blue arrow (HRAS); GenBank accession number: CAG47067; this is an example for either an unknown or partial sequence or as part of a synthetic biology engineering construct) using the GoSynthetic sequence-query function (“Sequence” in the menu-bar).
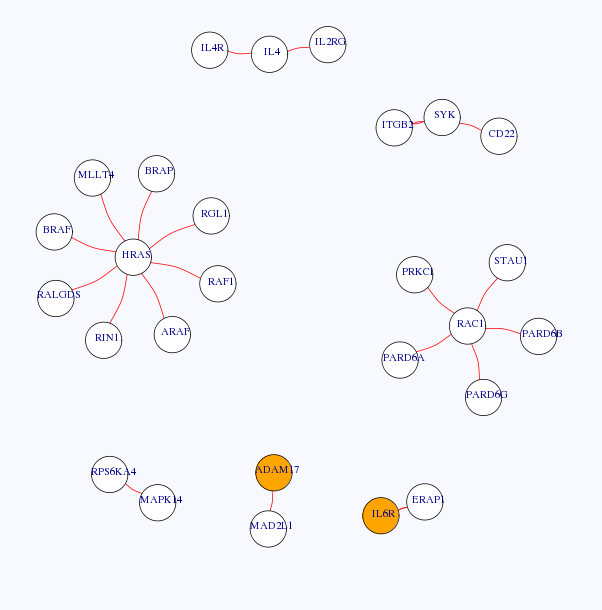


**Figure T6c:** Result page relation search, studying the chemotaxis for one human protein (GTPase-HRas partial sequence).

Orange colour indicates activatory nodes (starting processes, e.g. IL6 receptor or zinc protease ADAM7), interactions according to the stored data (including hand curated information from HPRD) are shown in red. Chemotaxis is depicted here to rely on seven independent clusters of protein interactions. However, the further higher level connections and overarching processes can also be analyzed with GO-synthetic, for instance to plan protein expression experiments influencing chemotaxis (going up the hierarchical classifications of functions, e.g. according to GO-terms). Furthermore, we offer the option to look for cell-specific adaptations for chemotaxis, thus leukocytes exhibit chemotactic behaviour and design in synthetic biology (e.g. block chemotactic receptors, an old way would be using heparin; choose term “leukocyte” in GoSynthetic and analyze further; key functional proteins according to the module information are shown; interaction clusters calculated according to HPRD information appear in VRelation search).

***d) Sequence search example:***

Investigated process: a human protein sequence by clicking “DEMO1” button

Selected organism: *Homo sapiens*


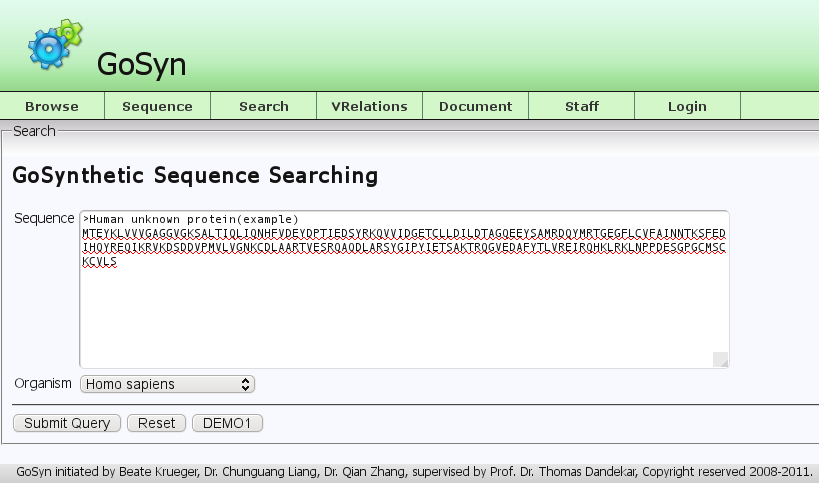


(A1) Enter a protein sequence in FASTA format

(A2) Select organism

**Figure T7a:** Entry page sequence search.

(A1) Enter a protein sequence in FASTA format

(A2) Submit the query by clicking on the button

The best protein match for the sequence is selected and analyzed via GoSynthetic. As a result the found protein is listed, together with the corresponding GO-annotations and function pie-charts (an overview of involved cellular functions the process is connected to, similar to those of the process search).


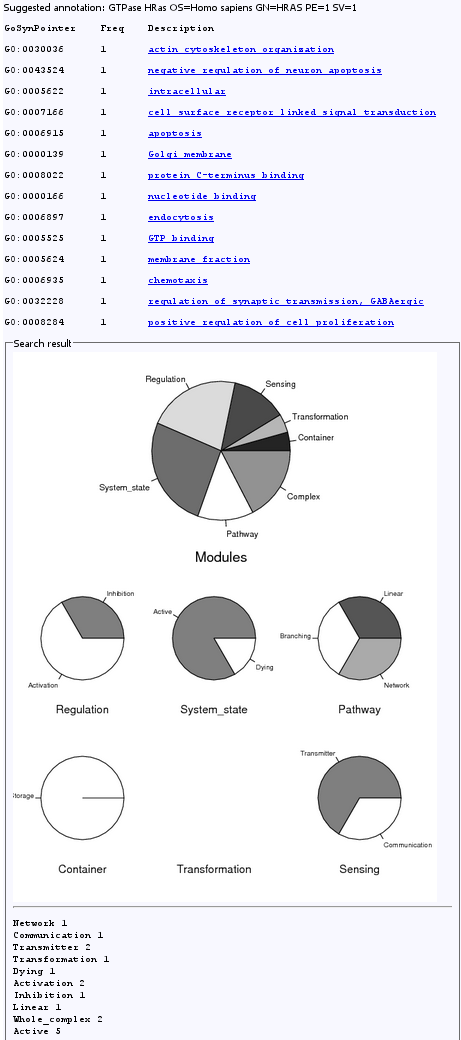


(B1) Found protein

(suggested annotation)

(B2) The function of the protein is described in list, for *Homo sapiens*, the interaction information can be immediately queried by these links. For other organisms, the corresponding information can be obtained using “VRelations” search-module.

(B3) A piechart for the main modules is shown below, similar to the result page of the process search

(B4) Table of all found modules for the proteins and the frequencies

**Figure T7b:** Result page sequence search.

(B1) The protein from the best BLAST result is displayed and described

(B2) The function of the protein is described by the GO annotation

(B3) A pie chart for the main modules is shown below, similar to the results page of the process search.

(B4) Table of all found module types for the proteins. Relative proportions are given.

Figure T8. Analysis of Process “Glycolysis” in 7 organisms.


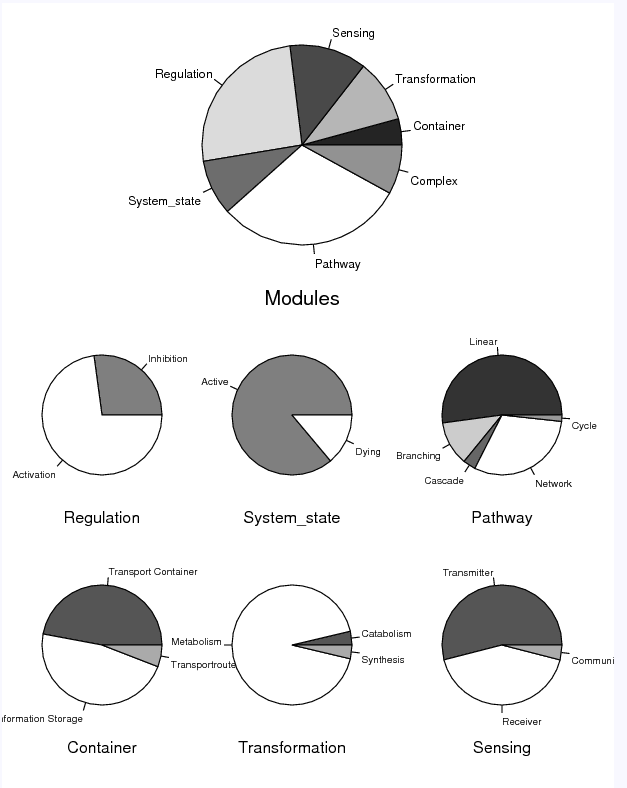
**Fig T8a. Analysis of process “glycolysis” in *H. sapiens*.**


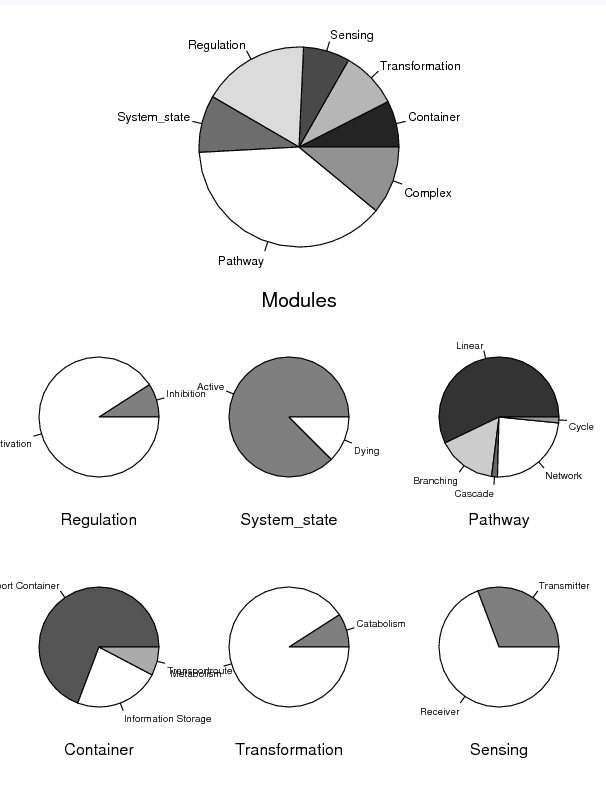
**Fig T8b. Analysis of process “glycolysis” in *B. taurus*.**


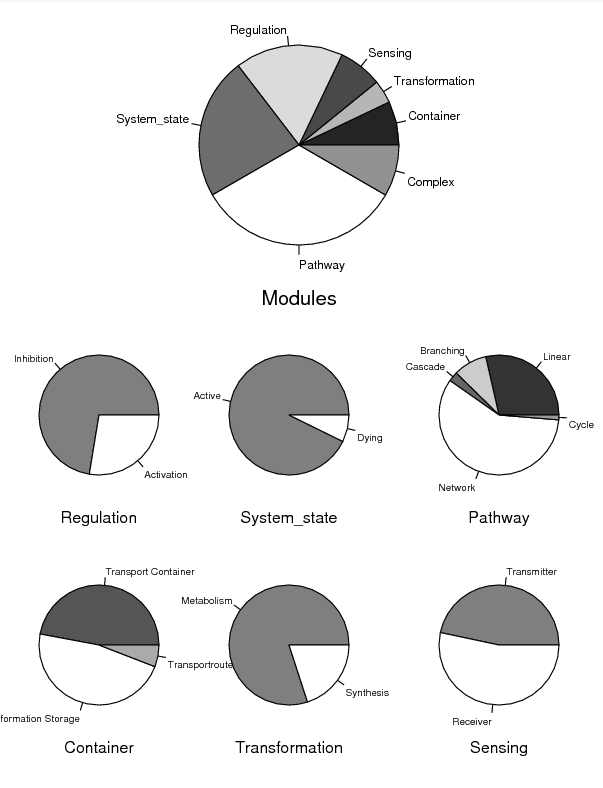
**Fig T8c. Analysis of process “glycolysis” in *D. melanogaster.***


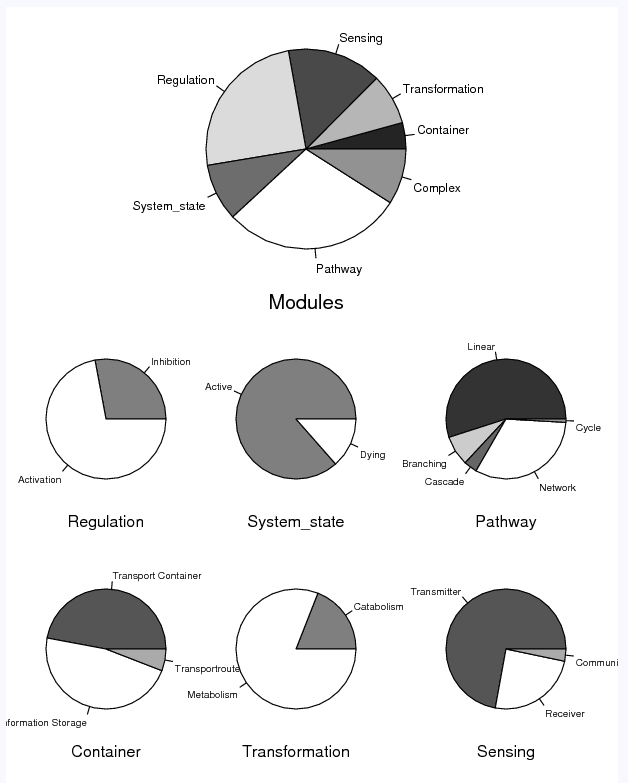
**Fig T8d. Analysis of process “glycolysis” *R. norvegicus*.**


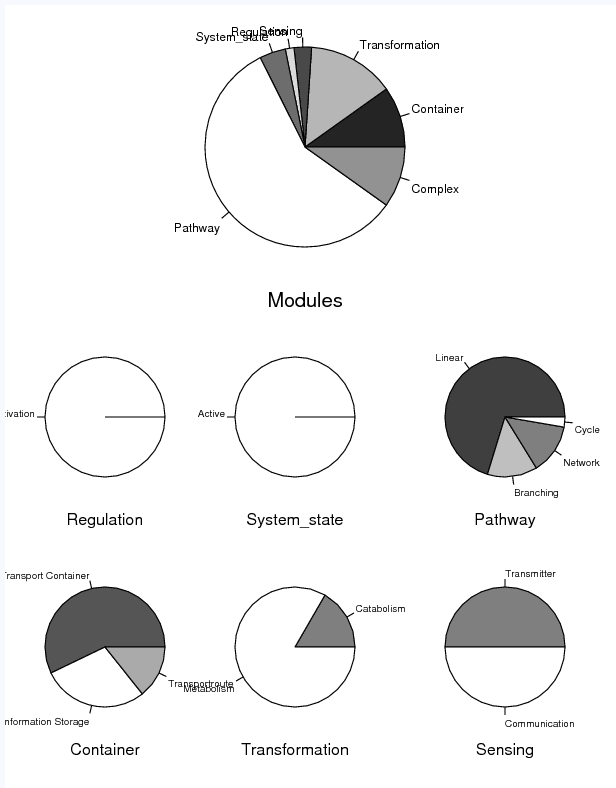
**Fig T8e. Analysis of process “glycolysis” in *E. col*i.**


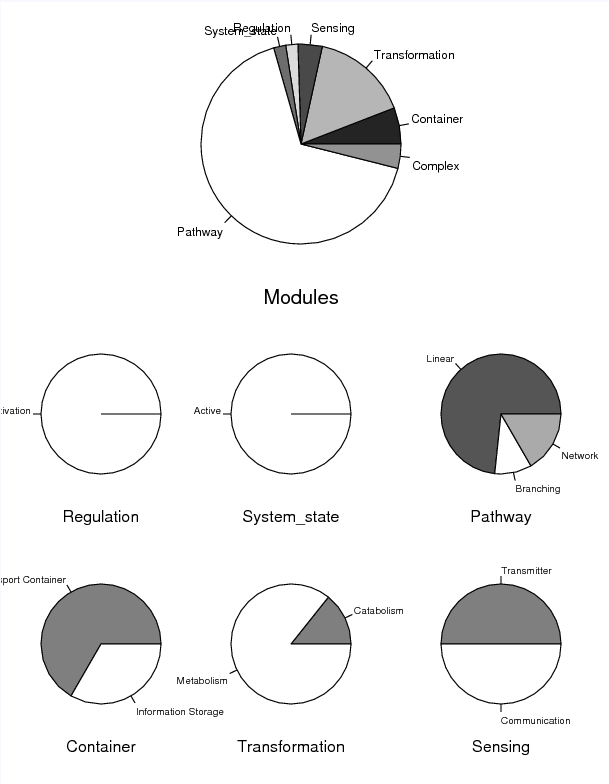
**Fig T8f. Analysis of process “glycolysis” in *S. auerus***

**
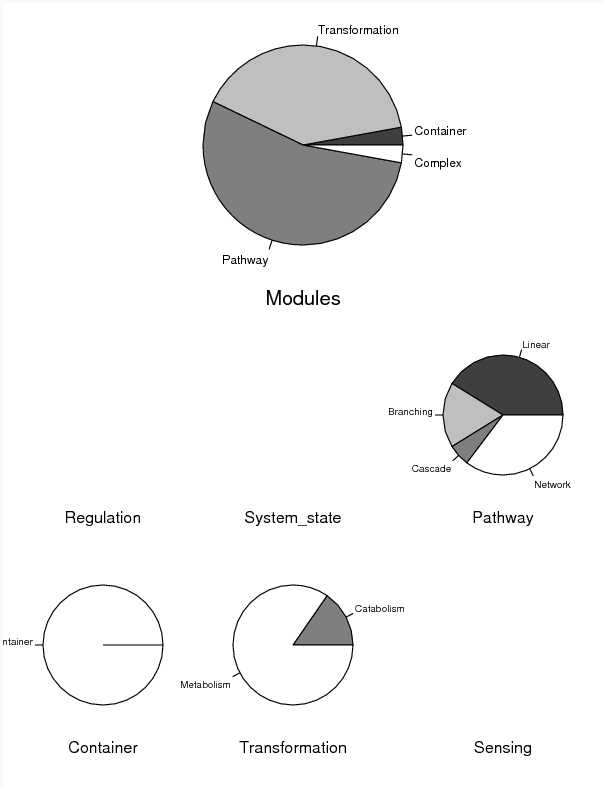
**

**Fig T8g. Analysis of process “glycolysis” in *P. aeruginos*a.**

**
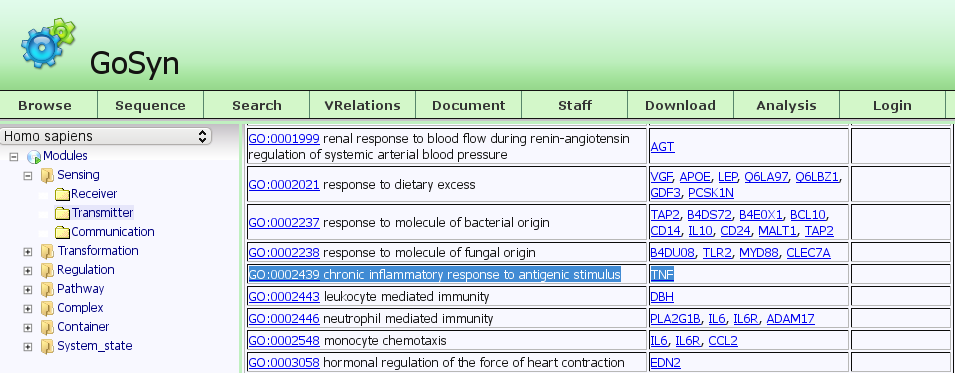
**

**Fig T9. Crosslinks (blue) and keyword in processes.**

External crosslinks are given in the second column, e.g., TNF, for protein queries. The GO external link is in the first column, e.g, GO:00002439, they are also available for external querying the GO terms in Amigo. A keyword is best chosen as a noun or a process adjective stored in the hierarchical process tree of GoSynthetic (so according to gene ontology, COG/KOG or bio-bricks), in the example shown “inflammatory” was combined with “process” in the keyword searching module.


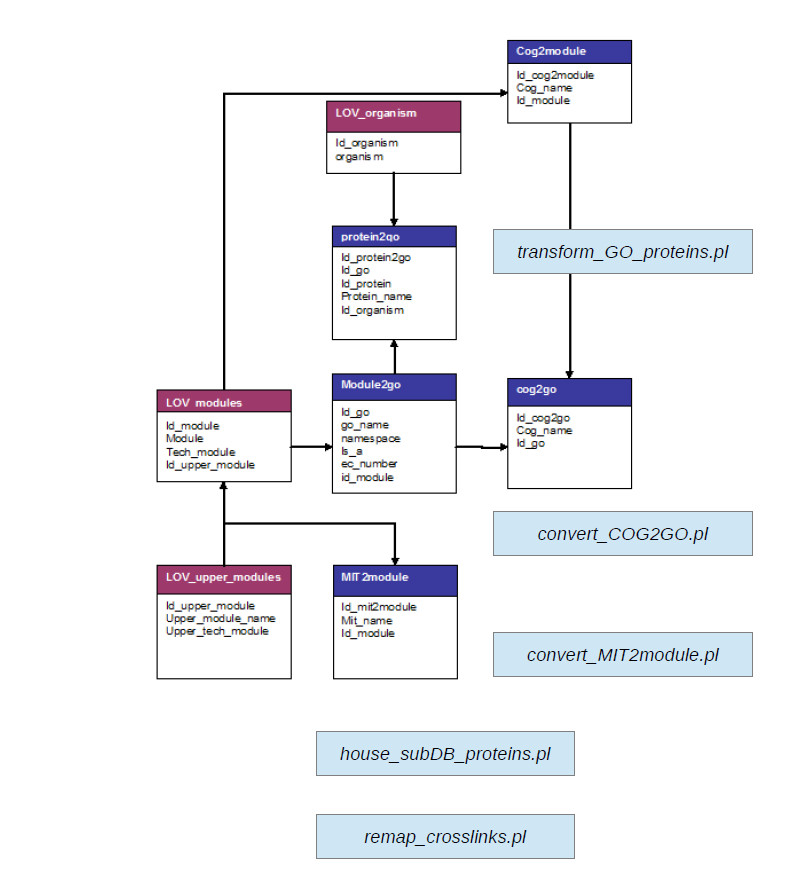
**Fig T10. Maintenance and update of GoSynthetic database.**

Maintenance and update of GoSynthetic database have to be done routinely and follow the entity relationship diagram (Figure 7 of manuscript). We update organism-specific identifiers and their annotation every three months according to the latest public available version. This involves all entities as shown above. Importantly, bridging information e.g., COG2GO and other searching indexes are automatically rebuild using core update Perl scripts (the four major ones are shown in blue rectangles) once the new organism-specific data have been incorporated. Manual curation of module classes (both for biological- and technical- terms) are frequently carried out as well.

# Tables

**S-Table 1. Association counts and data sources ^1^**

|  | *E. coli* | *S. aureus* | *H. sapiens* | *S. cerevisiae* | *D. melanogaster* | Total |
| --- | --- | --- | --- | --- | --- | --- |
| **GO** |  |  |  |  |  | 28544 |
| **Proteins** | 19812 | 18291 | 191199 | 60941 | 55869 | 345662 |
| **COGs** |  |  |  |  |  | 1102 |
| **Interactions** | 7863 | 6732 | 3164 | 11563 | 6524 | 35846 |
| **Cross-links** |  |  |  |  |  | 1068441 |

^1^The table shows the amount of data processed for GoSynthetic, listed separately for important model organisms as well as for the database as a whole.

**S-Table 2. Table-view for processes involved in reaction to light.^1^**

| Module | GO_ID | Description | *A.*  *thaliana* | *H. sapiens* |
| --- | --- | --- | --- | --- |
| Catabolism | 0010304 | PSII associated light-harvesting complex II catabolic process | 6 |  |
| Complex | 0009517 | PSII associated light-harvesting complex II | 3 |  |
| Complex | 0030076 | light-harvesting complex | 20 |  |
| Inhibition | 0010362 | negative regulation of anion channel activity by blue light | 3 |  |
| Network | 0009765 | photosynthesis, light harvesting | 5 |  |
| Network | 0009768 | photosynthesis, light harvesting in photosystem I | 5 |  |
| Network | 0009769 | photosynthesis, light harvesting in photosystem II | 7 |  |
| Network | 0019684 | photosynthesis, light reaction | 16 |  |
| Receiver | 0009584 | detection of visible light |  | 1 |
| Receiver | 0009882 | blue light photoreceptor activity | 4 |  |
| Receiver | 0009883 | red or far-red light photoreceptor activity | 3 |  |
| Regulation | 0042548 | regulation of photosynthesis, light reaction | 1 |  |
| Sensing | 0050953 | sensory perception of light stimulus |  | 4 |
| Transmitter | 0007603 | phototransduction, visible light |  | 7 |
| Transmitter | 0009416 | response to light stimulus | 117 | 15 |
| Transmitter | 0009583 | detection of light stimulus |  | 1 |
| Transmitter | 0009637 | response to blue light | 21 |  |
| Transmitter | 0009639 | response to red or far red light | 37 |  |
| Transmitter | 0009642 | response to light intensity | 6 | 1 |
| Transmitter | 0009644 | response to high light intensity | 37 |  |
| Transmitter | 0009645 | response to low light intensity stimulus | 3 |  |
| Transmitter | 0009646 | response to absence of light | 7 |  |
| Transmitter | 0009785 | blue light signaling pathway | 8 |  |
| Transmitter | 0010017 | red or far red light signaling pathway | 21 |  |
| Transmitter | 0010018 | far red light signaling pathway | 3 |  |
| Transmitter | 0010114 | response to red light | 35 |  |
| Transmitter | 0010161 | red light signaling pathway | 7 |  |
| Transmitter | 0010201 | response to continuous far red light stimulus by the high-irradiance response system | 1 |  |
| Transmitter | 0010202 | response to low fluence red light stimulus | 1 |  |
| Transmitter | 0010203 | response to very low fluence red light stimulus | 3 |  |
| Transmitter | 0010218 | response to far red light | 22 |  |
| Transmitter | 0010244 | response to low fluence blue light stimulus by blue low-fluence system | 1 |  |
| Transmitter | 0043481 | anthocyanin accumulation in tissues in response to UV light | 4 |  |
| Transmitter | 0050908 | detection of light stimulus involved in visual perception |  | 3 |

^1^The Table shows a listing of all modules and GO-terms and the number of involved proteins per organism comparing *H. sapiens* to *A. thaliana* (“Table view” GoSynthetic, biological classification; further functional classifications, e.g. according to COGs, are also available). The table was manually edited to save space, for full search results query the web version of the database as described in the results. Interesting differences between both organisms as well as the richness of functions involved, even for *H. sapiens*, can now be analyzed and exploited in detail for synthetic biology (option: switch the software to the technical view, listing all technical modules), protein engineering (where both views are interesting) or molecular biology (exactly the view shown here).

# Research applications

**a)** GoSynthetic results on “Tryptophan” in *E. coli*

As an example for engineering a metabolic process, we use the tryptophan in *Escherichia coli* and analyze it with the help of process and relation search. Shown are piecharts of processes (right), and their exact relations (left), mode with interactions (left bottom), detailed process counts for involved processes (right, bottom). For detailed procedures how to obtain each please see the tutorial.


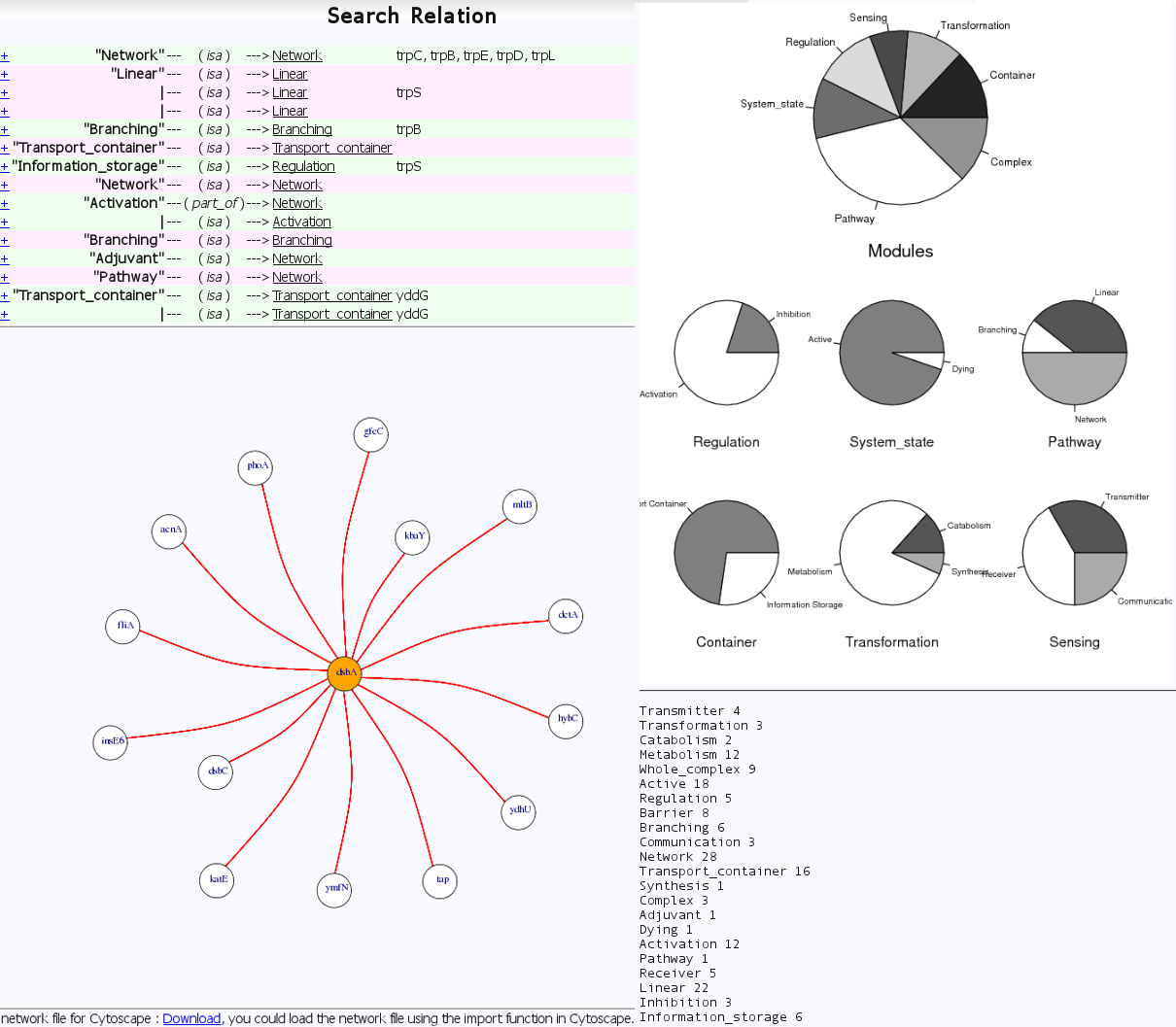
**Figure S1:** Result of GoSyntetic relation search and process search for tryptophan in *E. coli.*

**b**) GoSynthetic results on “reaction to light” in *Homo sapiens*.

GoSynthetic allows further planning of such artificial design experiments taking input from various organisms. We illustrate this for *H. sapiens*. Here we show key results for process and relation search (left) with the process term combination “reaction” AND “light” (right) and detailed list (right, bottom). This is an edited composite picture. Full output is several pages and should be done as described in the results.


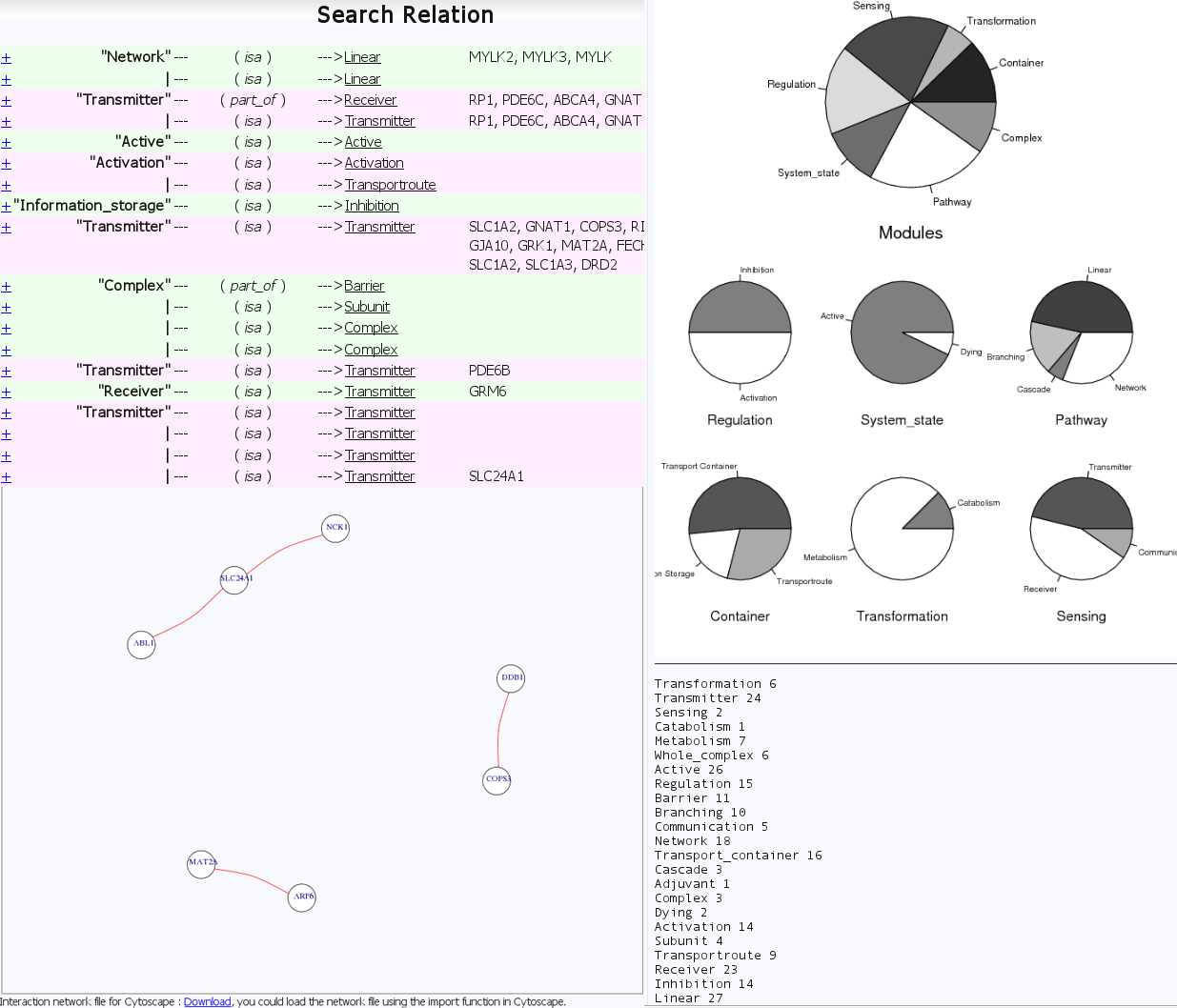


**Figure S2:** Result of GoSyntetic relation search and process search for reaction to light in *H. sapiens.*
