# Supplementary material for: GoSynthetic database tool to analyse natural and engineered molecular processes
Source: Database (Oxford). 2013 Jun 27;2013:bat043. doi: 10.1093/database/bat043 (PMC3694605; doi:10.1093/database/bat043)
Supplement: Supplementary Data [file supp_bat043_Database_GoSynthetic_supplmaterial-file2-f.doc]

### Database Tool

**GoSynthetic database tool to analyze natural and engineered molecular processes**

**(supplementary file 2)**

Chunguang Liang^1,§^, Beate Krüger^1,§^, Thomas Dandekar^1, 2,*^

^§^ both authors contributed equally to this work

^1^dept of bioinformatics, biocenter, Am Hubland, University of Würzburg, 97074 Würzburg, Germany

^4^European Molecular Biology Laboratory, Meyerhofstr. 1, 69012 Heidelberg, Germany

^*^corresponding author

# Module establishment for GoSynthetic

## 1. Basic considerationsns

In general, classification is the study or concern about what kinds of things exist - what entities there are in the universe. We are here specifically concerned with the problem of establishing an ontology useful both for biology and engineering and establish comparabilities. Some of the challenges are:

- To share common underanding of a common structure both for biology and engineering
- To enable reuse of discipline-specific knowledge as well as between different technologies and natural sciences
- To make discipline-specific assumptions explicit
- To separate discipline-specific knowledge from operational knowledge apparent from the ontology and its specific hierarchical classification

In practical terms, developing a classification includes:

- defining terms for classification
- arranging them in a taxonomic (subclass–superclass) hierarchy
- defining slots and describing allowed values for these slots
- filling in the values for slots for instances.

To define this specific classification several steps were necessary and several ideas were combined.

## 2. Relationship of the modules

The starting point of the classification uses typical defining terms for life. The seven modules cover the basic definition of life stressing emergent and system properties. In Table 1 in the manuscript they are summarized, examples are given in S2-Table 1:

Various definitions of life all converge and agree that living beings undergo metabolism (pathway modules), maintain homeostasis (regulation module), possess a capacity to grow (transformation module), respond to stimuli (sensing module), reproduce (system state module) and, through natural selection (operating on different cellular complexes), adapt (as complete entities / container module) to their environment in successive generations. However, from a design and engineering perspective the emergent features of each classification module are the key aspect as well as their inherent options for control and regulation (Table 1b in the manuscript). Our new classification considers also GO terms, COG/KOGs and MIT Biobrick functions but stresses key functions and modules an individual protein may participate in. In general a protein has several functions (in gene ontology for instance molecular function, cellular compartment and cellular processes it participates in). Also in our new classification in general a protein participates in multiple modules (or dimensions of emergence typical for the processes it is involved in) while the submodules of our classification (S2-Table 2) stress key system changes which can occur within the module.

**S2-Table 1. Modules in GO-Synthetic**

| **Molecular Biology Module** | **Engineering Term** |
| --- | --- |
| **Complex**  Biological entities mostly consist of different units or sub-modules. The complete function can only be achieved when all sub-modules are linked to a whole complex.  *Biological example: Heme- protein* | **Assembly**  Technical devices also consist of different assemblies. The single sub-assemblies frequently can be used but the complex devise is only completed when all assemblies work together.  *Technical example:* a car consists of wheels, seats, motor and so on |
| **Sensing**  Biological entities can be influenced via environment. Therefore they need sensors that notice changes in the environment and cause an effect. Possible observed parameters could be temperature, nutation or osmosis.  *Biological example:* Two-component systems. | **Detection**  Detectors respond to sensors for changes in the environment; for examples changes in temperature, pressure or humidity.  Electronic detectors. A simple detection system for example is a central heating with thermostat or an air conditioning system. |
| **Regulation**  Biological entities have to be controlled and regulated. The regulation is used to maintain special biological settings or to enhance alternatively silence biological effects. Consequently all biological processes are regulated.  *Biological example:* Blood coagulation is positive regulated | **Control System**  Control system is used to maintain special settings or to increase alternatively reduce some effects.  A well known example for a control system is an electronic amplifier such as a transistor. Negative feedback regulation includes temperature control by small or macroscopic sensors. |
| **Pathway**  Biological processes are organized within pathways.  A pathway is a set of reactions to manage, command, or regulate the behavior of systems directly or indirectly. These systems can be linear, branching, cyclic or cascading.  *Biological example: The* glycolysis is a linear pathway with a branching point. The citrate cycle is a cyclic pathway. | **Process Structure**  A process structure describes the way a process uses or products are transported.  Those systems can be linear, non linear, cyclic or cascading.  Process structures in engineering describe for example the cycle between generator and consumer or the linear transmission of light in a fiberglass cable or optoelectronic device). |
| **Transformation**  In nature waste is rarely produced. Normally, products are recycled into other products. Even energy is converted in different forms of energy. Consequently transformation can deal with energy production or consumption or just with the recycling of biological products.    *Biological example:* Food is converted into energy in form of ATP | **Conversion**  In industrial production conversion is a general topic. Materials have to be recycled and energy has to be produced or transformed in a different type of energy.  *Technical example:* Conversion of stored energy in a condenser into current in a circuit. |
| **Container**  Containers are used to contain, store, and transport substances or information. They are used to bring substances into or out of the cell or to transport them within the cell.  *Biological example:* Lysosomes, viruses, DNA | **Box**  Boxes are used to contain, store, and transport products. Those products can be abstract like digitally stored information or real articles of trade. Examples are chemical complexes but also complexes of buildings.  *Technical Example*: hard discs, trucks |
| **System State**  Biological entities can adapt to different states within their life cycle. In the beginning they start their life, they multiply and metabolize and at the end of the lifetime they will die. During illness a system can also be out of control  .*Biological example:* cell division is active or apoptosis is dying | **Process Phase**  With the expression process phase the phase of a technical system is meant. A system can be new, waiting, in process, finished or out of order or on/ off and standby. |

After a refinement process (see below) the established modules where as described in S2-Table 1. As each Module (7 types) with all contained submodules (33 in total) is directly corresponding to an engineering term and its sub-classifications a direct translation of biological classification into technical terms is easy possible including a two-level dictionary of corresponding terms. The classification had to be coupled to different processes. GoSynthetic used for this the standard gene ontology terms and started by mapping these to the new two-level classification. The mapping was iteratively refined considering also other classification schemes such as COG terms (S2-Table 4, mapping shown below) and MIT-biobricks (S2-Table 2).

***S2-Table 2. Technical and biological processes covered by synthetic biology BioBricks***

| ***Biological***  ***term*** | ***Engineering***  ***term*** | ***Engineering***  ***Subterm*** | ***Biological***  ***Subterm*** | ***BioBricks*** |
| --- | --- | --- | --- | --- |
| **Complex** | **Assembly** |  |  | Protein domain |
|  |  | Component | subunit | Protein coding sequences/  Ribosome Binding Sites |
|  |  | Cooperator | adjuvant | Conjugation/”Coliroid”  (light sensitive *E.coli*  produced photographs) |
|  |  | Entity | whole complex | Translational units |
|  |  | Obstacle | Barrier | Terminators |
| **Container** | **Box** |  |  | Plasmids |
|  |  | Carrier | transport container | Plasmids backbones |
|  |  | Data medium | information storage | DNA/ Protein coding  sequences |
|  |  | Mobility | transport route | Motility and chemotaxis |
| **Sensing** | **Detection** |  |  |  |
|  |  | Controller | communication | Receivers and senders/  Measurement devices/ |
|  |  | Reporter | transmitter | Reporters/  Measurement devices |
|  |  | Sensor | receiver | Measurement devices  Cell-cell signaling and  quorum sensing |
| **Regulation** | **Control_system** |  |  |  |
|  |  | Reduction | activation |  |
|  |  | Stimulation | inhibition | Promoters |
| **Transformation** | **Conversion** |  |  | DNA recombination |
|  |  | Disassembling | degradation |  |
|  |  | Energy-consumption | anabolism |  |
|  |  | Energy-production | catabolism |  |
|  |  | Production | synthesis | Protein generators/  Protein coding sequences/  Ribosome Binding Sites |
|  |  | Remodeling | metabolism | Biosynthesis |
| **System State** | **Process_phase** |  |  |  |
|  |  | Broken | out of control |  |
|  |  | Off | inactive |  |
|  |  | On | active |  |
|  |  | Standby | dying |  |
| **Pathway** | **Process_structure** |  |  |  |
|  |  | Cascade | Cascading |  |
|  |  | Circulation/ring | Cycle |  |
|  |  | Conveyor/ pipe | linear |  |
|  |  | Meshwork | network |  |
|  |  | Splitting | branching |  |

We derive in this way an own classification, optimized from an engineering perspective. The classification is a bit less rich than GO terms, however, the iterative refinement provides unique assignment of GO terms, of COG terms and of MIT-biobricks to the two-level hierarchy. This is helpful for design experiments as you can directly translate engineering concepts using the two-level classification of technical processes and map them onto or analyze now biological processes in technical terms, using the hierarchical classification. A number of other applications are given and explained in the paper, see results and methods section, stressing engineering and molecular biology experiments and a hierarchical analysis of the involved processes.

However, for unique mapping a number of problems had to be solved. In particular, a biological process in the GO classification is frequently assigned to multiple modules. To provide a simper handling of the classification all the GO terms where only assigned to one module – the most specific modules. That means e.g. TCA (tricarbon acid cycle) is a “pathway” but it is also a “circular pathway” and this is more specific than “pathway” so it was given the more specific property “circle”.

The submodules were established and assigned as the upper modules. Consequently they do also overlap but the terms where always assigned to the most specific module, in particular, regarding protein engineering experiments. The latter is a clear designer perspective and looks more at structure and control aspects of the process, in other words, it is already some distance from the purely biological classification according to key properties of life but this distance allows then to plan experiments more from a designer or engineering perspective with a new, independent classification.Hence we establish here a new classification, inspired by GO-terms, the definition of life and an engineering perspective on designing processes.

For example the process “meiosis” is described with the classification as a pathway, mainly cyclic and branching, and that it works mainly as a “transformation”. This classification fits very well considering that meiosis is the process of cell division and reproduction whereby normal chromosomes are “transformed” into gametes. Of course also other attributes are relevant to describe the meiosis but those were considered as the most important ones. To get to know more about the regulation in meiosis or containing proteins the GoSynthetic homepage provides multiple search-possibilities which facilitate a refined search both in depth as well as regarding specificity.

In addition to these functions, attributes that are important for engineering were added like in electronics the energy transformation or the switching circuit. The seven top modules were specified by sub-modules to achieve a higher specificity. Each sub-module type with all its terms is completely contained in its (parent) module (S2-Table 3). For instance, all processes and terms described in the sub-module type “branching” (right column, fifth line) are contained and belong also to the top level module type pathway (left column, fifth line).

**S2-Table 3. Two level hierarchical classification**

| **Subunits** | **Explanation** |
| --- | --- |
| **Sensing Subunits** | Biological entities can be influenced via environment. They need sensors to notice changes in the environment and cause an effect. |
|  | Transmitter, Receiver, Communication |
| **Regulation Subunits** | Biological entities have to be controlled and regulated. The regulation is used to maintain special biological settings or to enhance or alternatively silence biological effects. |
|  | Activation, Inhibition |
| **Pathway Subunits** | Biological processes are organized within pathways. A pathway is a set of reactions to manage, command, or regulate the behavior of systems directly or indirectly. |
|  | Linear, Branching, Cycle, Cascading, Network |
| **Transformation Subunits** | Energy is converted in different forms of energy. Consequently transformation can deal with energy production or consumption or recycling of biological products. |
|  | Anabolism, Catabolism, Metabolism, Degradation, Synthesis |
| **Container Subunits** | Containers are used to contain, store, and transport substances or information. |
|  | Information Storage, Transport Container, Transport Route |
| **System state Subunits** | Biological entities adapt to different states. In their life cycle they live, multiply, metabolize and die. |
|  | Active, Dying, Inactive, Out Of Control |
| **Complex Subunits** | Biological entities consist of different units or sub-modules. The complete functionality can only be achieved when all sub-modules are linked to a complex. |
|  | Adjuvant, Barrier, Subunit, Whole Complex |

**3. Establishing the classification by iterative refinement**

Once the first draft of the classification was initiated its verification started. Therefore all gene ontology terms were investigated regarding assignment to the established modules and sub-modules. For this assignment the module names and all found synonyms were mapped with a text-mining script to the gene ontology. One gene ontology term can only be assigned to one module in the classification. Removing all these ambiguities removes about half of all ontology terms: The iterative mapping refinement was done focussing exclusively on these remaining unique gene ontology terms, starting from the most specific sub-modules to combine relevance, novelty, and term of interest. For those gene-ontology terms that could not be mapped against the classification new modules or sub-modules where created. The aim was to achieve a high coverage of gene ontology term into the classification.

The establishment of the module hierarchy was achieved by iterative refinement. Some basic modules were mapped against the gene ontology terminology. Considering the remaining terms that could not be mapped to modules or sub-modules new modules were created and the process is then reiterated. This refinement-cycle converged with a coverage of about 95% of all gene ontology terms. To ensure reproducibility the text-mining process was done by programs applying perl-scripts and shell-scripts. For instance, regarding human terms,

a total of 8004 gene ontology terms were considered exactly defined and uniquely mapped to processes (gene ontology 2011, version 1,7). After iterative refinement 7609 terms or 95% of these 8004 unique gene ontology terms could be incorporated and mapped into the new non-overlapping classification of GO-Synthetic with 7 modules and 33 sub-modules.

After the last refinement-step a manual curation fixed any remaining inconsistencies. This manual step provides an additional check and security aspect to achieve a good quality during the mapping. Furthermore, this improves the added value of the classification and establishes it independent from other classifications and ontologies.

To have an additional validation an additional mapping between COG (Clusters of orthologous groups; Tatusov et al., 2003) and modules was done. This mapping was integrated into the classification including links to data and proteins as described in materials and methods.

We show here the mapping of the well annotated COGs to the top level of the module classification, however, also the next level (sub-processes) was considered in our analysis as well as all COGs and eukaryotic clusters of orthologous groups (KOGs).

After the last refinement-step a manual curation fixed any remaining inconsistencies. This manual step provides an additional check and security aspect to achieve a good quality during the mapping. Furthermore, this improves the added value of the classification and establishes it independent from other classifications and ontologies.

To have an additional validation an additional mapping between COG (Clusters of orthologous groups; Tatusov et al., 2003) and modules was done. This mapping was integrated into the classification including links to data and proteins as described in materials and methods.

We show here the mapping of the well annotated COGs to the top level of the module classification, however, also the next level (sub-processes) was considered in our analysis as well as all COGs and eukaryotic clusters of orthologous groups (KOGs).

## S2-Table 4. Modules – COG Mapping

| ***Categ.*** | ***cog*** | ***cog_name*** | ***module*** |
| --- | --- | --- | --- |
| [E] | COG1921 | Selenocysteine synthase [seryl-tRNASer selenium transferase] | Transport_container |
| [T] | COG0664 | cAMP-binding proteins - catabolite gene activator and regulatory subunit of cAMP-dependent protein kinases | Activation |
| [T] | COG3629 | DNA-binding transcriptional activator of the SARP family | Activation |
| [DKL] | COG5220 | Cdk activating kinase (CAK)/RNA polymerase II transcription initiation/nucleotide excision repair factor TFIIH, subunit TFB3 | Activation |
| [KL] | COG5242 | RNA polymerase II transcription initiation/nucleotide excision repair factor TFIIH, subunit TFB4 | Activation |
| [DKL] | COG5333 | Cdk activating kinase (CAK)/RNA polymerase II transcription initiation/nucleotide excision repair factor TFIIH/TFIIK, cyclin H subunit | Activation |
| [O] | COG5580 | Activator of HSP90 ATPase | Activation |
| [KL] | COG2901 | Factor for inversion stimulation Fis, transcriptional activator | Activation |
| [LD] | COG5067 | Protein kinase essential for the initiation of DNA replication | Activation |
| [KL] | COG5151 | RNA polymerase II transcription initiation/nucleotide excision repair factor TFIIH, subunit SSL1 | Activation |
| [K] | COG5179 | Transcription initiation factor TFIID, subunit TAF1 | Activation |
| [K] | COG5251 | Transcription initiation factor TFIID, subunit TAF11 | Activation |
| [L] | COG5527 | Protein involved in initiation of plasmid replication | Activation |
| [T] | COG3275 | Putative regulator of cell autolysis | Activation |
| [K] | COG5033 | Transcription initiation factor IIF, auxiliary subunit | Activation |
| [K] | COG5118 | Transcription initiation factor TFIIIB, Bdp1 subunit | Activation |
| [K] | COG5123 | Transcription initiation factor IIA, gamma subunit | Activation |
| [U] | COG5171 | Ran GTPase-activating protein (Ran-binding protein) | Activation |
| [L] | COG5534 | Plasmid replication initiator protein | Activation |
| [L] | COG5655 | Plasmid rolling circle replication initiator protein and truncated derivatives | Activation |
| [J] | COG0361 | Translation initiation factor 1 (IF-1) | Activation |
| [K] | COG4578 | Glucitol operon activator | Activation |
| [K] | COG5248 | Transcription initiation factor TFIID, subunit TAF13 | Activation |
| [O] | COG0602 | Organic radical activating enzymes | Activation |
| [K] | COG3617 | Prophage antirepressor | Activation |
| [K] | COG5149 | Transcription initiation factor IIA, large chain | Activation |
| [J] | COG5053 | Translation initiation factor 4E (eIF-4E) | Activation |
| [K] | COG5090 | Transcription initiation factor IIF, small subunit (RAP30) | Activation |
| [J] | COG5257 | Translation initiation factor 2, gamma subunit (eIF-2gamma; GTPase) | Activation |
| [P] | COG4548 | Nitric oxide reductase activation protein | Activation |
| [R] | COG5210 | GTPase-activating protein | Activation |
| [DT] | COG5057 | Phosphotyrosyl phosphatase activator | Activation |
| [J] | COG0050 | GTPases - translation elongation factors | Adjuvant |
| [O] | COG0071 | Molecular chaperone (small heat shock protein) | Adjuvant |
| [O] | COG0109 | Polyprenyltransferase (cytochrome oxidase assembly factor) | Adjuvant |
| [J] | COG0231 | Translation elongation factor P (EF-P)/translation initiation factor 5A (eIF-5A) | Adjuvant |
| [L] | COG0507 | ATP-dependent exoDNAse (exonuclease V), alpha subunit - helicase superfamily I member | Adjuvant |
| [G] | COG0580 | Glycerol uptake facilitator and related permeases (Major Intrinsic Protein Family) | Adjuvant |
| [KT] | COG1221 | Transcriptional regulators containing an AAA-type ATPase domain and a DNA-binding domain | Adjuvant |
| [O] | COG1281 | Disulfide bond chaperones of the HSP33 family | Adjuvant |
| [O] | COG1370 | Prefoldin, molecular chaperone implicated in de novo protein folding, alpha subunit | Adjuvant |
| [K] | COG1405 | Transcription initiation factor TFIIIB, Brf1 subunit/Transcription initiation factor TFIIB | Adjuvant |
| [O] | COG2377 | Predicted molecular chaperone distantly related to HSP70-fold metalloproteases | Adjuvant |
| [R] | COG3552 | Protein containing von Willebrand factor type A (vWA) domain | Adjuvant |
| [KL] | COG5144 | RNA polymerase II transcription initiation/nucleotide excision repair factor TFIIH, subunit TFB2 | Adjuvant |
| [K] | COG5162 | Transcription initiation factor TFIID, subunit TAF10 (also component of histone acetyltransferase SAGA) | Adjuvant |
| [K] | COG5296 | Transcription factor involved in TATA site selection and in elongation by RNA polymerase II | Adjuvant |
| [O] | COG5387 | Chaperone required for the assembly of the mitochondrial F1-ATPase | Adjuvant |
| [T] | COG5422 | RhoGEF, Guanine nucleotide exchange factor for Rho/Rac/Cdc42-like GTPases | Adjuvant |
| [J] | COG0009 | Putative translation factor (SUA5) | Adjuvant |
| [J] | COG0182 | Predicted translation initiation factor 2B subunit, eIF-2B alpha/beta/delta family | Adjuvant |
| [J] | COG0216 | Protein chain release factor A | Adjuvant |
| [D] | COG0239 | Integral membrane protein possibly involved in chromosome condensation | Adjuvant |
| [J] | COG0290 | Translation initiation factor 3 (IF-3) | Adjuvant |
| [L] | COG0592 | DNA polymerase sliding clamp subunit (PCNA homolog) | Adjuvant |
| [K] | COG1813 | Predicted transcription factor, homolog of eukaryotic MBF1 | Adjuvant |
| [NUO] | COG3418 | Flagellar biosynthesis/type III secretory pathway chaperone | Adjuvant |
| [R] | COG4588 | Accessory colonization factor AcfC, contains ABC-type periplasmic domain | Adjuvant |
| [A] | COG5107 | Pre-mRNA 3'-end processing (cleavage and polyadenylation) factor | Adjuvant |
| [KAD] | COG5147 | Myb superfamily proteins, including transcription factors and mRNA splicing factors | Adjuvant |
| [K] | COG5174 | Transcription initiation factor IIE, beta subunit | Adjuvant |
| [K] | COG5576 | Homeodomain-containing transcription factor | Adjuvant |
| [O] | COG0326 | Molecular chaperone, HSP90 family | Adjuvant |
| [K] | COG1308 | Transcription factor homologous to NACalpha-BTF3 | Adjuvant |
| [K] | COG1675 | Transcription initiation factor IIE, alpha subunit | Adjuvant |
| [J] | COG2890 | Methylase of polypeptide chain release factors | Adjuvant |
| [O] | COG3175 | Cytochrome oxidase assembly factor | Adjuvant |
| [R] | COG3331 | Penicillin-binding protein-related factor A, putative recombinase | Adjuvant |
| [BK] | COG5076 | Transcription factor involved in chromatin remodeling, contains bromodomain | Adjuvant |
| [K] | COG5094 | Transcription initiation factor TFIID, subunit TAF9 (also component of histone acetyltransferase SAGA) | Adjuvant |
| [K] | COG5095 | Transcription initiation factor TFIID, subunit TAF6 (also component of histone acetyltransferase SAGA) | Adjuvant |
| [KLB] | COG5165 | Nucleosome-binding factor SPN, POB3 subunit | Adjuvant |
| [J] | COG5256 | Translation elongation factor EF-1alpha (GTPase) | Adjuvant |
| [R] | COG5271 | AAA ATPase containing von Willebrand factor type A (vWA) domain | Adjuvant |
| [J] | COG0023 | Translation initiation factor 1 (eIF-1/SUI1) and related proteins | Adjuvant |
| [O] | COG0068 | Hydrogenase maturation factor | Adjuvant |
| [O] | COG0484 | DnaJ-class molecular chaperone with C-terminal Zn finger domain | Adjuvant |
| [O] | COG0542 | ATPases with chaperone activity, ATP-binding subunit | Adjuvant |
| [K] | COG0781 | Transcription termination factor | Adjuvant |
| [K] | COG0782 | Transcription elongation factor | Adjuvant |
| [J] | COG1093 | Translation initiation factor 2 alpha subunit (eIF-2alpha) | Adjuvant |
| [O] | COG1214 | Inactive homolog of metal-dependent proteases, putative molecular chaperone | Adjuvant |
| [J] | COG1503 | Peptide chain release factor 1 (eRF1) | Adjuvant |
| [O] | COG1975 | Xanthine and CO dehydrogenases maturation factor, XdhC/CoxF family | Adjuvant |
| [J] | COG3276 | Selenocysteine-specific translation elongation factor | Adjuvant |
| [K] | COG4008 | Predicted metal-binding transcription factor | Adjuvant |
| [K] | COG5025 | Transcription factor of the Forkhead/HNF3 family | Adjuvant |
| [A] | COG5623 | Pre-mRNA cleavage and polyadenylation factor IA/II complex, subunit CLP1 | Adjuvant |
| [R] | COG0073 | EMAP domain | Adjuvant |
| [K] | COG0195 | Transcription elongation factor | Adjuvant |
| [R] | COG0218 | Predicted GTPase | Adjuvant |
| [O] | COG0309 | Hydrogenase maturation factor | Adjuvant |
| [O] | COG0576 | Molecular chaperone GrpE (heat shock protein) | Adjuvant |
| [D] | COG0851 | Septum formation topological specificity factor | Adjuvant |
| [J] | COG1601 | Translation initiation factor 2 beta subunit (eIF-2beta)/eIF-5 N-terminal domain | Adjuvant |
| [O] | COG1730 | Predicted prefoldin, molecular chaperone implicated in de novo protein folding | Adjuvant |
| [O] | COG1973 | Hydrogenase maturation factor | Adjuvant |
| [J] | COG3130 | Ribosome modulation factor | Adjuvant |
| [D] | COG3640 | CO dehydrogenase maturation factor | Adjuvant |
| [T] | COG3806 | Anti-sigma factor | Adjuvant |
| [E] | COG4820 | Ethanolamine utilization protein, possible chaperonin | Adjuvant |
| [K] | COG4903 | Genetic competence transcription factor | Adjuvant |
| [J] | COG0264 | Translation elongation factor Ts | Adjuvant |
| [O] | COG0409 | Hydrogenase maturation factor | Adjuvant |
| [O] | COG0443 | Molecular chaperone | Adjuvant |
| [O] | COG0459 | Chaperonin GroEL (HSP60 family) | Adjuvant |
| [O] | COG0606 | Predicted ATPase with chaperone activity | Adjuvant |
| [C] | COG0680 | Ni,Fe-hydrogenase maturation factor | Adjuvant |
| [K] | COG1158 | Transcription termination factor | Adjuvant |
| [K] | COG1309 | Transcriptional regulator | Adjuvant |
| [NUO] | COG1516 | Flagellin-specific chaperone FliS | Adjuvant |
| [K] | COG1594 | DNA-directed RNA polymerase, subunit M/Transcription elongation factor TFIIS | Adjuvant |
| [K] | COG1595 | DNA-directed RNA polymerase specialized sigma subunit, sigma24 homolog | Adjuvant |
| [J] | COG2092 | Translation elongation factor EF-1beta | Adjuvant |
| [NUO] | COG2882 | Flagellar biosynthesis chaperone | Adjuvant |
| [NU] | COG3121 | P pilus assembly protein, chaperone PapD | Adjuvant |
| [KB] | COG5137 | Histone chaperone involved in gene silencing | Adjuvant |
| [K] | COG5414 | TATA-binding protein-associated factor | Adjuvant |
| [N] | COG5622 | Protein required for attachment to host cells | Adjuvant |
| [J] | COG0233 | Ribosome recycling factor | Adjuvant |
| [O] | COG0234 | Co-chaperonin GroES (HSP10) | Adjuvant |
| [J] | COG0480 | Translation elongation factors (GTPases) | Adjuvant |
| [O] | COG0501 | Zn-dependent protease with chaperone function | Adjuvant |
| [K] | COG0583 | Transcriptional regulator | Adjuvant |
| [J] | COG1184 | Translation initiation factor 2B subunit, eIF-2B alpha/beta/delta family | Adjuvant |
| [O] | COG1222 | ATP-dependent 26S proteasome regulatory subunit | Adjuvant |
| [O] | COG1382 | Prefoldin, chaperonin cofactor | Adjuvant |
| [K] | COG1414 | Transcriptional regulator | Adjuvant |
| [J] | COG1976 | Translation initiation factor 6 (eIF-6) | Adjuvant |
| [H] | COG1977 | Molybdopterin converting factor, small subunit | Adjuvant |
| [P] | COG2608 | Copper chaperone | Adjuvant |
| [UD] | COG5079 | Nuclear protein export factor | Adjuvant |
| [K] | COG5204 | Transcription elongation factor SPT4 | Adjuvant |
| [K] | COG5208 | CCAAT-binding factor, subunit C | Adjuvant |
| [K] | COG5221 | Dopey and related predicted leucine zipper transcription factors | Adjuvant |
| [O] | COG1138 | Cytochrome c biogenesis factor | Adjuvant |
| [K] | COG5157 | RNA polymerase II assessory factor | Adjuvant |
| [OZ] | COG5234 | Beta-tubulin folding cofactor D | Adjuvant |
| [O] | COG5380 | Lipase chaperone | Adjuvant |
| [K] | COG5641 | GATA Zn-finger-containing transcription factor | Adjuvant |
| [J] | COG0532 | Translation initiation factor 2 (IF-2; GTPase)""" | Activation |
| [J] | COG1186 | Protein chain release factor B | Adjuvant |
| [K] | COG2183 | Transcriptional accessory protein | Adjuvant |
| [O] | COG2214 | DnaJ-class molecular chaperone | Adjuvant |
| [K] | COG5164 | Transcription elongation factor | Adjuvant |
| [JO] | COG5269 | Ribosome-associated chaperone zuotin | Adjuvant |
| [K] | COG0819 | Putative transcription activator | Adjuvant |
| [K] | COG1316 | Transcriptional regulator | Adjuvant |
| [O] | COG4235 | Cytochrome c biogenesis factor | Adjuvant |
| [K] | COG5190 | TFIIF-interacting CTD phosphatases, including NLI-interacting factor | Adjuvant |
| [K] | COG5224 | CCAAT-binding factor, subunit B | Adjuvant |
| [M] | COG1664 | Integral membrane protein CcmA involved in cell shape determination | Barrier |
| [S] | COG2431 | Predicted membrane protein | Barrier |
| [M] | COG3017 | Outer membrane lipoprotein involved in outer membrane biogenesis | Barrier |
| [M] | COG3064 | Membrane protein involved in colicin uptake | Barrier |
| [V] | COG3725 | Membrane protein required for beta-lactamase induction | Barrier |
| [M] | COG4932 | Predicted outer membrane protein | Barrier |
| [R] | COG4956 | Integral membrane protein (PIN domain superfamily) | Barrier |
| [S] | COG5436 | Predicted integral membrane protein | Barrier |
| [S] | COG5446 | Predicted integral membrane protein | Barrier |
| [P] | COG5456 | Predicted integral membrane protein linked to a cation pump | Barrier |
| [S] | COG5472 | Predicted small integral membrane protein | Barrier |
| [S] | COG5477 | Predicted small integral membrane protein | Barrier |
| [S] | COG5478 | Predicted small integral membrane protein | Barrier |
| [N] | COG5571 | Autotransporter protein or domain, integral membrane beta-barrel involved in protein secretion | Barrier |
| [S] | COG5578 | Predicted integral membrane protein | Barrier |
| [P] | COG0310 | ABC-type Co2+ transport system, permease component | Barrier |
| [EP] | COG0444 | ABC-type dipeptide/oligopeptide/nickel transport system, ATPase component | Barrier |
| [P] | COG1629 | Outer membrane receptor proteins, mostly Fe transport | Barrier |
| [T] | COG1966 | Carbon starvation protein, predicted membrane protein | Barrier |
| [M] | COG2885 | Outer membrane protein and related peptidoglycan-associated (lipo)proteins | Barrier |
| [S] | COG3371 | Predicted membrane protein | Barrier |
| [S] | COG3374 | Predicted membrane protein | Barrier |
| [S] | COG4035 | Predicted membrane protein | Barrier |
| [S] | COG4036 | Predicted membrane protein | Barrier |
| [S] | COG4037 | Predicted membrane protein | Barrier |
| [S] | COG4038 | Predicted membrane protein | Barrier |
| [S] | COG4039 | Predicted membrane protein | Barrier |
| [S] | COG4040 | Predicted membrane protein | Barrier |
| [S] | COG4041 | Predicted membrane protein | Barrier |
| [S] | COG4042 | Predicted membrane protein | Barrier |
| [S] | COG4325 | Predicted membrane protein | Barrier |
| [S] | COG4327 | Predicted membrane protein | Barrier |
| [S] | COG4329 | Predicted membrane protein | Barrier |
| [S] | COG4330 | Predicted membrane protein | Barrier |
| [S] | COG4331 | Predicted membrane protein | Barrier |
| [O] | COG4846 | Membrane protein involved in cytochrome C biogenesis | Barrier |
| [S] | COG4920 | Predicted membrane protein | Barrier |
| [S] | COG5438 | Predicted multitransmembrane protein | Barrier |
| [S] | COG5463 | Predicted integral membrane protein | Barrier |
| [S] | COG5473 | Predicted integral membrane protein | Barrier |
| [S] | COG0344 | Predicted membrane protein | Barrier |
| [R] | COG1480 | Predicted membrane-associated HD superfamily hydrolase | Barrier |
| [M] | COG2834 | Outer membrane lipoprotein-sorting protein | Barrier |
| [M] | COG3065 | Starvation-inducible outer membrane lipoprotein | Barrier |
| [M] | COG3137 | Putative salt-induced outer membrane protein | Barrier |
| [M] | COG3248 | Nucleoside-binding outer membrane protein | Barrier |
| [S] | COG3671 | Predicted membrane protein | Barrier |
| [S] | COG3748 | Predicted membrane protein | Barrier |
| [S] | COG3752 | Predicted membrane protein | Barrier |
| [S] | COG3759 | Predicted membrane protein | Barrier |
| [S] | COG3762 | Predicted membrane protein | Barrier |
| [S] | COG3766 | Predicted membrane protein | Barrier |
| [S] | COG3768 | Predicted membrane protein | Barrier |
| [S] | COG4720 | Predicted membrane protein | Barrier |
| [S] | COG4721 | Predicted membrane protein | Barrier |
| [S] | COG4732 | Predicted membrane protein | Barrier |
| [C] | COG4781 | Membrane domain of membrane-anchored glycerophosphoryl diester phosphodiesterase | Barrier |
| [S] | COG4890 | Predicted outer membrane lipoprotein | Barrier |
| [M] | COG4953 | Membrane carboxypeptidase/penicillin-binding protein PbpC | Barrier |
| [S] | COG5500 | Predicted integral membrane protein | Barrier |
| [S] | COG5505 | Predicted integral membrane protein | Barrier |
| [S] | COG5528 | Predicted integral membrane protein | Barrier |
| [S] | COG5530 | Predicted integral membrane protein | Barrier |
| [S] | COG5542 | Predicted integral membrane protein | Barrier |
| [S] | COG5605 | Predicted small integral membrane protein | Barrier |
| [S] | COG5658 | Predicted integral membrane protein | Barrier |
| [S] | COG5660 | Predicted integral membrane protein | Barrier |
| [P] | COG0168 | Trk-type K+ transport systems, membrane components | Barrier |
| [M] | COG0791 | Cell wall-associated hydrolases (invasion-associated proteins) | Barrier |
| [M] | COG1696 | Predicted membrane protein involved in D-alanine export | Barrier |
| [M] | COG2821 | Membrane-bound lytic murein transglycosylase | Barrier |
| [S] | COG3162 | Predicted membrane protein | Barrier |
| [S] | COG3164 | Predicted membrane protein | Barrier |
| [S] | COG3174 | Predicted membrane protein | Barrier |
| [T] | COG3448 | CBS-domain-containing membrane protein | Barrier |
| [S] | COG3503 | Predicted membrane protein | Barrier |
| [M] | COG3659 | Carbohydrate-selective porin | Barrier |
| [M] | COG3713 | Outer membrane protein V | Barrier |
| [P] | COG3746 | Phosphate-selective porin | Barrier |
| [S] | COG3815 | Predicted membrane protein | Barrier |
| [S] | COG3817 | Predicted membrane protein | Barrier |
| [S] | COG3819 | Predicted membrane protein | Barrier |
| [S] | COG3918 | Predicted membrane protein | Barrier |
| [S] | COG3924 | Predicted membrane protein | Barrier |
| [S] | COG4292 | Predicted membrane protein | Barrier |
| [O] | COG4346 | Predicted membrane-bound dolichyl-phosphate-mannose-protein mannosyltransferase | Barrier |
| [S] | COG4392 | Predicted membrane protein | Barrier |
| [S] | COG4393 | Predicted membrane protein | Barrier |
| [S] | COG4420 | Predicted membrane protein | Barrier |
| [S] | COG4425 | Predicted membrane protein | Barrier |
| [M] | COG4571 | Outer membrane protease | Barrier |
| [S] | COG4682 | Predicted membrane protein | Barrier |
| [S] | COG4684 | Predicted membrane protein | Barrier |
| [G] | COG4945 | Membrane-anchored protein predicted to be involved in regulation of amylopullulanase | Barrier |
| [R] | COG5415 | Predicted integral membrane metal-binding protein | Barrier |
| [S] | COG5486 | Predicted metal-binding integral membrane protein | Barrier |
| [P] | COG0530 | Ca2+/Na+ antiporter | Barrier |
| [S] | COG1297 | Predicted membrane protein | Barrier |
| [S] | COG1822 | Predicted archaeal membrane protein | Barrier |
| [S] | COG1967 | Predicted membrane protein | Barrier |
| [M] | COG1970 | Large-conductance mechanosensitive channel | Barrier |
| [S] | COG1971 | Predicted membrane protein | Barrier |
| [S] | COG2034 | Predicted membrane protein | Barrier |
| [S] | COG2035 | Predicted membrane protein | Barrier |
| [S] | COG2119 | Predicted membrane protein | Barrier |
| [M] | COG2825 | Outer membrane protein | Barrier |
| [M] | COG3133 | Outer membrane lipoprotein | Barrier |
| [S] | COG3212 | Predicted membrane protein | Barrier |
| [S] | COG3223 | Predicted membrane protein | Barrier |
| [S] | COG3556 | Predicted membrane protein | Barrier |
| [L] | COG3611 | Replication initiation/membrane attachment protein | Barrier |
| [S] | COG3652 | Predicted outer membrane protein | Barrier |
| [S] | COG3714 | Predicted membrane protein | Barrier |
| [S] | COG3771 | Predicted membrane protein | Barrier |
| [S] | COG3776 | Predicted membrane protein | Barrier |
| [S] | COG3781 | Predicted membrane protein | Barrier |
| [S] | COG3790 | Predicted membrane protein | Barrier |
| [S] | COG3859 | Predicted membrane protein | Barrier |
| [S] | COG4025 | Predicted membrane protein | Barrier |
| [S] | COG4280 | Predicted membrane protein | Barrier |
| [S] | COG4291 | Predicted membrane protein | Barrier |
| [S] | COG4347 | Predicted membrane protein | Barrier |
| [S] | COG4478 | Predicted membrane protein | Barrier |
| [S] | COG4708 | Predicted membrane protein | Barrier |
| [S] | COG4709 | Predicted membrane protein | Barrier |
| [S] | COG4711 | Predicted membrane protein | Barrier |
| [S] | COG4713 | Predicted membrane protein | Barrier |
| [S] | COG5254 | Predicted membrane protein | Barrier |
| [S] | COG5480 | Predicted integral membrane protein | Barrier |
| [S] | COG5487 | Small integral membrane protein | Barrier |
| [S] | COG5521 | Predicted integral membrane protein | Barrier |
| [S] | COG5522 | Predicted integral membrane protein | Barrier |
| [S] | COG5523 | Predicted integral membrane protein | Barrier |
| [S] | COG5563 | Predicted integral membrane proteins containing uncharacterized repeats | Barrier |
| [S] | COG5637 | Predicted integral membrane protein | Barrier |
| [S] | COG1422 | Predicted membrane protein | Barrier |
| [S] | COG1808 | Predicted membrane protein | Barrier |
| [S] | COG2364 | Predicted membrane protein | Barrier |
| [S] | COG2510 | Predicted membrane protein | Barrier |
| [M] | COG3047 | Outer membrane protein W | Barrier |
| [M] | COG3203 | Outer membrane protein (porin) | Barrier |
| [P] | COG3301 | Formate-dependent nitrite reductase, membrane component | Barrier |
| [S] | COG3326 | Predicted membrane protein | Barrier |
| [S] | COG3336 | Predicted membrane protein | Barrier |
| [S] | COG3462 | Predicted membrane protein | Barrier |
| [S] | COG3463 | Predicted membrane protein | Barrier |
| [S] | COG3548 | Predicted integral membrane protein | Barrier |
| [S] | COG3647 | Predicted membrane protein | Barrier |
| [S] | COG3650 | Predicted membrane protein | Barrier |
| [S] | COG4125 | Predicted membrane protein | Barrier |
| [S] | COG4267 | Predicted membrane protein | Barrier |
| [S] | COG4269 | Predicted membrane protein | Barrier |
| [S] | COG4270 | Predicted membrane protein | Barrier |
| [S] | COG4272 | Predicted membrane protein | Barrier |
| [S] | COG4371 | Predicted membrane protein | Barrier |
| [S] | COG4377 | Predicted membrane protein | Barrier |
| [S] | COG4655 | Predicted membrane protein | Barrier |
| [S] | COG4803 | Predicted membrane protein | Barrier |
| [S] | COG4852 | Predicted membrane protein | Barrier |
| [S] | COG4905 | Predicted membrane protein | Barrier |
| [S] | COG4906 | Predicted membrane protein | Barrier |
| [S] | COG4907 | Predicted membrane protein | Barrier |
| [U] | COG5100 | Nuclear pore protein | Barrier |
| [S] | COG5455 | Predicted integral membrane protein | Barrier |
| [S] | COG5488 | Integral membrane protein | Barrier |
| [S] | COG5546 | Small integral membrane protein | Barrier |
| [S] | COG5547 | Small integral membrane protein | Barrier |
| [S] | COG5548 | Small integral membrane protein | Barrier |
| [P] | COG0025 | NhaP-type Na+/H+ and K+/H+ antiporters | Barrier |
| [P] | COG0038 | Chloride channel protein EriC | Barrier |
| [ER] | COG0591 | Na+/proline symporter | Barrier |
| [D] | COG0772 | Bacterial cell division membrane protein | Barrier |
| [S] | COG1836 | Predicted membrane protein | Barrier |
| [S] | COG1950 | Predicted membrane protein | Barrier |
| [S] | COG2324 | Predicted membrane protein | Barrier |
| [S] | COG2339 | Predicted membrane protein | Barrier |
| [S] | COG2707 | Predicted membrane protein | Barrier |
| [S] | COG2717 | Predicted membrane protein | Barrier |
| [S] | COG2733 | Predicted membrane protein | Barrier |
| [S] | COG3205 | Predicted membrane protein | Barrier |
| [S] | COG3686 | Predicted membrane protein | Barrier |
| [S] | COG3821 | Predicted membrane protein | Barrier |
| [S] | COG4078 | Predicted membrane protein | Barrier |
| [S] | COG4769 | Predicted membrane protein | Barrier |
| [S] | COG4811 | Predicted membrane protein | Barrier |
| [S] | COG4818 | Predicted membrane protein | Barrier |
| [S] | COG4828 | Predicted membrane protein | Barrier |
| [S] | COG4836 | Predicted membrane protein | Barrier |
| [S] | COG5393 | Predicted membrane protein | Barrier |
| [S] | COG5395 | Predicted membrane protein | Barrier |
| [M] | COG0481 | Membrane GTPase LepA | Barrier |
| [R] | COG0670 | Integral membrane protein, interacts with FtsH | Barrier |
| [S] | COG0762 | Predicted integral membrane protein | Barrier |
| [MU] | COG1538 | Outer membrane protein | Barrier |
| [S] | COG1584 | Predicted membrane protein | Barrier |
| [S] | COG1714 | Predicted membrane protein/domain | Barrier |
| [M] | COG2247 | Putative cell wall-binding domain | Barrier |
| [S] | COG3152 | Predicted membrane protein | Barrier |
| [S] | COG3235 | Predicted membrane protein | Barrier |
| [M] | COG3264 | Small-conductance mechanosensitive channel | Barrier |
| [S] | COG3304 | Predicted membrane protein | Barrier |
| [S] | COG3305 | Predicted membrane protein | Barrier |
| [S] | COG3308 | Predicted membrane protein | Barrier |
| [S] | COG3601 | Predicted membrane protein | Barrier |
| [S] | COG3952 | Predicted membrane protein | Barrier |
| [S] | COG4129 | Predicted membrane protein | Barrier |
| [S] | COG4323 | Predicted membrane protein | Barrier |
| [S] | COG4872 | Predicted membrane protein | Barrier |
| [S] | COG4881 | Predicted membrane protein | Barrier |
| [U] | COG5052 | Protein involved in membrane traffic | Barrier |
| [S] | COG5081 | Predicted membrane protein | Barrier |
| [S] | COG5202 | Predicted membrane protein | Barrier |
| [U] | COG5233 | Peripheral Golgi membrane protein | Barrier |
| [S] | COG5572 | Predicted integral membrane protein | Barrier |
| [S] | COG5650 | Predicted integral membrane protein | Barrier |
| [S] | COG5652 | Predicted integral membrane protein | Barrier |
| [P] | COG0370 | Fe2+ transport system protein B | Barrier |
| [S] | COG0392 | Predicted integral membrane protein | Barrier |
| [M] | COG0750 | Predicted membrane-associated Zn-dependent proteases 1 | Barrier |
| [S] | COG1470 | Predicted membrane protein | Barrier |
| [S] | COG1981 | Predicted membrane protein | Barrier |
| [S] | COG2149 | Predicted membrane protein | Barrier |
| [R] | COG2194 | Predicted membrane-associated, metal-dependent hydrolase | Barrier |
| [S] | COG2237 | Predicted membrane protein | Barrier |
| [S] | COG2245 | Predicted membrane protein | Barrier |
| [S] | COG2246 | Predicted membrane protein | Barrier |
| [S] | COG2259 | Predicted membrane protein | Barrier |
| [S] | COG2261 | Predicted membrane protein | Barrier |
| [S] | COG2426 | Predicted membrane protein | Barrier |
| [S] | COG2855 | Predicted membrane protein | Barrier |
| [S] | COG3356 | Predicted membrane protein | Barrier |
| [S] | COG3428 | Predicted membrane protein | Barrier |
| [S] | COG3431 | Predicted membrane protein | Barrier |
| [S] | COG3689 | Predicted membrane protein | Barrier |
| [S] | COG4083 | Predicted membrane protein | Barrier |
| [S] | COG4089 | Predicted membrane protein | Barrier |
| [S] | COG4094 | Predicted membrane protein | Barrier |
| [R] | COG4194 | Predicted membrane protein | Barrier |
| [S] | COG4743 | Predicted membrane protein | Barrier |
| [S] | COG4758 | Predicted membrane protein | Barrier |
| [S] | COG4760 | Predicted membrane protein | Barrier |
| [S] | COG4763 | Predicted membrane protein | Barrier |
| [S] | COG4854 | Predicted membrane protein | Barrier |
| [S] | COG4939 | Major membrane immunogen, membrane-anchored lipoprotein | Barrier |
| [S] | COG5237 | Predicted membrane protein | Barrier |
| [S] | COG5373 | Predicted membrane protein | Barrier |
| [S] | COG5612 | Predicted integral membrane protein | Barrier |
| [S] | COG5615 | Predicted integral membrane protein | Barrier |
| [S] | COG5616 | Predicted integral membrane protein | Barrier |
| [S] | COG5617 | Predicted integral membrane protein | Barrier |
| [M] | COG0729 | Outer membrane protein | Barrier |
| [C] | COG4237 | Hydrogenase 4 membrane component (E) | Barrier |
| [S] | COG4485 | Predicted membrane protein | Barrier |
| [S] | COG4499 | Predicted membrane protein | Barrier |
| [S] | COG4640 | Predicted membrane protein | Barrier |
| [S] | COG4648 | Predicted membrane protein | Barrier |
| [S] | COG4984 | Predicted membrane protein | Barrier |
| [P] | COG0387 | Ca2+/H+ antiporter | Barrier |
| [M] | COG0668 | Small-conductance mechanosensitive channel | Barrier |
| [S] | COG1784 | Predicted membrane protein | Barrier |
| [S] | COG4550 | Predicted membrane protein | Barrier |
| [S] | COG2311 | Predicted membrane protein | Barrier |
| [S] | COG2314 | Predicted membrane protein | Barrier |
| [S] | COG2322 | Predicted membrane protein | Barrier |
| [S] | COG2323 | Predicted membrane protein | Barrier |
| [S] | COG4241 | Predicted membrane protein | Barrier |
| [S] | COG4539 | Predicted membrane protein | Barrier |
| [S] | COG4541 | Predicted membrane protein | Barrier |
| [M] | COG5009 | Membrane carboxypeptidase/penicillin-binding protein | Barrier |
| [S] | COG5085 | Predicted membrane protein | Barrier |
| [S] | COG5291 | Predicted membrane protein | Barrier |
| [S] | COG5305 | Predicted membrane protein | Barrier |
| [S] | COG5346 | Predicted membrane protein | Barrier |
| [S] | COG4243 | Predicted membrane protein | Barrier |
| [S] | COG4244 | Predicted membrane protein | Barrier |
| [S] | COG5197 | Predicted membrane protein | Barrier |
| [S] | COG2860 | Predicted membrane protein | Barrier |
| [S] | COG2862 | Predicted membrane protein | Barrier |
| [E] | COG0076 | Glutamate decarboxylase and related PLP-dependent proteins | Branching |
| [HE] | COG0111 | Phosphoglycerate dehydrogenase and related dehydrogenases | Branching |
| [BQ] | COG0123 | Deacetylases, including yeast histone deacetylase and acetoin utilization protein | Branching |
| [O] | COG0229 | Conserved domain frequently associated with peptide methionine sulfoxide reductase | Branching |
| [O] | COG0265 | Trypsin-like serine proteases, typically periplasmic, contain C-terminal PDZ domain | Branching |
| [R] | COG0300 | Short-chain dehydrogenases of various substrate specificities | Branching |
| [L] | COG0322 | Nuclease subunit of the excinuclease complex | Branching |
| [E] | COG0403 | Glycine cleavage system protein P (pyridoxal-binding), N-terminal domain | Branching |
| [CP] | COG0651 | Formate hydrogenlyase subunit 3/Multisubunit Na+/H+ antiporter, MnhD subunit | Branching |
| [L] | COG0847 | DNA polymerase III, epsilon subunit and related 3'-5' exonucleases | Branching |
| [IQR] | COG1028 | Dehydrogenases with different specificities (related to short-chain alcohol dehydrogenases) | Branching |
| [E] | COG1296 | Predicted branched-chain amino acid permease (azaleucine resistance) | Branching |
| [R] | COG1752 | Predicted esterase of the alpha-beta hydrolase superfamily | Branching |
| [G] | COG2342 | Predicted extracellular endo alpha-1,4 polygalactosaminidase or related polysaccharide hydrolase | Branching |
| [L] | COG3298 | Predicted 3'-5' exonuclease related to the exonuclease domain of PolB | Branching |
| [T] | COG3480 | Predicted secreted protein containing a PDZ domain | Branching |
| [R] | COG5084 | Cleavage and polyadenylation specificity factor (CPSF) Clipper subunit and related makorin family Zn-finger proteins | Branching |
| [D] | COG5657 | CAS/CSE protein involved in chromosome segregation | Branching |
| [C] | COG0022 | Pyruvate/2-oxoglutarate dehydrogenase complex, dehydrogenase (E1) component, eukaryotic type, beta subunit | Branching |
| [C] | COG0508 | Pyruvate/2-oxoglutarate dehydrogenase complex, dihydrolipoamide acyltransferase (E2) component, and related enzymes | Branching |
| [C] | COG0567 | 2-oxoglutarate dehydrogenase complex, dehydrogenase (E1) component, and related enzymes | Branching |
| [C] | COG1071 | Pyruvate/2-oxoglutarate dehydrogenase complex, dehydrogenase (E1) component, eukaryotic type, alpha subunit | Branching |
| [C] | COG1529 | Aerobic-type carbon monoxide dehydrogenase, large subunit CoxL/CutL homologs | Branching |
| [D] | COG1674 | DNA segregation ATPase FtsK/SpoIIIE and related proteins | Branching |
| [I] | COG1924 | Activator of 2-hydroxyglutaryl-CoA dehydratase (HSP70-class ATPase domain) | Branching |
| [NU] | COG2165 | Type II secretory pathway, pseudopilin PulG | Branching |
| [I] | COG3407 | Mevalonate pyrophosphate decarboxylase | Branching |
| [R] | COG3443 | Predicted periplasmic or secreted protein | Branching |
| [R] | COG4667 | Predicted esterase of the alpha-beta hydrolase superfamily | Branching |
| [F] | COG4741 | Predicted secreted endonuclease distantly related to archaeal Holliday junction resolvase | Branching |
| [R] | COG4927 | Predicted choloylglycine hydrolase | Branching |
| [A] | COG5176 | Splicing factor (branch point binding protein) | Branching |
| [S] | COG5437 | Predicted secreted protein | Branching |
| [E] | COG0002 | Acetylglutamate semialdehyde dehydrogenase | Branching |
| [E] | COG0014 | Gamma-glutamyl phosphate reductase | Branching |
| [G] | COG0057 | Glyceraldehyde-3-phosphate dehydrogenase/erythrose-4-phosphate dehydrogenase | Branching |
| [E] | COG0065 | 3-isopropylmalate dehydratase large subunit | Branching |
| [E] | COG0066 | 3-isopropylmalate dehydratase small subunit | Branching |
| [EQ] | COG0145 | N-methylhydantoinase A/acetone carboxylase, beta subunit | Branching |
| [EQ] | COG0146 | N-methylhydantoinase B/acetone carboxylase, alpha subunit | Branching |
| [P] | COG0155 | Sulfite reductase, beta subunit (hemoprotein) | Branching |
| [F] | COG0208 | Ribonucleotide reductase, beta subunit | Branching |
| [F] | COG0209 | Ribonucleotide reductase, alpha subunit | Branching |
| [C] | COG0243 | Anaerobic dehydrogenases, typically selenocysteine-containing | Branching |
| [L] | COG0258 | 5'-3' exonuclease (including N-terminal domain of PolI) | Branching |
| [E] | COG0404 | Glycine cleavage system T protein (aminomethyltransferase) | Branching |
| [R] | COG0561 | Predicted hydrolases of the HAD superfamily | Branching |
| [C] | COG0649 | NADH:ubiquinone oxidoreductase 49 kD subunit 7 | Branching |
| [HC] | COG0654 | 2-polyprenyl-6-methoxyphenol hydroxylase and related FAD-dependent oxidoreductases | Branching |
| [R] | COG0656 | Aldo/keto reductases, related to diketogulonate reductase | Branching |
| [C] | COG1018 | Flavodoxin reductases (ferredoxin-NADPH reductases) family 1 | Branching |
| [Q] | COG2015 | Alkyl sulfatase and related hydrolases | Branching |
| [R] | COG2179 | Predicted hydrolase of the HAD superfamily | Branching |
| [M] | COG2335 | Secreted and surface protein containing fasciclin-like repeats | Branching |
| [R] | COG3083 | Predicted hydrolase of alkaline phosphatase superfamily | Branching |
| [R] | COG4324 | Predicted aminopeptidase | Branching |
| [A] | COG5182 | Splicing factor 3b, subunit 2 | Branching |
| [R] | COG5322 | Predicted dehydrogenase | Branching |
| [J] | COG0144 | tRNA and rRNA cytosine-C5-methylases | Branching |
| [J] | COG0293 | 23S rRNA methylase | Branching |
| [G] | COG0296 | 1,4-alpha-glucan branching enzyme | Branching |
| [E] | COG0334 | Glutamate dehydrogenase/leucine dehydrogenase | Branching |
| [E] | COG0345 | Pyrroline-5-carboxylate reductase | Branching |
| [C] | COG0437 | Fe-S-cluster-containing hydrogenase components 1 | Branching |
| [F] | COG0516 | IMP dehydrogenase/GMP reductase | Branching |
| [R] | COG1380 | Putative effector of murein hydrolase LrgA | Branching |
| [E] | COG1687 | Predicted branched-chain amino acid permeases (azaleucine resistance) | Branching |
| [O] | COG2039 | Pyrrolidone-carboxylate peptidase (N-terminal pyroglutamyl peptidase) | Branching |
| [PR] | COG2146 | Ferredoxin subunits of nitrite reductase and ring-hydroxylating dioxygenases | Branching |
| [R] | COG2249 | Putative NADPH-quinone reductase (modulator of drug activity B) | Branching |
| [C] | COG2838 | Monomeric isocitrate dehydrogenase | Branching |
| [R] | COG2936 | Predicted acyl esterases | Branching |
| [Q] | COG3458 | Acetyl esterase (deacetylase) | Branching |
| [Q] | COG3805 | Aromatic ring-cleaving dioxygenase | Branching |
| [Q] | COG4569 | Acetaldehyde dehydrogenase (acetylating) | Branching |
| [G] | COG4724 | Endo-beta-N-acetylglucosaminidase D | Branching |
| [A] | COG5178 | U5 snRNP spliceosome subunit | Branching |
| [A] | COG5181 | U2 snRNP spliceosome subunit | Branching |
| [S] | COG5501 | Predicted secreted protein | Branching |
| [E] | COG0077 | Prephenate dehydratase | Branching |
| [EH] | COG0115 | Branched-chain amino acid aminotransferase/4-amino-4-deoxychorismate lyase | Branching |
| [Q] | COG0179 | 2-keto-4-pentenoate hydratase/2-oxohepta-3-ene-1,7-dioic acid hydratase (catechol pathway) | Branching |
| [H] | COG0212 | 5-formyltetrahydrofolate cyclo-ligase | Branching |
| [C] | COG0240 | Glycerol-3-phosphate dehydrogenase | Branching |
| [G] | COG0246 | Mannitol-1-phosphate/altronate dehydrogenases | Branching |
| [C] | COG0247 | Fe-S oxidoreductase | Branching |
| [E] | COG0289 | Dihydrodipicolinate reductase | Branching |
| [F] | COG0295 | Cytidine deaminase | Branching |
| [R] | COG0319 | Predicted metal-dependent hydrolase | Branching |
| [C] | COG0377 | NADH:ubiquinone oxidoreductase 20 kD subunit and related Fe-S oxidoreductases | Branching |
| [C] | COG0479 | Succinate dehydrogenase/fumarate reductase, Fe-S protein subunit | Branching |
| [C] | COG0650 | Formate hydrogenlyase subunit 4 | Branching |
| [E] | COG1003 | Glycine cleavage system protein P (pyridoxal-binding), C-terminal domain | Branching |
| [V] | COG1401 | GTPase subunit of restriction endonuclease | Branching |
| [C] | COG1454 | Alcohol dehydrogenase, class IV | Branching |
| [G] | COG1494 | Fructose-1,6-bisphosphatase/sedoheptulose 1,7-bisphosphatase and related proteins | Branching |
| [G] | COG1554 | Trehalose and maltose hydrolases (possible phosphorylases) | Branching |
| [F] | COG1864 | DNA/RNA endonuclease G, NUC1 | Branching |
| [L] | COG2254 | Predicted HD superfamily hydrolase, possibly a nuclease | Branching |
| [L] | COG2356 | Endonuclease I | Branching |
| [R] | COG2514 | Predicted ring-cleavage extradiol dioxygenase | Branching |
| [G] | COG3408 | Glycogen debranching enzyme | Branching |
| [R] | COG4341 | Predicted HD phosphohydrolase | Branching |
| [R] | COG4915 | 5-bromo-4-chloroindolyl phosphate hydrolysis protein | Branching |
| [H] | COG0043 | 3-polyprenyl-4-hydroxybenzoate decarboxylase and related decarboxylases | Branching |
| [F] | COG0044 | Dihydroorotase and related cyclic amidohydrolases | Branching |
| [L] | COG0116 | Predicted N6-adenine-specific DNA methylase | Branching |
| [F] | COG0151 | Phosphoribosylamine-glycine ligase | Branching |
| [J] | COG0193 | Peptidyl-tRNA hydrolase | Branching |
| [O] | COG0225 | Peptide methionine sulfoxide reductase | Branching |
| [FP] | COG0248 | Exopolyphosphatase | Branching |
| [L] | COG0420 | DNA repair exonuclease | Branching |
| [E] | COG0509 | Glycine cleavage system H protein (lipoate-binding) | Branching |
| [R] | COG0613 | Predicted metal-dependent phosphoesterases (PHP family) | Branching |
| [C] | COG0674 | Pyruvate:ferredoxin oxidoreductase and related 2-oxoacid:ferredoxin oxidoreductases, alpha subunit | Branching |
| [L] | COG0708 | Exonuclease III | Branching |
| [C] | COG0778 | Nitroreductase | Branching |
| [C] | COG1012 | NAD-dependent aldehyde dehydrogenases | Branching |
| [G] | COG1023 | Predicted 6-phosphogluconate dehydrogenase | Branching |
| [I] | COG1024 | Enoyl-CoA hydratase/carnithine racemase | Branching |
| [ER] | COG1063 | Threonine dehydrogenase and related Zn-dependent dehydrogenases | Branching |
| [R] | COG1647 | Esterase/lipase | Branching |
| [J] | COG1676 | tRNA splicing endonuclease | Branching |
| [I] | COG2030 | Acyl dehydratase | Branching |
| [F] | COG2131 | Deoxycytidylate deaminase | Branching |
| [G] | COG2133 | Glucose/sorbosone dehydrogenases | Branching |
| [I] | COG2134 | CDP-diacylglycerol pyrophosphatase | Branching |
| [H] | COG2154 | Pterin-4a-carbinolamine dehydratase | Branching |
| [R] | COG2333 | Predicted hydrolase (metallo-beta-lactamase superfamily) | Branching |
| [L] | COG2887 | RecB family exonuclease | Branching |
| [S] | COG3471 | Predicted periplasmic/secreted protein | Branching |
| [R] | COG3926 | Putative secretion activating protein | Branching |
| [H] | COG0113 | Delta-aminolevulinic acid dehydratase | Branching |
| [G] | COG0158 | Fructose-1,6-bisphosphatase | Branching |
| [H] | COG0190 | 5,10-methylene-tetrahydrofolate dehydrogenase/Methenyl tetrahydrofolate cyclohydrolase | Branching |
| [L] | COG0270 | Site-specific DNA methylase | Branching |
| [C] | COG0277 | FAD/FMN-containing dehydrogenases | Branching |
| [F] | COG0284 | Orotidine-5'-phosphate decarboxylase | Branching |
| [E] | COG0287 | Prephenate dehydrogenase | Branching |
| [P] | COG0288 | Carbonic anhydrase | Branching |
| [G] | COG0362 | 6-phosphogluconate dehydrogenase | Branching |
| [G] | COG0364 | Glucose-6-phosphate 1-dehydrogenase | Branching |
| [H] | COG0407 | Uroporphyrinogen-III decarboxylase | Branching |
| [C] | COG0427 | Acetyl-CoA hydrolase | Branching |
| [D] | COG0489 | ATPases involved in chromosome partitioning | Branching |
| [R] | COG0535 | Predicted Fe-S oxidoreductases | Branching |
| [K] | COG0557 | Exoribonuclease R | Branching |
| [FJ] | COG0590 | Cytosine/adenosine deaminases | Branching |
| [E] | COG0685 | 5,10-methylenetetrahydrofolate reductase | Branching |
| [E] | COG0686 | Alanine dehydrogenase | Branching |
| [I] | COG0688 | Phosphatidylserine decarboxylase | Branching |
| [R] | COG1407 | Predicted ICC-like phosphoesterases | Branching |
| [R] | COG1408 | Predicted phosphohydrolases | Branching |
| [R] | COG1409 | Predicted phosphohydrolases | Branching |
| [L] | COG1570 | Exonuclease VII, large subunit | Branching |
| [G] | COG1820 | N-acetylglucosamine-6-phosphate deacetylase | Branching |
| [C] | COG2033 | Desulfoferrodoxin | Branching |
| [C] | COG2055 | Malate/L-lactate dehydrogenases | Branching |
| [F] | COG2169 | Adenosine deaminase | Branching |
| [H] | COG2227 | 2-polyprenyl-3-methyl-5-hydroxy-6-metoxy-1,4-benzoquinol methylase | Branching |
| [E] | COG2515 | 1-aminocyclopropane-1-carboxylate deaminase | Branching |
| [L] | COG2925 | Exonuclease I | Branching |
| [EQ] | COG3191 | L-aminopeptidase/D-esterase | Branching |
| [R] | COG3608 | Predicted deacylase | Branching |
| [A] | COG5104 | Splicing factor | Branching |
| [A] | COG5161 | Pre-mRNA cleavage and polyadenylation specificity factor | Branching |
| [A] | COG5246 | Splicing factor 3a, subunit 2 | Branching |
| [M] | COG5520 | O-Glycosyl hydrolase | Branching |
| [F] | COG0026 | Phosphoribosylaminoimidazole carboxylase (NCAIR synthetase) | Branching |
| [H] | COG0117 | Pyrimidine deaminase | Branching |
| [EG] | COG0129 | Dihydroxyacid dehydratase/phosphogluconate dehydratase | Branching |
| [H] | COG0163 | 3-polyprenyl-4-hydroxybenzoate decarboxylase | Branching |
| [E] | COG0165 | Argininosuccinate lyase | Branching |
| [F] | COG0167 | Dihydroorotate dehydrogenase | Branching |
| [E] | COG0169 | Shikimate 5-dehydrogenase | Branching |
| [H] | COG0262 | Dihydrofolate reductase | Branching |
| [E] | COG0460 | Homoserine dehydrogenase | Branching |
| [C] | COG0538 | Isocitrate dehydrogenases | Branching |
| [L] | COG0608 | Single-stranded DNA-specific exonuclease | Branching |
| [M] | COG0845 | Membrane-fusion protein | Branching |
| [H] | COG0853 | Aspartate 1-decarboxylase | Branching |
| [I] | COG1257 | Hydroxymethylglutaryl-CoA reductase | Branching |
| [J] | COG1490 | D-Tyr-tRNAtyr deacylase | Branching |
| [Q] | COG1535 | Isochorismate hydrolase | Branching |
| [I] | COG1946 | Acyl-CoA thioesterase | Branching |
| [L] | COG1948 | ERCC4-type nuclease | Branching |
| [R] | COG2248 | Predicted hydrolase (metallo-beta-lactamase superfamily) | Branching |
| [C] | COG5557 | Polysulphide reductase | Branching |
| [C] | COG0039 | Malate/lactate dehydrogenases | Branching |
| [C] | COG0114 | Fumarase | Branching |
| [E] | COG0131 | Imidazoleglycerol-phosphate dehydratase | Branching |
| [E] | COG0136 | Aspartate-semialdehyde dehydrogenase | Branching |
| [E] | COG0139 | Phosphoribosyl-AMP cyclohydrolase | Branching |
| [E] | COG0140 | Phosphoribosyl-ATP pyrophosphohydrolase | Branching |
| [E] | COG0141 | Histidinol dehydrogenase | Branching |
| [H] | COG0181 | Porphobilinogen deaminase | Branching |
| [P] | COG0369 | Sulfite reductase, alpha subunit (flavoprotein) | Branching |
| [CE] | COG0473 | Isocitrate/isopropylmalate dehydrogenase | Branching |
| [C] | COG0578 | Glycerol-3-phosphate dehydrogenase | Branching |
| [R] | COG0579 | Predicted dehydrogenase | Branching |
| [R] | COG0673 | Predicted dehydrogenases and related proteins | Branching |
| [E] | COG0710 | 3-dehydroquinate dehydratase | Branching |
| [M] | COG1088 | dTDP-D-glucose 4,6-dehydratase | Branching |
| [M] | COG1089 | GDP-D-mannose dehydratase | Branching |
| [M] | COG1091 | dTDP-4-dehydrorhamnose reductase | Branching |
| [L] | COG1468 | RecB family exonuclease | Branching |
| [I] | COG1607 | Acyl-CoA hydrolase | Branching |
| [V] | COG1715 | Restriction endonuclease | Branching |
| [G] | COG1980 | Archaeal fructose 1,6-bisphosphatase | Branching |
| [I] | COG2272 | Carboxylesterase type B | Branching |
| [E] | COG2856 | Predicted Zn peptidase | Branching |
| [R] | COG3233 | Predicted deacetylase | Branching |
| [Q] | COG4308 | Limonene-1,2-epoxide hydrolase | Branching |
| [R] | COG4757 | Predicted alpha/beta hydrolase | Branching |
| [F] | COG0015 | Adenylosuccinate lyase | Branching |
| [E] | COG0019 | Diaminopimelate decarboxylase | Branching |
| [O] | COG0492 | Thioredoxin reductase | Branching |
| [H] | COG0499 | S-adenosylhomocysteine hydrolase | Branching |
| [E] | COG0506 | Proline dehydrogenase | Branching |
| [G] | COG0726 | Predicted xylanase/chitin deacetylase | Branching |
| [M] | COG0774 | UDP-3-O-acyl-N-acetylglucosamine deacetylase | Branching |
| [M] | COG1004 | Predicted UDP-glucose 6-dehydrogenase | Branching |
| [N] | COG1724 | Predicted periplasmic or secreted lipoprotein | Branching |
| [R] | COG5621 | Predicted secreted hydrolase | Branching |
| [G] | COG0021 | Transketolase | Branching |
| [R] | COG0388 | Predicted amidohydrolase | Branching |
| [R] | COG0400 | Predicted esterase | Branching |
| [I] | COG0743 | 1-deoxy-D-xylulose 5-phosphate reductoisomerase | Branching |
| [E] | COG0757 | 3-dehydroquinate dehydratase II | Branching |
| [M] | COG0860 | N-acetylmuramoyl-L-alanine amidase | Branching |
| [F] | COG1001 | Adenine deaminase | Branching |
| [L] | COG1722 | Exonuclease VII small subunit | Branching |
| [R] | COG2316 | Predicted hydrolase (HD superfamily) | Branching |
| [LDA] | COG5049 | 5'-3' exonuclease | Branching |
| [H] | COG0373 | Glutamyl-tRNA reductase | Branching |
| [M] | COG0677 | UDP-N-acetyl-D-mannosaminuronate dehydrogenase | Branching |
| [M] | COG0812 | UDP-N-acetylmuramate dehydrogenase | Branching |
| [E] | COG1586 | S-adenosylmethionine decarboxylase | Branching |
| [C] | COG4230 | Delta 1-pyrroline-5-carboxylate dehydrogenase | Branching |
| [A] | COG5188 | Splicing factor 3a, subunit 3 | Branching |
| [C] | COG0644 | Dehydrogenases (flavoproteins) | Branching |
| [C] | COG4229 | Predicted enolase-phosphatase | Branching |
| [E] | COG1171 | Threonine dehydratase | Branching |
| [NU] | COG4726 | Tfp pilus assembly protein PilX | Communication |
| [OU] | COG4960 | Flp pilus assembly protein, protease CpaA | Communication |
| [U] | COG4962 | Flp pilus assembly protein, ATPase CpaF | Communication |
| [U] | COG4963 | Flp pilus assembly protein, ATPase CpaE | Communication |
| [U] | COG4964 | Flp pilus assembly protein, secretin CpaC | Communication |
| [A] | COG5200 | U1 snRNP component, mediates U1 snRNP association with cap-binding complex | Communication |
| [K] | COG5211 | RNA polymerase II-interacting protein involved in transcription start site selection | Communication |
| [U] | COG4961 | Flp pilus assembly protein TadG | Communication |
| [U] | COG4965 | Flp pilus assembly protein TadB | Communication |
| [NU] | COG4966 | Tfp pilus assembly protein PilW | Communication |
| [L] | COG0353 | Recombinational DNA repair protein (RecF pathway) | Communication |
| [A] | COG5180 | Protein interacting with poly(A)-binding protein | Communication |
| [N] | COG5492 | Bacterial surface proteins containing Ig-like domains | Communication |
| [U] | COG5080 | Rab GTPase interacting factor, Golgi membrane protein | Communication |
| [K] | COG0250 | Transcription antiterminator | Communication |
| [M] | COG4775 | Outer membrane protein/protective antigen OMA87 | Communication |
| [S] | COG4716 | Myosin-crossreactive antigen | Communication |
| [NU] | COG4969 | Tfp pilus assembly protein, major pilin PilA | Communication |
| [N] | COG5461 | Type IV pili component | Communication |
| [NU] | COG4972 | Tfp pilus assembly protein, ATPase PilM | Communication |
| [U] | COG5010 | Flp pilus assembly protein TadD, contains TPR repeats | Communication |
| [M] | COG5386 | Cell surface protein | Communication |
| [NU] | COG4967 | Tfp pilus assembly protein PilV | Communication |
| [NU] | COG4968 | Tfp pilus assembly protein PilE | Communication |
| [NU] | COG4970 | Tfp pilus assembly protein FimT | Communication |
| [D] | COG5185 | Protein involved in chromosome segregation, interacts with SMC proteins | Communication |
| [NU] | COG5008 | Tfp pilus assembly protein, ATPase PilU | Communication |
| [M] | COG4520 | Surface antigen | Communication |
| [C] | COG3761 | NADH:ubiquinone oxidoreductase 17. Feb kD subunit | Degradation |
| [O] | COG4930 | Predicted ATP-dependent Lon-type protease | Degradation |
| [U] | COG5167 | Protein involved in vacuole import and degradation | Degradation |
| [T] | COG5432 | RING-finger-containing E3 ubiquitin ligase | Degradation |
| [O] | COG0330 | Membrane protease subunits, stomatin/prohibitin homologs | Degradation |
| [E] | COG1505 | Serine proteases of the peptidase family S9A | Degradation |
| [O] | COG5539 | Predicted cysteine protease (OTU family) | Degradation |
| [C] | COG0838 | NADH:ubiquinone oxidoreductase subunit 3 (chain A) | Degradation |
| [C] | COG0839 | NADH:ubiquinone oxidoreductase subunit 6 (chain J) | Degradation |
| [CP] | COG1009 | NADH:ubiquinone oxidoreductase subunit 5 (chain L)/Multisubunit Na+/H+ antiporter, MnhA subunit | Degradation |
| [O] | COG5661 | Predicted secreted Zn-dependent protease | Degradation |
| [O] | COG5664 | Predicted secreted Zn-dependent protease | Degradation |
| [O] | COG0533 | Metal-dependent proteases with possible chaperone activity | Degradation |
| [C] | COG2878 | Predicted NADH:ubiquinone oxidoreductase, subunit RnfB | Degradation |
| [R] | COG3654 | Prophage maintenance system killer protein | Degradation |
| [P] | COG3720 | Putative heme degradation protein | Degradation |
| [C] | COG4313 | Protein involved in meta-pathway of phenol degradation | Degradation |
| [C] | COG4656 | Predicted NADH:ubiquinone oxidoreductase, subunit RnfC | Degradation |
| [C] | COG4657 | Predicted NADH:ubiquinone oxidoreductase, subunit RnfA | Degradation |
| [C] | COG4658 | Predicted NADH:ubiquinone oxidoreductase, subunit RnfD | Degradation |
| [C] | COG4659 | Predicted NADH:ubiquinone oxidoreductase, subunit RnfG | Degradation |
| [C] | COG4660 | Predicted NADH:ubiquinone oxidoreductase, subunit RnfE | Degradation |
| [O] | COG5504 | Predicted Zn-dependent protease | Degradation |
| [O] | COG5533 | Ubiquitin C-terminal hydrolase | Degradation |
| [O] | COG5540 | RING-finger-containing ubiquitin ligase | Degradation |
| [E] | COG0520 | Selenocysteine lyase | Degradation |
| [C] | COG0852 | NADH:ubiquinone oxidoreductase 27 kD subunit | Degradation |
| [O] | COG1030 | Membrane-bound serine protease (ClpP class) | Degradation |
| [R] | COG1750 | Archaeal serine proteases | Degradation |
| [R] | COG3549 | Plasmid maintenance system killer protein | Degradation |
| [R] | COG3772 | Phage-related lysozyme (muraminidase) | Degradation |
| [R] | COG4287 | PhoPQ-activated pathogenicity-related protein | Degradation |
| [R] | COG4900 | Predicted metallopeptidase | Degradation |
| [O] | COG5243 | HRD ubiquitin ligase complex, ER membrane component | Degradation |
| [O] | COG5640 | Secreted trypsin-like serine protease | Degradation |
| [O] | COG5647 | Cullin, a subunit of E3 ubiquitin ligase | Degradation |
| [O] | COG0466 | ATP-dependent Lon protease, bacterial type | Degradation |
| [OU] | COG0616 | Periplasmic serine proteases (ClpP class) | Degradation |
| [R] | COG3607 | Predicted lactoylglutathione lyase | Degradation |
| [O] | COG5201 | SCF ubiquitin ligase, SKP1 component | Degradation |
| [C] | COG0281 | Malic enzyme | Degradation |
| [O] | COG0465 | ATP-dependent Zn proteases | Degradation |
| [M] | COG0793 | Periplasmic protease | Degradation |
| [C] | COG1005 | NADH:ubiquinone oxidoreductase subunit 1 (chain H) | Degradation |
| [C] | COG1007 | NADH:ubiquinone oxidoreductase subunit 2 (chain N) | Degradation |
| [C] | COG1008 | NADH:ubiquinone oxidoreductase subunit 4 (chain M) | Degradation |
| [O] | COG1404 | Subtilisin-like serine proteases | Degradation |
| [E] | COG1506 | Dipeptidyl aminopeptidases/acylaminoacyl-peptidases | Degradation |
| [P] | COG1513 | Cyanate lyase | Degradation |
| [C] | COG1905 | NADH:ubiquinone oxidoreductase 24 kD subunit | Degradation |
| [C] | COG2224 | Isocitrate lyase | Degradation |
| [R] | COG2738 | Predicted Zn-dependent protease | Degradation |
| [R] | COG3577 | Predicted aspartyl protease | Degradation |
| [E] | COG4187 | Arginine degradation protein (predicted deacylase) | Degradation |
| [R] | COG4784 | Putative Zn-dependent protease | Degradation |
| [O] | COG5131 | Ubiquitin-like protein | Degradation |
| [O] | COG5549 | Predicted Zn-dependent protease | Degradation |
| [O] | COG5550 | Predicted aspartyl protease | Degradation |
| [C] | COG1882 | Pyruvate-formate lyase | Degradation |
| [O] | COG5021 | Ubiquitin-protein ligase | Degradation |
| [O] | COG5077 | Ubiquitin carboxyl-terminal hydrolase | Degradation |
| [O] | COG5574 | RING-finger-containing E3 ubiquitin ligase | Degradation |
| [O] | COG0638 | 20S proteasome, alpha and beta subunits | Degradation |
| [E] | COG1770 | Protease II | Degradation |
| [O] | COG5078 | Ubiquitin-protein ligase | Degradation |
| [O] | COG5140 | Ubiquitin fusion-degradation protein | Degradation |
| [OD] | COG5194 | Component of SCF ubiquitin ligase and anaphase-promoting complex | Degradation |
| [O] | COG5560 | Ubiquitin C-terminal hydrolase | Degradation |
| [R] | COG3975 | Predicted protease with the C-terminal PDZ domain | Degradation |
| [R] | COG4740 | Predicted metalloprotease | Degradation |
| [R] | COG5342 | Invasion protein B, involved in pathogenesis | Degradation |
| [O] | COG4934 | Predicted protease | Degradation |
| [D] | COG4942 | Membrane-bound metallopeptidase | Degradation |
| [O] | COG5227 | Ubiquitin-like protein (sentrin) | Degradation |
| [O] | COG5272 | Ubiquitin | Degradation |
| [M] | COG1044 | UDP-3-O-[3-hydroxymyristoyl] glucosamine N-acyltransferase" | Transport_container |
| [M] | COG3773 | Cell wall hydrolyses involved in spore germination | Information_storage |
| [R] | COG4397 | Mu-like prophage major head subunit gpT | Information_storage |
| [G] | COG4580 | Maltoporin (phage lambda and maltose receptor) | Information_storage |
| [M] | COG4623 | Predicted soluble lytic transglycosylase fused to an ABC-type amino acid-binding protein | Information_storage |
| [R] | COG5366 | Protein involved in propagation of M2 dsRNA satellite of L-A virus | Information_storage |
| [R] | COG3497 | Phage tail sheath protein FI | Information_storage |
| [R] | COG3498 | Phage tail tube protein FII | Information_storage |
| [R] | COG3740 | Phage head maturation protease | Information_storage |
| [R] | COG4326 | Sporulation control protein | Information_storage |
| [R] | COG4653 | Predicted phage phi-C31 gp36 major capsid-like protein | Information_storage |
| [S] | COG4723 | Phage-related protein, tail component | Information_storage |
| [S] | COG4733 | Phage-related protein, tail component | Information_storage |
| [UI] | COG5153 | Putative lipase essential for disintegration of autophagic bodies inside the vacuole | Information_storage |
| [M] | COG5577 | Spore coat protein | Information_storage |
| [L] | COG4220 | Phage DNA packaging protein, Nu1 subunit of terminase | Information_storage |
| [R] | COG4386 | Mu-like prophage tail sheath protein gpL | Information_storage |
| [S] | COG4387 | Mu-like prophage protein gp36 | Information_storage |
| [R] | COG4388 | Mu-like prophage I protein | Information_storage |
| [G] | COG4678 | Muramidase (phage lambda lysozyme) | Information_storage |
| [S] | COG4926 | Phage-related protein | Information_storage |
| [R] | COG3499 | Phage protein U | Information_storage |
| [R] | COG3500 | Phage protein D | Information_storage |
| [R] | COG4373 | Mu-like prophage FluMu protein gp28 | Information_storage |
| [R] | COG4379 | Mu-like prophage tail protein gpP | Information_storage |
| [R] | COG4385 | Bacteriophage P2-related tail formation protein | Information_storage |
| [S] | COG4718 | Phage-related protein | Information_storage |
| [S] | COG4722 | Phage-related protein | Information_storage |
| [R] | COG5302 | Post-segregation antitoxin (ccd killing mechanism protein) encoded by the F plasmid | Information_storage |
| [L] | COG5433 | Transposase | Information_storage |
| [R] | COG5511 | Bacteriophage capsid protein | Information_storage |
| [R] | COG5565 | Bacteriophage terminase large (ATPase) subunit and inactivated derivatives | Information_storage |
| [K] | COG3561 | Phage anti-repressor protein | Information_storage |
| [S] | COG4381 | Mu-like prophage protein gp46 | Information_storage |
| [S] | COG4382 | Mu-like prophage protein gp16 | Information_storage |
| [S] | COG4383 | Mu-like prophage protein gp29 | Information_storage |
| [S] | COG4384 | Mu-like prophage protein gp45 | Information_storage |
| [S] | COG4672 | Phage-related protein | Information_storage |
| [S] | COG4679 | Phage-related protein | Information_storage |
| [V] | COG4823 | Abortive infection bacteriophage resistance protein | Information_storage |
| [R] | COG5518 | Bacteriophage capsid portal protein | Information_storage |
| [R] | COG5525 | Bacteriophage tail assembly protein | Information_storage |
| [L] | COG5659 | FOG: Transposase | Information_storage |
| [D] | COG2385 | Sporulation protein and related proteins | Information_storage |
| [R] | COG4824 | Phage-related holin (Lysis protein) | Information_storage |
| [R] | COG5401 | Spore germination protein | Information_storage |
| [R] | COG3941 | Mu-like prophage protein | Information_storage |
| [R] | COG3948 | Phage-related baseplate assembly protein | Information_storage |
| [R] | COG4626 | Phage terminase-like protein, large subunit | Information_storage |
| [L] | COG4644 | Transposase and inactivated derivatives, TnpA family | Information_storage |
| [S] | COG5412 | Phage-related protein | Information_storage |
| [S] | COG4518 | Mu-like prophage FluMu protein gp41 | Information_storage |
| [L] | COG5377 | Phage-related protein, predicted endonuclease | Information_storage |
| [L] | COG5421 | Transposase | Information_storage |
| [R] | COG4540 | Phage P2 baseplate assembly protein gpV | Information_storage |
| [R] | COG4691 | Plasmid stability protein | Information_storage |
| [S] | COG5280 | Phage-related minor tail protein | Information_storage |
| [L] | COG5558 | Transposase | Information_storage |
| [R] | COG5562 | Phage envelope protein | Information_storage |
| [R] | COG4228 | Mu-like prophage DNA circulation protein | Information_storage |
| [S] | COG4695 | Phage-related protein | Information_storage |
| [R] | COG5004 | P2-like prophage tail protein X | Information_storage |
| [R] | COG5362 | Phage-related terminase | Information_storage |
| [R] | COG5614 | Bacteriophage head-tail adaptor | Information_storage |
| [R] | COG5003 | Mu-like prophage protein gp37 | Information_storage |
| [R] | COG5005 | Mu-like prophage protein gpG | Information_storage |
| [S] | COG5281 | Phage-related minor tail protein | Information_storage |
| [R] | COG5301 | Phage-related tail fibre protein | Information_storage |
| [S] | COG5283 | Phage-related tail protein | Information_storage |
| [M] | COG5337 | Spore coat assembly protein | Information_storage |
| [D] | COG0424 | Nucleotide-binding protein implicated in inhibition of septum formation | Inhibition |
| [T] | COG2972 | Predicted signal transduction protein with a C-terminal ATPase domain | Inhibition |
| [C] | COG3005 | Nitrate/TMAO reductases, membrane-bound tetraheme cytochrome c subunit | Inhibition |
| [T] | COG3073 | Negative regulator of sigma E activity | Inhibition |
| [UNTP] | COG3678 | P pilus assembly/Cpx signaling pathway, periplasmic inhibitor/zinc-resistance associated protein | Inhibition |
| [R] | COG4396 | Mu-like prophage host-nuclease inhibitor protein Gam | Inhibition |
| [E] | COG4819 | Ethanolamine utilization protein, possible chaperonin protecting lyase from inhibition | Inhibition |
| [J] | COG5099 | RNA-binding protein of the Puf family, translational repressor | Inhibition |
| [DK] | COG5103 | Cell division control protein, negative regulator of transcription | Inhibition |
| [D] | COG5404 | SOS-response cell division inhibitor, blocks FtsZ ring formation | Inhibition |
| [N] | COG1406 | Predicted inhibitor of MCP methylation, homolog of CheC | Inhibition |
| [C] | COG2009 | Succinate dehydrogenase/fumarate reductase, cytochrome b subunit | Inhibition |
| [K] | COG5601 | General negative regulator of transcription subunit | Inhibition |
| [R] | COG1245 | Predicted ATPase, RNase L inhibitor (RLI) homolog | Inhibition |
| [P] | COG2920 | Dissimilatory sulfite reductase (desulfoviridin), gamma subunit | Inhibition |
| [T] | COG3026 | Negative regulator of sigma E activity | Inhibition |
| [K] | COG4463 | Transcriptional repressor of class III stress genes | Inhibition |
| [R] | COG4574 | Serine protease inhibitor ecotin | Inhibition |
| [K] | COG5247 | Class 2 transcription repressor NC2, alpha subunit (DRAP1 homolog) | Inhibition |
| [L] | COG5519 | Superfamily II helicase and inactivated derivatives | Inhibition |
| [L] | COG3057 | Negative regulator of replication initiationR | Inhibition |
| [L] | COG3449 | DNA gyrase inhibitor | Inhibition |
| [OTN] | COG4862 | Negative regulator of genetic competence, sporulation and motility | Inhibition |
| [K] | COG2740 | Predicted nucleic-acid-binding protein implicated in transcription termination | Inhibition |
| [D] | COG2894 | Septum formation inhibitor-activating ATPase | Inhibition |
| [C] | COG3029 | Fumarate reductase subunit C | Inhibition |
| [K] | COG4465 | Pleiotropic transcriptional repressor | Inhibition |
| [K] | COG4568 | Transcriptional antiterminator | Inhibition |
| [K] | COG5150 | Class 2 transcription repressor NC2, beta subunit (Dr1) | Inhibition |
| [K] | COG2732 | Barstar, RNAse (barnase) inhibitor | Inhibition |
| [R] | COG2910 | Putative NADH-flavin reductase | Inhibition |
| [K] | COG3711 | Transcriptional antiterminator | Inhibition |
| [R] | COG5091 | Suppressor of G2 allele of skp1 and related proteins | Inhibition |
| [K] | COG5175 | Transcriptional repressor | Inhibition |
| [D] | COG0850 | Septum formation inhibitor | Inhibition |
| [K] | COG2973 | Trp operon repressor | Inhibition |
| [O] | COG4826 | Serine protease inhibitor | Inhibition |
| [T] | COG2337 | Growth inhibitor | Inhibition |
| [L] | COG3436 | Transposase and inactivated derivatives | Inhibition |
| [K] | COG3933 | Transcriptional antiterminator | Inhibition |
| [DZ] | COG5184 | Alpha-tubulin suppressor and related RCC1 domain-containing proteins | Inhibition |
| [KD] | COG5189 | Putative transcriptional repressor regulating G2/M transition | Inhibition |
| [J] | COG0522 | Ribosomal protein S4 and related proteins | Linear |
| [FGR] | COG0537 | Diadenosine tetraphosphate (Ap4A) hydrolase and other HIT family hydrolases | Linear |
| [P] | COG0783 | DNA-binding ferritin-like protein (oxidative damage protectant) | Linear |
| [C] | COG1013 | Pyruvate:ferredoxin oxidoreductase and related 2-oxoacid:ferredoxin oxidoreductases, beta subunit | Linear |
| [C] | COG1014 | Pyruvate:ferredoxin oxidoreductase and related 2-oxoacid:ferredoxin oxidoreductases, gamma subunit | Linear |
| [R] | COG1439 | Predicted nucleic acid-binding protein, consists of a PIN domain and a Zn-ribbon module | Linear |
| [R] | COG1487 | Predicted nucleic acid-binding protein, contains PIN domain | Linear |
| [K] | COG1644 | DNA-directed RNA polymerase, subunit N (RpoN/RPB10) | Linear |
| [V] | COG1680 | Beta-lactamase class C and other penicillin binding proteins | Linear |
| [R] | COG1782 | Predicted metal-dependent RNase, consists of a metallo-beta-lactamase domain and an RNA-binding KH domain | Linear |
| [R] | COG1827 | Predicted small molecule binding protein (contains 3H domain) | Linear |
| [R] | COG1853 | Conserved protein/domain typically associated with flavoprotein oxygenases, DIM6/NTAB family | Linear |
| [JD] | COG2026 | Cytotoxic translational repressor of toxin-antitoxin stability system | Linear |
| [R] | COG2110 | Predicted phosphatase homologous to the C-terminal domain of histone macroH2A1 | Linear |
| [R] | COG2129 | Predicted phosphoesterases, related to the Icc protein | Linear |
| [J] | COG2451 | Ribosomal protein L35AE/L33A | Linear |
| [T] | COG2453 | Predicted protein-tyrosine phosphatase | Linear |
| [R] | COG2605 | Predicted kinase related to galactokinase and mevalonate kinase | Linear |
| [L] | COG2816 | NTP pyrophosphohydrolases containing a Zn-finger, probably nucleic-acid-binding | Linear |
| [Q] | COG2931 | RTX toxins and related Ca2+-binding proteins | Linear |
| [R] | COG3417 | Collagen-binding surface adhesin SpaP (antigen I/II family) | Linear |
| [R] | COG3478 | Predicted nucleic-acid-binding protein containing a Zn-ribbon domain | Linear |
| [R] | COG3529 | Predicted nucleic-acid-binding protein containing a Zn-ribbon domain | Linear |
| [R] | COG3576 | Predicted flavin-nucleotide-binding protein structurally related to pyridoxine 5'-phosphate oxidase | Linear |
| [R] | COG4277 | Predicted DNA-binding protein with the Helix-hairpin-helix motif | Linear |
| [R] | COG4710 | Predicted DNA-binding protein with an HTH domain | Linear |
| [R] | COG5516 | Conserved protein containing a Zn-ribbon-like motif, possibly RNA-binding | Linear |
| [E] | COG0241 | Histidinol phosphatase and related phosphatases | Linear |
| [T] | COG0478 | RIO-like serine/threonine protein kinase fused to N-terminal HTH domain | Linear |
| [P] | COG0529 | Adenylylsulfate kinase and related kinases | Linear |
| [L] | COG0551 | Zn-finger domain associated with topoisomerase type I | Linear |
| [G] | COG0647 | Predicted sugar phosphatases of the HAD superfamily | Linear |
| [R] | COG1453 | Predicted oxidoreductases of the aldo/keto reductase family | Linear |
| [R] | COG1646 | Predicted phosphate-binding enzymes, TIM-barrel fold | Linear |
| [J] | COG1825 | Ribosomal protein L25 (general stress protein Ctc) | Linear |
| [J] | COG2016 | Predicted RNA-binding protein (contains PUA domain) | Linear |
| [J] | COG2178 | Predicted RNA-binding protein of the translin family | Linear |
| [J] | COG3277 | RNA-binding protein involved in rRNA processing | Linear |
| [R] | COG3318 | Predicted metal-binding protein related to the C-terminal domain of SecA | Linear |
| [R] | COG3889 | Predicted solute binding protein | Linear |
| [R] | COG4031 | Predicted metal-binding protein | Linear |
| [R] | COG4113 | Predicted nucleic acid-binding protein, contains PIN domain | Linear |
| [S] | COG5466 | Predicted small metal-binding protein | Linear |
| [H] | COG0301 | Thiamine biosynthesis ATP pyrophosphatase | Linear |
| [L] | COG0338 | Site-specific DNA methylase | Linear |
| [H] | COG0340 | Biotin-(acetyl-CoA carboxylase) ligase | Linear |
| [L] | COG0417 | DNA polymerase elongation subunit (family B) | Linear |
| [G] | COG0524 | Sugar kinases, ribokinase family | Linear |
| [KL] | COG0553 | Superfamily II DNA/RNA helicases, SNF2 family | Linear |
| [C] | COG0713 | NADH:ubiquinone oxidoreductase subunit 11 or 4L (chain K) | Linear |
| [L] | COG0776 | Bacterial nucleoid DNA-binding protein | Linear |
| [C] | COG1625 | Fe-S oxidoreductase, related to NifB/MoaA family | Linear |
| [R] | COG1661 | Predicted DNA-binding protein with PD1-like DNA-binding motif | Linear |
| [K] | COG1758 | DNA-directed RNA polymerase, subunit K/omega | Linear |
| [R] | COG1818 | Predicted RNA-binding protein, contains THUMP domain | Linear |
| [M] | COG2027 | D-alanyl-D-alanine carboxypeptidase (penicillin-binding protein 4) | Linear |
| [R] | COG3380 | Predicted NAD/FAD-dependent oxidoreductase | Linear |
| [R] | COG3688 | Predicted RNA-binding protein containing a PIN domain | Linear |
| [R] | COG4892 | Predicted heme/steroid binding protein | Linear |
| [OU] | COG5061 | Oxidoreductin, endoplasmic reticulum membrane-associated protein involved in disulfide bond formation | Linear |
| [R] | COG5263 | FOG: Glucan-binding domain (YG repeat) | Linear |
| [S] | COG5469 | Predicted metal-binding protein | Linear |
| [G] | COG0058 | Glucan phosphorylase | Linear |
| [J] | COG0211 | Ribosomal protein L27 | Linear |
| [J] | COG0222 | Ribosomal protein L7/L12 | Linear |
| [J] | COG0244 | Ribosomal protein L10 | Linear |
| [J] | COG0254 | Ribosomal protein L31 | Linear |
| [J] | COG0255 | Ribosomal protein L29 | Linear |
| [J] | COG0291 | Ribosomal protein L35 | Linear |
| [J] | COG0292 | Ribosomal protein L20 | Linear |
| [H] | COG0302 | GTP cyclohydrolase I | Linear |
| [H] | COG0321 | Lipoate-protein ligase B | Linear |
| [J] | COG0333 | Ribosomal protein L32 | Linear |
| [J] | COG0335 | Ribosomal protein L19 | Linear |
| [E] | COG0339 | Zn-dependent oligopeptidases | Linear |
| [J] | COG0349 | Ribonuclease D | Linear |
| [H] | COG0351 | Hydroxymethylpyrimidine/phosphomethylpyrimidine kinase | Linear |
| [HC] | COG0543 | 2-polyprenylphenol hydroxylase and related flavodoxin oxidoreductases | Linear |
| [F] | COG0563 | Adenylate kinase and related kinases | Linear |
| [L] | COG0587 | DNA polymerase III, alpha subunit | Linear |
| [E] | COG0831 | Urea amidohydrolase (urease) gamma subunit | Linear |
| [E] | COG0832 | Urea amidohydrolase (urease) beta subunit | Linear |
| [R] | COG1399 | Predicted metal-binding, possibly nucleic acid-binding protein | Linear |
| [L] | COG1555 | DNA uptake protein and related DNA-binding proteins | Linear |
| [R] | COG1569 | Predicted nucleic acid-binding protein, contains PIN domain | Linear |
| [R] | COG1837 | Predicted RNA-binding protein (contains KH domain) | Linear |
| [R] | COG2041 | Sulfite oxidase and related enzymes | Linear |
| [HR] | COG2045 | Phosphosulfolactate phosphohydrolase and related enzymes | Linear |
| [J] | COG2125 | Ribosomal protein S6E (S10) | Linear |
| [J] | COG2157 | Ribosomal protein L20A (L18A) | Linear |
| [R] | COG2405 | Predicted nucleic acid-binding protein, contains PIN domain | Linear |
| [M] | COG3409 | Putative peptidoglycan-binding domain-containing protein | Linear |
| [T] | COG4579 | Isocitrate dehydrogenase kinase/phosphatase | Linear |
| [R] | COG5595 | Zn-ribbon-containing, possibly nucleic-acid-binding protein | Linear |
| [J] | COG0197 | Ribosomal protein L16/L10E | Linear |
| [J] | COG0198 | Ribosomal protein L24 | Linear |
| [J] | COG0199 | Ribosomal protein S14 | Linear |
| [J] | COG0200 | Ribosomal protein L15 | Linear |
| [J] | COG0203 | Ribosomal protein L17 | Linear |
| [F] | COG0213 | Thymidine phosphorylase | Linear |
| [C] | COG0221 | Inorganic pyrophosphatase | Linear |
| [L] | COG0328 | Ribonuclease HI | Linear |
| [GEPR] | COG0477 | Permeases of the major facilitator superfamily | Linear |
| [LR] | COG0494 | NTP pyrophosphohydrolases including oxidative damage repair enzymes | Linear |
| [F] | COG0528 | Uridylate kinase | Linear |
| [K] | COG0568 | DNA-directed RNA polymerase, sigma subunit (sigma70/sigma32) | Linear |
| [R] | COG0658 | Predicted membrane metal-binding protein | Linear |
| [R] | COG0661 | Predicted unusual protein kinase | Linear |
| [F] | COG0775 | Nucleoside phosphorylase | Linear |
| [I] | COG0825 | Acetyl-CoA carboxylase alpha subunit | Linear |
| [O] | COG0826 | Collagenase and related proteases | Linear |
| [O] | COG0829 | Urease accessory protein UreH | Linear |
| [O] | COG0830 | Urease accessory protein UreF | Linear |
| [R] | COG1011 | Predicted hydrolase (HAD superfamily) | Linear |
| [O] | COG1025 | Secreted/periplasmic Zn-dependent peptidases, insulinase-like | Linear |
| [R] | COG1026 | Predicted Zn-dependent peptidases, insulinase-like | Linear |
| [R] | COG1489 | DNA-binding protein, stimulates sugar fermentation | Linear |
| [O] | COG1495 | Disulfide bond formation protein DsbB | Linear |
| [R] | COG1545 | Predicted nucleic-acid-binding protein containing a Zn-ribbon | Linear |
| [R] | COG1964 | Predicted Fe-S oxidoreductases | Linear |
| [F] | COG2019 | Archaeal adenylate kinase | Linear |
| [K] | COG2093 | DNA-directed RNA polymerase, subunit E'' | Linear |
| [J] | COG2126 | Ribosomal protein L37E | Linear |
| [J] | COG2163 | Ribosomal protein L14E/L6E/L27E | Linear |
| [R] | COG3113 | Predicted NTP binding protein (contains STAS domain) | Linear |
| [C] | COG3488 | Predicted thiol oxidoreductase | Linear |
| [K] | COG3710 | DNA-binding winged-HTH domains | Linear |
| [R] | COG4001 | Predicted metal-binding protein | Linear |
| [Z] | COG5069 | Ca2+-binding actin-bundling protein fimbrin/plastin (EF-Hand superfamily) | Linear |
| [TZDR] | COG5126 | Ca2+-binding protein (EF-Hand superfamily) | Linear |
| [H] | COG0157 | Nicotinate-nucleotide pyrophosphorylase | Linear |
| [F] | COG0194 | Guanylate kinase | Linear |
| [H] | COG0276 | Protoheme ferro-lyase (ferrochelatase) | Linear |
| [P] | COG0306 | Phosphate/sulphate permeases | Linear |
| [E] | COG0308 | Aminopeptidase N | Linear |
| [J] | COG0359 | Ribosomal protein L9 | Linear |
| [J] | COG0360 | Ribosomal protein S6 | Linear |
| [H] | COG0408 | Coproporphyrinogen III oxidase | Linear |
| [G] | COG0448 | ADP-glucose pyrophosphorylase | Linear |
| [F] | COG0503 | Adenine/guanine phosphoribosyltransferases and related PRPP-binding proteins | Linear |
| [LKJ] | COG0513 | Superfamily II DNA and RNA helicases | Linear |
| [E] | COG0527 | Aspartokinases | Linear |
| [C] | COG0584 | Glycerophosphoryl diester phosphodiesterase | Linear |
| [R] | COG0655 | Multimeric flavodoxin WrbA | Linear |
| [M] | COG0768 | Cell division protein FtsI/penicillin-binding protein 2 | Linear |
| [L] | COG0827 | Adenine-specific DNA methylase | Linear |
| [J] | COG0828 | Ribosomal protein S21 | Linear |
| [Q] | COG1021 | Peptide arylation enzymes | Linear |
| [C] | COG1029 | Formylmethanofuran dehydrogenase subunit B | Linear |
| [R] | COG1458 | Predicted DNA-binding protein containing PIN domain | Linear |
| [J] | COG1514 | 2'-5' RNA ligase | Linear |
| [R] | COG1579 | Zn-ribbon protein, possibly nucleic acid-binding | Linear |
| [C] | COG1902 | NADH:flavin oxidoreductases, Old Yellow Enzyme family | Linear |
| [P] | COG1910 | Periplasmic molybdate-binding protein/domain | Linear |
| [E] | COG2040 | Homocysteine/selenocysteine methylase (S-methylmethionine-dependent) | Linear |
| [J] | COG2051 | Ribosomal protein S27E | Linear |
| [J] | COG2053 | Ribosomal protein S28E/S33 | Linear |
| [J] | COG2058 | Ribosomal protein L12E/L44/L45/RPP1/RPP2 | Linear |
| [T] | COG2062 | Phosphohistidine phosphatase SixA | Linear |
| [J] | COG2075 | Ribosomal protein L24E | Linear |
| [R] | COG2085 | Predicted dinucleotide-binding enzymes | Linear |
| [J] | COG2167 | Ribosomal protein L39E | Linear |
| [G] | COG2182 | Maltose-binding periplasmic proteins/domains | Linear |
| [E] | COG2362 | D-aminopeptidase | Linear |
| [T] | COG2365 | Protein tyrosine/serine phosphatase | Linear |
| [R] | COG2907 | Predicted NAD/FAD-binding protein | Linear |
| [R] | COG2916 | DNA-binding protein H-NS | Linear |
| [R] | COG3341 | Predicted double-stranded RNA/RNA-DNA hybrid binding protein | Linear |
| [R] | COG3467 | Predicted flavin-nucleotide-binding protein | Linear |
| [Q] | COG3486 | Lysine/ornithine N-monooxygenase | Linear |
| [C] | COG4263 | Nitrous oxide reductase | Linear |
| [I] | COG4281 | Acyl-CoA-binding protein | Linear |
| [J] | COG4901 | Ribosomal protein S25 | Linear |
| [J] | COG5045 | Ribosomal protein S10E | Linear |
| [S] | COG5423 | Predicted metal-binding protein | Linear |
| [S] | COG5629 | Predicted metal-binding protein | Linear |
| [G] | COG0153 | Galactokinase | Linear |
| [G] | COG0205 | 6-phosphofructokinase | Linear |
| [J] | COG0227 | Ribosomal protein L28 | Linear |
| [J] | COG0228 | Ribosomal protein S16 | Linear |
| [J] | COG0230 | Ribosomal protein L34 | Linear |
| [J] | COG0238 | Ribosomal protein S18 | Linear |
| [J] | COG0256 | Ribosomal protein L18 | Linear |
| [J] | COG0257 | Ribosomal protein L36 | Linear |
| [J] | COG0261 | Ribosomal protein L21 | Linear |
| [J] | COG0267 | Ribosomal protein L33 | Linear |
| [J] | COG0268 | Ribosomal protein S20 | Linear |
| [O] | COG0278 | Glutaredoxin-related protein | Linear |
| [C] | COG0282 | Acetate kinase | Linear |
| [F] | COG0283 | Cytidylate kinase | Linear |
| [L] | COG0415 | Deoxyribodipyrimidine photolyase | Linear |
| [J] | COG0539 | Ribosomal protein S1 | Linear |
| [E] | COG0548 | Acetylglutamate kinase | Linear |
| [E] | COG0549 | Carbamate kinase | Linear |
| [C] | COG0554 | Glycerol kinase | Linear |
| [E] | COG0560 | Phosphoserine phosphatase | Linear |
| [G] | COG0574 | Phosphoenolpyruvate synthase/pyruvate phosphate dikinase | Linear |
| [L] | COG0648 | Endonuclease IV | Linear |
| [R] | COG0679 | Predicted permeases | Linear |
| [R] | COG0724 | RNA-binding proteins (RRM domain) | Linear |
| [R] | COG0824 | Predicted thioesterase | Linear |
| [H] | COG1010 | Precorrin-3B methylase | Linear |
| [C] | COG1017 | Hemoglobin-like flavoprotein | Linear |
| [G] | COG1070 | Sugar (pentulose and hexulose) kinases | Linear |
| [R] | COG1073 | Hydrolases of the alpha/beta superfamily | Linear |
| [J] | COG1491 | Predicted RNA-binding protein | Linear |
| [R] | COG1532 | Predicted RNA-binding protein | Linear |
| [F] | COG1816 | Adenosine deaminase | Linear |
| [R] | COG1832 | Predicted CoA-binding protein | Linear |
| [R] | COG1847 | Predicted RNA-binding protein | Linear |
| [A] | COG1949 | Oligoribonuclease (3'->5' exoribonuclease) | Linear |
| [R] | COG2005 | N-terminal domain of molybdenum-binding protein | Linear |
| [R] | COG2044 | Predicted peroxiredoxins | Linear |
| [G] | COG2074 | 2-phosphoglycerate kinase | Linear |
| [L] | COG2094 | 3-methyladenine DNA glycosylase | Linear |
| [J] | COG2097 | Ribosomal protein L31E | Linear |
| [R] | COG2100 | Predicted Fe-S oxidoreductase | Linear |
| [R] | COG2107 | Predicted periplasmic solute-binding protein | Linear |
| [T] | COG2112 | Predicted Ser/Thr protein kinase | Linear |
| [H] | COG2145 | Hydroxyethylthiazole kinase, sugar kinase family | Linear |
| [R] | COG2344 | AT-rich DNA-binding protein | Linear |
| [G] | COG3001 | Fructosamine-3-kinase | Linear |
| [J] | COG4830 | Ribosomal protein S26 | Linear |
| [L] | COG0164 | Ribonuclease HII | Linear |
| [I] | COG0170 | Dolichol kinase | Linear |
| [J] | COG0184 | Ribosomal protein S15P/S13E | Linear |
| [J] | COG0185 | Ribosomal protein S19 | Linear |
| [J] | COG0186 | Ribosomal protein S17 | Linear |
| [F] | COG0232 | dGTP triphosphohydrolase | Linear |
| [H] | COG0237 | Dephospho-CoA kinase | Linear |
| [H] | COG0259 | Pyridoxamine-phosphate oxidase | Linear |
| [E] | COG0260 | Leucyl aminopeptidase | Linear |
| [E] | COG0263 | Glutamate 5-kinase | Linear |
| [L] | COG0266 | Formamidopyrimidine-DNA glycosylase | Linear |
| [G] | COG0366 | Glycosidases | Linear |
| [F] | COG0418 | Dihydroorotase | Linear |
| [L] | COG0514 | Superfamily II DNA helicase | Linear |
| [R] | COG0536 | Predicted GTPase | Linear |
| [R] | COG0546 | Predicted phosphatases | Linear |
| [E] | COG0547 | Anthranilate phosphoribosyltransferase | Linear |
| [H] | COG0611 | Thiamine monophosphate kinase | Linear |
| [H] | COG0635 | Coproporphyrinogen III oxidase and related Fe-S oxidoreductases | Linear |
| [T] | COG0639 | Diadenosine tetraphosphatase and related serine/threonine protein phosphatases | Linear |
| [C] | COG0667 | Predicted oxidoreductases (related to aryl-alcohol dehydrogenases) | Linear |
| [O] | COG0678 | Peroxiredoxin | Linear |
| [C] | COG0723 | Rieske Fe-S protein | Linear |
| [G] | COG0837 | Glucokinase | Linear |
| [P] | COG0855 | Polyphosphate kinase | Linear |
| [G] | COG1015 | Phosphopentomutase | Linear |
| [E] | COG1027 | Aspartate ammonia-lyase | Linear |
| [C] | COG1032 | Fe-S oxidoreductase | Linear |
| [R] | COG1537 | Predicted RNA-binding proteins | Linear |
| [K] | COG1581 | Archaeal DNA-binding protein | Linear |
| [R] | COG1719 | Predicted hydrocarbon binding protein (contains V4R domain) | Linear |
| [R] | COG1881 | Phospholipid-binding protein | Linear |
| [H] | COG1893 | Ketopantoate reductase | Linear |
| [E] | COG2066 | Glutaminase | Linear |
| [O] | COG2077 | Peroxiredoxin | Linear |
| [H] | COG2099 | Precorrin-6x reductase | Linear |
| [L] | COG2189 | Adenine specific DNA methylase Mod | Linear |
| [J] | COG2238 | Ribosomal protein S19E (S16A) | Linear |
| [J] | COG2260 | Predicted Zn-ribbon RNA-binding protein | Linear |
| [J] | COG2264 | Ribosomal protein L11 methylase | Linear |
| [G] | COG2379 | Putative glycerate kinase | Linear |
| [K] | COG2771 | DNA-binding HTH domain-containing proteins | Linear |
| [R] | COG3573 | Predicted oxidoreductase | Linear |
| [H] | COG3585 | Molybdopterin-binding protein | Linear |
| [R] | COG5141 | PHD zinc finger-containing protein | Linear |
| [T] | COG5212 | Low-affinity cAMP phosphodiesterase | Linear |
| [P] | COG5398 | Heme oxygenase | Linear |
| [O] | COG0450 | Peroxiredoxin | Linear |
| [G] | COG0469 | Pyruvate kinase | Linear |
| [R] | COG0496 | Predicted acid phosphatase | Linear |
| [RTKL] | COG0515 | Serine/threonine protein kinase | Linear |
| [J] | COG0565 | rRNA methylase | Linear |
| [J] | COG0566 | rRNA methylases | Linear |
| [K] | COG0571 | dsRNA-specific ribonuclease | Linear |
| [F] | COG0572 | Uridine kinase | Linear |
| [R] | COG0628 | Predicted permease | Linear |
| [R] | COG0714 | MoxR-like ATPases | Linear |
| [F] | COG0717 | Deoxycytidine deaminase | Linear |
| [J] | COG1997 | Ribosomal protein L37AE/L43A | Linear |
| [J] | COG1998 | Ribosomal protein S27AE | Linear |
| [R] | COG2118 | DNA-binding protein | Linear |
| [J] | COG2139 | Ribosomal protein L21E | Linear |
| [J] | COG2147 | Ribosomal protein L19E | Linear |
| [K] | COG2207 | AraC-type DNA-binding domain-containing proteins | Linear |
| [H] | COG2241 | Precorrin-6B methylase 1 | Linear |
| [H] | COG2242 | Precorrin-6B methylase 2 | Linear |
| [J] | COG2263 | Predicted RNA methylase | Linear |
| [P] | COG3230 | Heme oxygenase | Linear |
| [G] | COG3265 | Gluconate kinase | Linear |
| [S] | COG5561 | Predicted metal-binding protein | Linear |
| [L] | COG0632 | Holliday junction resolvasome, DNA-binding subunit | Linear |
| [E] | COG0665 | Glycine/D-amino acid oxidases (deaminating) | Linear |
| [C] | COG0716 | Flavodoxins | Linear |
| [R] | COG0727 | Predicted Fe-S-cluster oxidoreductase | Linear |
| [M] | COG0771 | UDP-N-acetylmuramoylalanine-D-glutamate ligase | Linear |
| [J] | COG0858 | Ribosome-binding factor A | Linear |
| [L] | COG0863 | DNA modification methylase | Linear |
| [C] | COG1069 | Ribulose kinase | Linear |
| [H] | COG1072 | Panthothenate kinase | Linear |
| [F] | COG1102 | Cytidylate kinase | Linear |
| [R] | COG1745 | Predicted metal-binding protein | Linear |
| [H] | COG2240 | Pyridoxal/pyridoxine/pyridoxamine kinase | Linear |
| [H] | COG2243 | Precorrin-2 methylase | Linear |
| [C] | COG2414 | Aldehyde:ferredoxin oxidoreductase | Linear |
| [J] | COG5051 | Ribosomal protein L36E | Linear |
| [O] | COG0386 | Glutathione peroxidase | Linear |
| [I] | COG0671 | Membrane-associated phospholipid phosphatase | Linear |
| [P] | COG0672 | High-affinity Fe2+/Pb2+ permease | Linear |
| [L] | COG0742 | N6-adenine-specific methylase | Linear |
| [I] | COG2267 | Lysophospholipase | Linear |
| [R] | COG4639 | Predicted kinase | Linear |
| [KL] | COG4646 | DNA methylase | Linear |
| [R] | COG5012 | Predicted cobalamin binding protein | Linear |
| [T] | COG0394 | Protein-tyrosine-phosphatase | Linear |
| [L] | COG0629 | Single-stranded DNA-binding protein | Linear |
| [T] | COG0631 | Serine/threonine protein phosphatase | Linear |
| [P] | COG1785 | Alkaline phosphatase | Linear |
| [R] | COG4989 | Predicted oxidoreductase | Linear |
| [R] | COG4996 | Predicted phosphatase | Linear |
| [R] | COG0637 | Predicted phosphatase/phosphohexomutase | Linear |
| [R] | COG5088 | Rad5p-binding protein | Linear |
| [C] | COG0633 | Ferredoxin | Linear |
| [E] | COG0106 | Phosphoribosylformimino-5-aminoimidazole carboxamide ribonucleotide (ProFAR) isomerase | Metabolism |
| [F] | COG0138 | AICAR transformylase/IMP cyclohydrolase PurH (only IMP cyclohydrolase domain in Aful) | Metabolism |
| [L] | COG0187 | Type IIA topoisomerase (DNA gyrase/topo II, topoisomerase IV), B subunit | Metabolism |
| [L] | COG0188 | Type IIA topoisomerase (DNA gyrase/topo II, topoisomerase IV), A subunit | Metabolism |
| [L] | COG0210 | Superfamily I DNA and RNA helicases | Metabolism |
| [J] | COG1188 | Ribosome-associated heat shock protein implicated in the recycling of the 50S subunit (S4 paralog) | Metabolism |
| [L] | COG2452 | Predicted site-specific integrase-resolvase | Metabolism |
| [H] | COG3172 | Predicted ATPase/kinase involved in NAD metabolism | Metabolism |
| [KE] | COG3283 | Transcriptional regulator of aromatic amino acids metabolism | Metabolism |
| [T] | COG3290 | Signal transduction histidine kinase regulating citrate/malate metabolism | Metabolism |
| [P] | COG3454 | Metal-dependent hydrolase involved in phosphonate metabolism | Metabolism |
| [K] | COG5068 | Regulator of arginine metabolism and related MADS box-containing transcription factors | Metabolism |
| [B] | COG5531 | SWIB-domain-containing proteins implicated in chromatin remodeling | Metabolism |
| [J] | COG0219 | Predicted rRNA methylase (SpoU class) | Metabolism |
| [U] | COG0341 | Preprotein translocase subunit SecF | Metabolism |
| [U] | COG0342 | Preprotein translocase subunit SecD | Metabolism |
| [L] | COG0350 | Methylated DNA-protein cysteine methyltransferase | Metabolism |
| [R] | COG0596 | Predicted hydrolases or acyltransferases (alpha/beta hydrolase superfamily) | Metabolism |
| [O] | COG0652 | Peptidyl-prolyl cis-trans isomerase (rotamase) - cyclophilin family | Metabolism |
| [U] | COG0653 | Preprotein translocase subunit SecA (ATPase, RNA helicase) | Metabolism |
| [B] | COG5034 | Chromatin remodeling protein, contains PhD zinc finger | Metabolism |
| [BK] | COG5259 | RSC chromatin remodeling complex subunit RSC8 | Metabolism |
| [E] | COG5425 | Usg protein, probable subunit of phosphoribosylanthranilate isomerase | Metabolism |
| [G] | COG0235 | Ribulose-5-phosphate 4-epimerase and related epimerases and aldolases | Metabolism |
| [H] | COG0314 | Molybdopterin converting factor, large subunit | Metabolism |
| [OC] | COG0526 | Thiol-disulfide isomerase and thioredoxins | Metabolism |
| [H] | COG1713 | Predicted HD superfamily hydrolase involved in NAD metabolism | Metabolism |
| [R] | COG2150 | Predicted regulator of amino acid metabolism, contains ACT domain | Metabolism |
| [QK] | COG3284 | Transcriptional activator of acetoin/glycerol metabolism | Metabolism |
| [G] | COG3635 | Predicted phosphoglycerate mutase, AP superfamily | Metabolism |
| [O] | COG4070 | Predicted peptidyl-prolyl cis-trans isomerase (rotamase), cyclophilin family | Metabolism |
| [EH] | COG0059 | Ketol-acid reductoisomerase | Metabolism |
| [O] | COG0298 | Hydrogenase maturation factor | Metabolism |
| [J] | COG0324 | tRNA delta(2)-isopentenylpyrophosphate transferase | Metabolism |
| [J] | COG0343 | Queuine/archaeosine tRNA-ribosyltransferase | Metabolism |
| [R] | COG0354 | Predicted aminomethyltransferase related to GcvT | Metabolism |
| [O] | COG0544 | FKBP-type peptidyl-prolyl cis-trans isomerase (trigger factor) | Metabolism |
| [G] | COG0698 | Ribose 5-phosphate isomerase RpiB | Metabolism |
| [G] | COG2513 | PEP phosphonomutase and related enzymes | Metabolism |
| [V] | COG4403 | Lantibiotic modifying enzyme | Metabolism |
| [M] | COG4952 | Predicted sugar isomerase | Metabolism |
| [J] | COG0242 | N-formylmethionyl-tRNA deformylase | Metabolism |
| [E] | COG0253 | Diaminopimelate epimerase | Metabolism |
| [L] | COG0305 | Replicative DNA helicase | Metabolism |
| [I] | COG0331 | (acyl-carrier-protein) S-malonyltransferase | Metabolism |
| [O] | COG0435 | Predicted glutathione S-transferase | Metabolism |
| [O] | COG0545 | FKBP-type peptidyl-prolyl cis-trans isomerases 1 | Metabolism |
| [MG] | COG0702 | Predicted nucleoside-diphosphate-sugar epimerases | Metabolism |
| [L] | COG1389 | DNA topoisomerase VI, subunit B | Metabolism |
| [L] | COG1643 | HrpA-like helicases | Metabolism |
| [O] | COG1651 | Protein-disulfide isomerase | Metabolism |
| [Q] | COG2761 | Predicted dithiol-disulfide isomerase involved in polyketide biosynthesis | Metabolism |
| [G] | COG2942 | N-acyl-D-glucosamine 2-epimerase | Metabolism |
| [G] | COG3010 | Putative N-acetylmannosamine-6-phosphate epimerase | Metabolism |
| [G] | COG3623 | Putative L-xylulose-5-phosphate 3-epimerase | Metabolism |
| [R] | COG3631 | Ketosteroid isomerase-related protein | Metabolism |
| [G] | COG3717 | 5-keto 4-deoxyuronate isomerase | Metabolism |
| [Q] | COG3917 | 2-hydroxychromene-2-carboxylate isomerase | Metabolism |
| [E] | COG4401 | Chorismate mutase | Metabolism |
| [U] | COG5066 | VAMP-associated protein involved in inositol metabolism | Metabolism |
| [G] | COG0149 | Triosephosphate isomerase | Metabolism |
| [J] | COG0336 | tRNA-(guanine-N1)-methyltransferase | Metabolism |
| [E] | COG0436 | Aspartate/tyrosine/aromatic aminotransferase | Metabolism |
| [I] | COG0439 | Biotin carboxylase | Metabolism |
| [F] | COG0540 | Aspartate carbamoyltransferase, catalytic chain | Metabolism |
| [G] | COG0588 | Phosphoglycerate mutase 1 | Metabolism |
| [D] | COG1077 | Actin-like ATPase involved in cell morphogenesis | Metabolism |
| [G] | COG1482 | Phosphomannose isomerase | Metabolism |
| [L] | COG1697 | DNA topoisomerase VI, subunit A | Metabolism |
| [I] | COG1884 | Methylmalonyl-CoA mutase, N-terminal domain/subunit | Metabolism |
| [GR] | COG2140 | Thermophilic glucose-6-phosphate isomerase and related metalloenzymes | Metabolism |
| [G] | COG2160 | L-arabinose isomerase | Metabolism |
| [M] | COG2222 | Predicted phosphosugar isomerases | Metabolism |
| [M] | COG2891 | Cell shape-determining protein | Metabolism |
| [Q] | COG3473 | Maleate cis-trans isomerase | Metabolism |
| [L] | COG3569 | Topoisomerase IB | Metabolism |
| [G] | COG0120 | Ribose 5-phosphate isomerase | Metabolism |
| [G] | COG0148 | Enolase | Metabolism |
| [F] | COG0274 | Deoxyribose-phosphate aldolase | Metabolism |
| [G] | COG0279 | Phosphoheptose isomerase | Metabolism |
| [G] | COG0363 | 6-phosphogluconolactonase/Glucosamine-6-phosphate isomerase/deaminase | Metabolism |
| [L] | COG0389 | Nucleotidyltransferase/DNA polymerase involved in DNA repair | Metabolism |
| [H] | COG0413 | Ketopantoate hydroxymethyltransferase | Metabolism |
| [M] | COG0438 | Glycosyltransferase | Metabolism |
| [L] | COG0550 | Topoisomerase IA | Metabolism |
| [M] | COG0562 | UDP-galactopyranose mutase | Metabolism |
| [MG] | COG1086 | Predicted nucleoside-diphosphate sugar epimerases | Metabolism |
| [R] | COG1090 | Predicted nucleoside-diphosphate sugar epimerase | Metabolism |
| [J] | COG1206 | NAD(FAD)-utilizing enzyme possibly involved in translation | Metabolism |
| [I] | COG1443 | Isopentenyldiphosphate isomerase | Metabolism |
| [G] | COG1621 | Beta-fructosidases (levanase/invertase) | Metabolism |
| [H] | COG2082 | Precorrin isomerase | Metabolism |
| [R] | COG2103 | Predicted sugar phosphate isomerase | Metabolism |
| [G] | COG2407 | L-fucose isomerase and related proteins | Metabolism |
| [G] | COG3622 | Hydroxypyruvate isomerase | Metabolism |
| [Q] | COG3653 | N-acyl-D-aspartate/D-glutamate deacylase | Metabolism |
| [CI] | COG5274 | Cytochrome b involved in lipid metabolism | Metabolism |
| [F] | COG0041 | Phosphoribosylcarboxyaminoimidazole (NCAIR) mutase | Metabolism |
| [G] | COG0166 | Glucose-6-phosphate isomerase | Metabolism |
| [L] | COG0177 | Predicted EndoIII-related endonuclease | Metabolism |
| [G] | COG0191 | Fructose/tagatose bisphosphate aldolase | Metabolism |
| [E] | COG0405 | Gamma-glutamyltransferase | Metabolism |
| [G] | COG0406 | Fructose-2,6-bisphosphatase | Metabolism |
| [MG] | COG0451 | Nucleoside-diphosphate-sugar epimerases | Metabolism |
| [L] | COG0582 | Integrase | Metabolism |
| [R] | COG0612 | Predicted Zn-dependent peptidases | Metabolism |
| [I] | COG0657 | Esterase/lipase | Metabolism |
| [G] | COG1082 | Sugar phosphate isomerases/epimerases | Metabolism |
| [I] | COG2185 | Methylmalonyl-CoA mutase, C-terminal domain/subunit (cobalamin-binding) | Metabolism |
| [G] | COG4101 | Predicted mannose-6-phosphate isomerase | Metabolism |
| [S] | COG4319 | Ketosteroid isomerase homolog | Metabolism |
| [G] | COG4806 | L-rhamnose isomerase | Metabolism |
| [Q] | COG4829 | Muconolactone delta-isomerase | Metabolism |
| [E] | COG0135 | Phosphoribosylanthranilate isomerase | Metabolism |
| [H] | COG0382 | 4-hydroxybenzoate polyprenyltransferase and related prenyltransferases | Metabolism |
| [L] | COG0468 | RecA/RadA recombinase | Metabolism |
| [P] | COG0607 | Rhodanese-related sulfurtransferase | Metabolism |
| [O] | COG0625 | Glutathione S-transferase | Metabolism |
| [R] | COG0627 | Predicted esterase | Metabolism |
| [M] | COG0766 | UDP-N-acetylglucosamine enolpyruvyl transferase | Metabolism |
| [M] | COG1087 | UDP-glucose 4-epimerase | Metabolism |
| [G] | COG2115 | Xylose isomerase | Metabolism |
| [E] | COG3232 | 5-carboxymethyl-2-hydroxymuconate isomerase | Metabolism |
| [O] | COG5207 | Isopeptidase T | Metabolism |
| [G] | COG0033 | Phosphoglucomutase | Metabolism |
| [G] | COG0036 | Pentose-5-phosphate-3-epimerase | Metabolism |
| [R] | COG0384 | Predicted epimerase, PhzC/PhzF homolog | Metabolism |
| [MI] | COG0615 | Cytidylyltransferase | Metabolism |
| [G] | COG1904 | Glucuronate isomerase | Metabolism |
| [M] | COG0381 | UDP-N-acetylglucosamine 2-epimerase | Metabolism |
| [G] | COG0662 | Mannose-6-phosphate isomerase | Metabolism |
| [OK] | COG0378 | Ni2+-binding GTPase involved in regulation of expression and maturation of urease and hydrogenase | Regulation |
| [O] | COG0425 | Predicted redox protein, regulator of disulfide bond formation | Regulation |
| [P] | COG0490 | Putative regulatory, ligand-binding protein related to C-terminal domains of K+ channels | Regulation |
| [M] | COG0741 | Soluble lytic murein transglycosylase and related regulatory proteins (some contain LysM/invasin domains) | Regulation |
| [K] | COG0864 | Predicted transcriptional regulators containing the CopG/Arc/MetJ DNA-binding domain and a metal-binding domain | Regulation |
| [ER] | COG1387 | Histidinol phosphatase and related hydrolases of the PHP family | Regulation |
| [T] | COG1493 | Serine kinase of the HPr protein, regulates carbohydrate metabolism | Regulation |
| [T] | COG1875 | Predicted ATPase related to phosphate starvation-inducible protein PhoH | Regulation |
| [E] | COG2061 | ACT-domain-containing protein, predicted allosteric regulator of homoserine dehydrogenase | Regulation |
| [TK] | COG2197 | Response regulator containing a CheY-like receiver domain and an HTH DNA-binding domain | Regulation |
| [K] | COG2865 | Predicted transcriptional regulator containing an HTH domain and an uncharacterized domain shared with the mammalian protein Schlafen | Regulation |
| [K] | COG3357 | Predicted transcriptional regulator containing an HTH domain fused to a Zn-ribbon | Regulation |
| [KT] | COG3604 | Transcriptional regulator containing GAF, AAA-type ATPase, and DNA binding domains | Regulation |
| [K] | COG3609 | Predicted transcriptional regulators containing the CopG/Arc/MetJ DNA-binding domain | Regulation |
| [KT] | COG3829 | Transcriptional regulator containing PAS, AAA-type ATPase, and DNA-binding domains | Regulation |
| [KT] | COG4219 | Antirepressor regulating drug resistance, predicted signal transduction N-terminal membrane component | Regulation |
| [R] | COG4225 | Predicted unsaturated glucuronyl hydrolase involved in regulation of bacterial surface properties, and related proteins | Regulation |
| [KT] | COG4650 | Sigma54-dependent transcription regulator containing an AAA-type ATPase domain and a DNA-binding domain | Regulation |
| [K] | COG4977 | Transcriptional regulator containing an amidase domain and an AraC-type DNA-binding HTH domain | Regulation |
| [DT] | COG5261 | Protein involved in regulation of cellular morphogenesis/cytokinesis | Regulation |
| [N] | COG5442 | Flagellar biosynthesis regulator FlaF | Regulation |
| [N] | COG5443 | Flagellar biosynthesis regulator FlbT | Regulation |
| [K] | COG5499 | Predicted transcription regulator containing HTH domain | Regulation |
| [K] | COG5631 | Predicted transcription regulator, contains HTH domain (MarR family) | Regulation |
| [K] | COG5662 | Predicted transmembrane transcriptional regulator (anti-sigma factor) | Regulation |
| [K] | COG5665 | CCR4-NOT transcriptional regulation complex, NOT5 subunit | Regulation |
| [K] | COG1327 | Predicted transcriptional regulator, consists of a Zn-ribbon and ATP-cone domains | Regulation |
| [K] | COG1521 | Putative transcriptional regulator, homolog of Bvg accessory factor | Regulation |
| [OU] | COG1585 | Membrane protein implicated in regulation of membrane protease activity | Regulation |
| [K] | COG2524 | Predicted transcriptional regulator, contains C-terminal CBS domains | Regulation |
| [K] | COG4800 | Predicted transcriptional regulator with an HTH domain | Regulation |
| [T] | COG5170 | Serine/threonine protein phosphatase 2A, regulatory subunit | Regulation |
| [K] | COG1386 | Predicted transcriptional regulator containing the HTH domain | Regulation |
| [P] | COG1392 | Phosphate transport regulator (distant homolog of PhoU) | Regulation |
| [P] | COG1393 | Arsenate reductase and related proteins, glutaredoxin family | Regulation |
| [O] | COG1764 | Predicted redox protein, regulator of disulfide bond formation | Regulation |
| [O] | COG1765 | Predicted redox protein, regulator of disulfide bond formation | Regulation |
| [T] | COG2172 | Anti-sigma regulatory factor (Ser/Thr protein kinase) | Regulation |
| [K] | COG3070 | Regulator of competence-specific genes | Regulation |
| [K] | COG3160 | Regulator of sigma D | Regulation |
| [R] | COG3641 | Predicted membrane protein, putative toxin regulator | Regulation |
| [K] | COG3905 | Predicted transcriptional regulator | Regulation |
| [U] | COG4796 | Type II secretory pathway, component HofQ | Regulation |
| [O] | COG4935 | Regulatory P domain of the subtilisin-like proprotein convertases and other proteases | Regulation |
| [KD] | COG5132 | Cell cycle control protein, G10 family | Regulation |
| [K] | COG5625 | Predicted transcription regulator containing HTH domain | Regulation |
| [K] | COG0789 | Predicted transcriptional regulators | Regulation |
| [KG] | COG1349 | Transcriptional regulators of sugar metabolism | Regulation |
| [K] | COG2345 | Predicted transcriptional regulator | Regulation |
| [K] | COG2390 | Transcriptional regulator, contains sigma factor-related N-terminal domain | Regulation |
| [TQ] | COG2508 | Regulator of polyketide synthase expression | Regulation |
| [K] | COG2932 | Predicted transcriptional regulator | Regulation |
| [V] | COG3023 | Negative regulator of beta-lactamase expression | Regulation |
| [KE] | COG3060 | Transcriptional regulator of met regulon | Regulation |
| [K] | COG4957 | Predicted transcriptional regulator | Regulation |
| [O] | COG5110 | 26S proteasome regulatory complex component | Regulation |
| [O] | COG5116 | 26S proteasome regulatory complex component | Regulation |
| [U] | COG5347 | GTPase-activating protein that regulates ARFs (ADP-ribosylation factors), involved in ARF-mediated vesicular transport | Regulation |
| [K] | COG5450 | Transcription regulator of the Arc/MetJ class | Regulation |
| [P] | COG0704 | Phosphate uptake regulator | Regulation |
| [P] | COG0861 | Membrane protein TerC, possibly involved in tellurium resistance | Regulation |
| [K] | COG1329 | Transcriptional regulators, similar to M. xanthus CarD | Regulation |
| [KH] | COG1339 | Transcriptional regulator of a riboflavin/FAD biosynthetic operon | Regulation |
| [K] | COG1475 | Predicted transcriptional regulators | Regulation |
| [K] | COG1476 | Predicted transcriptional regulators | Regulation |
| [K] | COG1959 | Predicted transcriptional regulator | Regulation |
| [E] | COG2716 | Glycine cleavage system regulatory protein | Regulation |
| [D] | COG2846 | Regulator of cell morphogenesis and NO signaling | Regulation |
| [K] | COG2944 | Predicted transcriptional regulator | Regulation |
| [K] | COG3423 | Predicted transcriptional regulator | Regulation |
| [K] | COG3636 | Predicted transcriptional regulator | Regulation |
| [R] | COG3800 | Predicted transcriptional regulator | Regulation |
| [K] | COG4109 | Predicted transcriptional regulator containing CBS domains | Regulation |
| [DKT] | COG5035 | Cell cycle control protein | Regulation |
| [R] | COG0375 | Zn finger protein HypA/HybF (possibly regulating hydrogenase expression) | Regulation |
| [K] | COG1510 | Predicted transcriptional regulators | Regulation |
| [KG] | COG1940 | Transcriptional regulator/sugar kinase | Regulation |
| [K] | COG2378 | Predicted transcriptional regulator | Regulation |
| [R] | COG2522 | Predicted transcriptional regulator | Regulation |
| [K] | COG2808 | Transcriptional regulator | Regulation |
| [K] | COG2909 | ATP-dependent transcriptional regulator | Regulation |
| [R] | COG3054 | Predicted transcriptional regulator | Regulation |
| [S] | COG3619 | Predicted membrane protein | Regulation |
| [K] | COG3655 | Predicted transcriptional regulator | Regulation |
| [K] | COG3722 | Transcriptional regulator | Regulation |
| [KT] | COG3835 | Sugar diacid utilization regulator | Regulation |
| [O] | COG5071 | 26S proteasome regulatory complex component | Regulation |
| [O] | COG5148 | 26S proteasome regulatory complex, subunit RPN10/PSMD4 | Regulation |
| [LK] | COG1197 | Transcription-repair coupling factor (superfamily II helicase) | Regulation |
| [K] | COG1378 | Predicted transcriptional regulators | Regulation |
| [K] | COG1395 | Predicted transcriptional regulator | Regulation |
| [K] | COG1396 | Predicted transcriptional regulators | Regulation |
| [F] | COG1457 | Purine-cytosine permease and related proteins | Regulation |
| [K] | COG1497 | Predicted transcriptional regulator | Regulation |
| [KG] | COG1548 | Predicted transcriptional regulator/sugar kinase | Regulation |
| [TD] | COG1718 | Serine/threonine protein kinase involved in cell cycle control | Regulation |
| [K] | COG1802 | Transcriptional regulators | Regulation |
| [K] | COG2002 | Regulators of stationary/sporulation gene expression | Regulation |
| [K] | COG3682 | Predicted transcriptional regulator | Regulation |
| [E] | COG1168 | Bifunctional PLP-dependent enzyme with beta-cystathionase and maltose regulon repressor activities | Regulation |
| [R] | COG1365 | Predicted ATPase (PP-loop superfamily) | Regulation |
| [K] | COG1522 | Transcriptional regulators | Regulation |
| [K] | COG1695 | Predicted transcriptional regulators | Regulation |
| [K] | COG1709 | Predicted transcriptional regulator | Regulation |
| [K] | COG1777 | Predicted transcriptional regulators | Regulation |
| [K] | COG1846 | Transcriptional regulators | Regulation |
| [KT] | COG1983 | Putative stress-responsive transcriptional regulator | Regulation |
| [T] | COG2336 | Growth regulator | Regulation |
| [S] | COG3157 | Hemolysin-coregulated protein (uncharacterized) | Regulation |
| [J] | COG4937 | Predicted regulatory domain of prephenate dehydrogenase | Regulation |
| [H] | COG5146 | Pantothenate kinase, acetyl-CoA regulated | Regulation |
| [O] | COG5159 | 26S proteasome regulatory complex component | Regulation |
| [DZ] | COG5217 | Microtubule-binding protein involved in cell cycle control | Regulation |
| [P] | COG0735 | Fe2+/Zn2+ uptake regulation proteins | Regulation |
| [K] | COG1609 | Transcriptional regulators | Regulation |
| [K] | COG3311 | Predicted transcriptional regulator | Regulation |
| [K] | COG3355 | Predicted transcriptional regulator | Regulation |
| [K] | COG3432 | Predicted transcriptional regulator | Regulation |
| [K] | COG4189 | Predicted transcriptional regulator | Regulation |
| [K] | COG4190 | Predicted transcriptional regulator | Regulation |
| [K] | COG4738 | Predicted transcriptional regulator | Regulation |
| [K] | COG4742 | Predicted transcriptional regulator | Regulation |
| [KT] | COG4978 | Transcriptional regulator, effector-binding domain/component | Regulation |
| [K] | COG5097 | RNA polymerase II transcriptional regulation mediator | Regulation |
| [O] | COG5187 | 26S proteasome regulatory complex component, contains PCI domain | Regulation |
| [K] | COG1318 | Predicted transcriptional regulators | Regulation |
| [K] | COG1321 | Mn-dependent transcriptional regulator | Regulation |
| [K] | COG1733 | Predicted transcriptional regulators | Regulation |
| [F] | COG1781 | Aspartate carbamoyltransferase, regulatory subunit | Regulation |
| [K] | COG5007 | Predicted transcriptional regulator, BolA superfamily | Regulation |
| [R] | COG0641 | Arylsulfatase regulator (Fe-S oxidoreductase) | Regulation |
| [K] | COG1737 | Transcriptional regulators | Regulation |
| [K] | COG2186 | Transcriptional regulators | Regulation |
| [K] | COG2188 | Transcriptional regulators | Regulation |
| [K] | COG3888 | Predicted transcriptional regulator | Regulation |
| [K] | COG0640 | Predicted transcriptional regulators | Regulation |
| [K] | COG1725 | Predicted transcriptional regulators | Regulation |
| [K] | COG5340 | Predicted transcriptional regulator | Regulation |
| [K] | COG0085 | DNA-directed RNA polymerase, beta subunit/140 kD subunit | Sensing |
| [K] | COG0086 | DNA-directed RNA polymerase, beta' subunit/160 kD subunit | Sensing |
| [K] | COG0202 | DNA-directed RNA polymerase, alpha subunit/40 kD subunit | Sensing |
| [L] | COG0272 | NAD-dependent DNA ligase (contains BRCT domain type II) | Sensing |
| [R] | COG0312 | Predicted Zn-dependent proteases and their inactivated homologs | Sensing |
| [M] | COG0357 | Predicted S-adenosylmethionine-dependent methyltransferase involved in bacterial cell division | Sensing |
| [M] | COG0399 | Predicted pyridoxal phosphate-dependent enzyme apparently involved in regulation of cell wall biogenesis | Sensing |
| [T] | COG0589 | Universal stress protein UspA and related nucleotide-binding proteins | Sensing |
| [ET] | COG0834 | ABC-type amino acid transport/signal transduction systems, periplasmic component/domain | Sensing |
| [L] | COG1074 | ATP-dependent exoDNAse (exonuclease V) beta subunit (contains helicase and exonuclease domains) | Sensing |
| [R] | COG1310 | Predicted metal-dependent protease of the PAD1/JAB1 superfamily | Sensing |
| [K] | COG1508 | DNA-directed RNA polymerase specialized sigma subunit, sigma54 homolog | Sensing |
| [T] | COG1551 | Carbon storage regulator (could also regulate swarming and quorum sensing) | Sensing |
| [R] | COG2159 | Predicted metal-dependent hydrolase of the TIM-barrel fold | Sensing |
| [T] | COG2905 | Predicted signal-transduction protein containing cAMP-binding and CBS domains | Sensing |
| [T] | COG3476 | Tryptophan-rich sensory protein (mitochondrial benzodiazepine receptor homolog) | Sensing |
| [T] | COG3887 | Predicted signaling protein consisting of a modified GGDEF domain and a DHH domain | Sensing |
| [OTK] | COG4512 | Membrane protein putatively involved in post-translational modification of the autoinducing quorum-sensing peptide | Sensing |
| [T] | COG5001 | Predicted signal transduction protein containing a membrane domain, an EAL and a GGDEF domain | Sensing |
| [KT] | COG1842 | Phage shock protein A (IM30), suppresses sigma54-dependent transcription | Sensing |
| [R] | COG2220 | Predicted Zn-dependent hydrolases of the beta-lactamase fold | Sensing |
| [T] | COG3434 | Predicted signal transduction protein containing EAL and modified HD-GYP domains | Sensing |
| [T] | COG3447 | Predicted integral membrane sensor domain | Sensing |
| [T] | COG3452 | Predicted periplasmic ligand-binding sensor domain | Sensing |
| [R] | COG3618 | Predicted metal-dependent hydrolase of the TIM-barrel fold | Sensing |
| [T] | COG3851 | Signal transduction histidine kinase, glucose-6-phosphate specific | Sensing |
| [T] | COG3852 | Signal transduction histidine kinase, nitrogen specific | Sensing |
| [FR] | COG0402 | Cytosine deaminase and related metal-dependent hydrolases | Sensing |
| [L] | COG1311 | Archaeal DNA polymerase II, small subunit/DNA polymerase delta, subunit B | Sensing |
| [T] | COG1639 | Predicted signal transduction protein | Sensing |
| [C] | COG2141 | Coenzyme F420-dependent N5,N10-methylene tetrahydromethanopterin reductase and related flavin-dependent oxidoreductases | Sensing |
| [T] | COG3292 | Predicted periplasmic ligand-binding sensor domain | Sensing |
| [T] | COG3605 | Signal transduction protein containing GAF and PtsI domains | Sensing |
| [T] | COG3850 | Signal transduction histidine kinase, nitrate/nitrite-specific | Sensing |
| [T] | COG3920 | Signal transduction histidine kinase | Sensing |
| [T] | COG5000 | Signal transduction histidine kinase involved in nitrogen fixation and metabolism regulation | Sensing |
| [ER] | COG0493 | NADPH-dependent glutamate synthase beta chain and related oxidoreductases | Sensing |
| [NT] | COG1352 | Methylase of chemotaxis methyl-accepting proteins | Sensing |
| [R] | COG1574 | Predicted metal-dependent hydrolase with the TIM-barrel fold | Sensing |
| [P] | COG2837 | Predicted iron-dependent peroxidase | Sensing |
| [T] | COG3300 | MHYT domain (predicted integral membrane sensor domain) | Sensing |
| [T] | COG3614 | Predicted periplasmic ligand-binding sensor domain | Sensing |
| [PT] | COG3712 | Fe2+-dicitrate sensor, membrane component | Sensing |
| [R] | COG3729 | General stress protein | Sensing |
| [R] | COG4783 | Putative Zn-dependent protease, contains TPR repeats | Sensing |
| [K] | COG4941 | Predicted RNA polymerase sigma factor containing a TPR repeat domain | Sensing |
| [K] | COG5169 | Heat shock transcription factor | Sensing |
| [L] | COG0358 | DNA primase (bacterial type) | Sensing |
| [T] | COG0467 | RecA-superfamily ATPases implicated in signal transduction | Sensing |
| [NT] | COG0835 | Chemotaxis signal transduction protein | Sensing |
| [C] | COG0843 | Heme/copper-type cytochrome/quinol oxidases, subunit 1 | Sensing |
| [K] | COG0846 | NAD-dependent protein deacetylases, SIR2 family | Sensing |
| [R] | COG1099 | Predicted metal-dependent hydrolases with the TIM-barrel fold | Sensing |
| [T] | COG1217 | Predicted membrane GTPase involved in stress response | Sensing |
| [R] | COG1831 | Predicted metal-dependent hydrolase (urease superfamily) | Sensing |
| [C] | COG1927 | Coenzyme F420-dependent N(5),N(10)-methenyltetrahydromethanopterin dehydrogenase | Sensing |
| [J] | COG2123 | RNase PH-related exoribonuclease | Sensing |
| [R] | COG2130 | Putative NADP-dependent oxidoreductases | Sensing |
| [O] | COG3187 | Heat shock protein | Sensing |
| [L] | COG5260 | DNA polymerase sigma | Sensing |
| [L] | COG0084 | Mg-dependent DNase | Sensing |
| [T] | COG0271 | Stress-induced morphogen (activity unknown) | Sensing |
| [U] | COG0681 | Signal peptidase I | Sensing |
| [O] | COG1220 | ATP-dependent protease HslVU (ClpYQ), ATPase subunit | Sensing |
| [R] | COG1473 | Metal-dependent amidase/aminoacylase/carboxypeptidase | Sensing |
| [R] | COG1735 | Predicted metal-dependent hydrolase with the TIM-barrel fold | Sensing |
| [TK] | COG2208 | Serine phosphatase RsbU, regulator of sigma subunit | Sensing |
| [T] | COG4585 | Signal transduction histidine kinase | Sensing |
| [NT] | COG0840 | Methyl-accepting chemotaxis protein | Sensing |
| [O] | COG1219 | ATP-dependent protease Clp, ATPase subunit | Sensing |
| [R] | COG1266 | Predicted metal-dependent membrane protease | Sensing |
| [R] | COG1878 | Predicted metal-dependent hydrolase | Sensing |
| [P] | COG2906 | Bacterioferritin-associated ferredoxin | Sensing |
| [T] | COG3322 | Predicted periplasmic ligand-binding sensor domain | Sensing |
| [R] | COG0491 | Zn-dependent hydrolases, including glyoxylases | Sensing |
| [NT] | COG0643 | Chemotaxis protein histidine kinase and related kinases | Sensing |
| [O] | COG1066 | Predicted ATP-dependent serine protease | Sensing |
| [R] | COG1103 | Archaea-specific pyridoxal phosphate-dependent enzymes | Sensing |
| [P] | COG1218 | 3'-Phosphoadenosine 5'-phosphosulfate (PAPS) 3'-phosphatase | Sensing |
| [K] | COG1278 | Cold shock proteins | Sensing |
| [T] | COG2205 | Osmosensitive K+ channel histidine kinase | Sensing |
| [T] | COG5278 | Predicted periplasmic ligand-binding sensor domain | Sensing |
| [O] | COG1067 | Predicted ATP-dependent protease | Sensing |
| [F] | COG1328 | Oxygen-sensitive ribonucleoside-triphosphate reductase | Sensing |
| [NT] | COG1871 | Chemotaxis protein; stimulates methylation of MCP proteins | Sensing |
| [T] | COG4564 | Signal transduction histidine kinase | Sensing |
| [T] | COG0642 | Signal transduction histidine kinase | Sensing |
| [R] | COG3872 | Predicted metal-dependent enzyme | Sensing |
| [T] | COG4251 | Bacteriophytochrome (light-regulated signal transduction histidine kinase) | Sensing |
| [TK] | COG4936 | Predicted sensor domain | Sensing |
| [T] | COG5002 | Signal transduction histidine kinase | Sensing |
| [R] | COG2321 | Predicted metalloprotease | Sensing |
| [T] | COG4252 | Predicted transmembrane sensor domain | Sensing |
| [T] | COG4250 | Predicted sensor protein/domain | Sensing |
| [C] | COG1838 | Tartrate dehydratase beta subunit/Fumarate hydratase class I, C-terminal domain | Subunit |
| [C] | COG1951 | Tartrate dehydratase alpha subunit/Fumarate hydratase class I, N-terminal domain | Subunit |
| [C] | COG2080 | Aerobic-type carbon monoxide dehydrogenase, small subunit CoxS/CutS homologs | Subunit |
| [K] | COG2101 | TATA-box binding protein (TBP), component of TFIID and TFIIIB | Subunit |
| [JA] | COG2136 | Predicted exosome subunit/U3 small nucleolar ribonucleoprotein (snoRNP) component, contains IMP4 domain | Subunit |
| [C] | COG2221 | Dissimilatory sulfite reductase (desulfoviridin), alpha and beta subunits | Subunit |
| [L] | COG2256 | ATPase related to the helicase subunit of the Holliday junction resolvase | Subunit |
| [R] | COG2517 | Predicted RNA-binding protein containing a C-terminal EMAP domain | Subunit |
| [L] | COG2827 | Predicted endonuclease containing a URI domain | Subunit |
| [S] | COG3978 | Acetolactate synthase (isozyme II), small (regulatory) subunit | Subunit |
| [V] | COG4096 | Type I site-specific restriction-modification system, R (restriction) subunit and related helicases | Subunit |
| [C] | COG4117 | Thiosulfate reductase cytochrome B subunit (membrane anchoring protein) | Subunit |
| [G] | COG4305 | Endoglucanase C-terminal domain/subunit and related proteins | Subunit |
| [D] | COG5244 | Dynactin complex subunit involved in mitotic spindle partitioning in anaphase B | Subunit |
| [U] | COG5308 | Nuclear pore complex subunit | Subunit |
| [U] | COG5325 | t-SNARE complex subunit, syntaxin | Subunit |
| [O] | COG5541 | Vesicle coat complex COPI, zeta subunit | Subunit |
| [R] | COG5643 | Protein containing a metal-binding domain shared with formylmethanofuran dehydrogenase subunit E | Subunit |
| [K] | COG2012 | DNA-directed RNA polymerase, subunit H, RpoH/RPB5 | Subunit |
| [J] | COG2024 | Phenylalanyl-tRNA synthetase alpha subunit (archaeal type) | Subunit |
| [L] | COG2176 | DNA polymerase III, alpha subunit (gram-positive type) | Subunit |
| [L] | COG2927 | DNA polymerase III, chi subunit | Subunit |
| [C] | COG2993 | Cbb3-type cytochrome oxidase, cytochrome c subunit | Subunit |
| [P] | COG3256 | Nitric oxide reductase large subunit | Subunit |
| [L] | COG3747 | Phage terminase, small subunit | Subunit |
| [U] | COG5028 | Vesicle coat complex COPII, subunit SEC24/subunit SFB2/subunit SFB3 | Subunit |
| [L] | COG5235 | Single-stranded DNA-binding replication protein A (RPA), medium (30 kD) subunit | Subunit |
| [K] | COG5250 | RNA polymerase II, fourth largest subunit | Subunit |
| [R] | COG5307 | SEC7 domain proteins | Subunit |
| [L] | COG1933 | Archaeal DNA polymerase II, large subunit | Subunit |
| [C] | COG1969 | Ni,Fe-hydrogenase I cytochrome b subunit | Subunit |
| [L] | COG2812 | DNA polymerase III, gamma/tau subunits | Subunit |
| [R] | COG2940 | Proteins containing SET domain | Subunit |
| [T] | COG3103 | SH3 domain protein | Subunit |
| [O] | COG3278 | Cbb3-type cytochrome oxidase, subunit 1 | Subunit |
| [O] | COG3634 | Alkyl hydroperoxide reductase, large subunit | Subunit |
| [L] | COG3728 | Phage terminase, small subunit | Subunit |
| [E] | COG4302 | Ethanolamine ammonia-lyase, small subunit | Subunit |
| [E] | COG4303 | Ethanolamine ammonia-lyase, large subunit | Subunit |
| [R] | COG4449 | Predicted protease of the Abi (CAAX) family | Subunit |
| [E] | COG4583 | Sarcosine oxidase gamma subunit | Subunit |
| [U] | COG5047 | Vesicle coat complex COPII, subunit SEC23 | Subunit |
| [K] | COG5111 | DNA-directed RNA polymerase III, subunit C34 | Subunit |
| [O] | COG5405 | ATP-dependent protease HslVU (ClpYQ), peptidase subunit | Subunit |
| [KLB] | COG5406 | Nucleosome binding factor SPN, SPT16 subunit | Subunit |
| [U] | COG5538 | Preprotein translocase subunit Sec66 | Subunit |
| [B] | COG5602 | Histone deacetylase complex, SIN3 component | Subunit |
| [C] | COG1941 | Coenzyme F420-reducing hydrogenase, gamma subunit | Subunit |
| [K] | COG1996 | DNA-directed RNA polymerase, subunit RPC10 (contains C4-type Zn-finger) | Subunit |
| [J] | COG2023 | RNase P subunit RPR2 | Subunit |
| [B] | COG2036 | Histones H3 and H4 | Subunit |
| [L] | COG2219 | Eukaryotic-type DNA primase, large subunit | Subunit |
| [R] | COG2402 | Predicted nucleic acid-binding protein, contains PIN domain | Subunit |
| [L] | COG3050 | DNA polymerase III, psi subunit | Subunit |
| [C] | COG3288 | NAD/NADP transhydrogenase alpha subunit | Subunit |
| [P] | COG3303 | Formate-dependent nitrite reductase, periplasmic cytochrome c552 subunit | Subunit |
| [Q] | COG3485 | Protocatechuate 3,4-dioxygenase beta subunit | Subunit |
| [L] | COG3857 | ATP-dependent nuclease, subunit B | Subunit |
| [U] | COG4023 | Preprotein translocase subunit Sec61beta | Subunit |
| [H] | COG4054 | Methyl coenzyme M reductase, beta subunit | Subunit |
| [H] | COG4055 | Methyl coenzyme M reductase, subunit D | Subunit |
| [H] | COG4056 | Methyl coenzyme M reductase, subunit C | Subunit |
| [H] | COG4057 | Methyl coenzyme M reductase, gamma subunit | Subunit |
| [H] | COG4058 | Methyl coenzyme M reductase, alpha subunit | Subunit |
| [F] | COG4630 | Xanthine dehydrogenase, iron-sulfur cluster and FAD-binding subunit A | Subunit |
| [PR] | COG4638 | Phenylpropionate dioxygenase and related ring-hydroxylating dioxygenases, large terminal subunit | Subunit |
| [TDK] | COG5041 | Casein kinase II, beta subunit | Subunit |
| [J] | COG5163 | Protein required for biogenesis of the 60S ribosomal subunit | Subunit |
| [U] | COG5173 | Exocyst complex subunit SEC6 | Subunit |
| [L] | COG5214 | DNA polymerase alpha-primase complex, polymerase-associated subunit B | Subunit |
| [Q] | COG5517 | Small subunit of phenylpropionate dioxygenase | Subunit |
| [R] | COG5529 | Pyocin large subunit | Subunit |
| [G] | COG1850 | Ribulose 1,5-bisphosphate carboxylase, large subunit | Subunit |
| [C] | COG1883 | Na+-transporting methylmalonyl-CoA/oxaloacetate decarboxylase, beta subunit | Subunit |
| [C] | COG2048 | Heterodisulfide reductase, subunit B | Subunit |
| [E] | COG2049 | Allophanate hydrolase subunit 1 | Subunit |
| [C] | COG2180 | Nitrate reductase delta subunit | Subunit |
| [C] | COG2181 | Nitrate reductase gamma subunit | Subunit |
| [C] | COG2218 | Formylmethanofuran dehydrogenase subunit C | Subunit |
| [J] | COG2913 | Small protein A (tmRNA-binding) | Subunit |
| [CO] | COG2924 | Fe-S cluster protector protein | Subunit |
| [C] | COG3043 | Nitrate reductase cytochrome c-type subunit | Subunit |
| [C] | COG3051 | Citrate lyase, alpha subunit | Subunit |
| [C] | COG3052 | Citrate lyase, gamma subunit | Subunit |
| [R] | COG3269 | Predicted RNA-binding protein, contains TRAM domain | Subunit |
| [T] | COG3848 | Phosphohistidine swiveling domain | Subunit |
| [J] | COG4352 | Ribosomal protein L13E | Subunit |
| [R] | COG4624 | Iron only hydrogenase large subunit, C-terminal domain | Subunit |
| [C] | COG4802 | Ferredoxin-thioredoxin reductase, catalytic subunit | Subunit |
| [Q] | COG4909 | Propanediol dehydratase, large subunit | Subunit |
| [Q] | COG4910 | Propanediol dehydratase, small subunit | Subunit |
| [U] | COG5030 | Clathrin adaptor complex, small subunit | Subunit |
| [J] | COG5154 | RNA-binding protein required for 60S ribosomal subunit biogenesis | Subunit |
| [A] | COG5213 | Polyadenylation factor I complex, subunit FIP1 | Subunit |
| [A] | COG5239 | mRNA deadenylase, exonuclease subunit and related nucleases | Subunit |
| [L] | COG5241 | Nucleotide excision repair endonuclease NEF1, RAD10 subunit | Subunit |
| [UR] | COG5391 | Phox homology (PX) domain protein | Subunit |
| [U] | COG5407 | Preprotein translocase subunit Sec63 | Subunit |
| [C] | COG1894 | NADH:ubiquinone oxidoreductase, NADH-binding (51 kD) subunit | Subunit |
| [U] | COG1952 | Preprotein translocase subunit SecB | Subunit |
| [C] | COG3259 | Coenzyme F420-reducing hydrogenase, alpha subunit | Subunit |
| [R] | COG4085 | Predicted RNA-binding protein, contains TRAM domain | Subunit |
| [I] | COG4770 | Acetyl/propionyl-CoA carboxylase, alpha subunit | Subunit |
| [O] | COG5206 | Glycosylphosphatidylinositol transamidase (GPIT), subunit GPI8 | Subunit |
| [U] | COG5240 | Vesicle coat complex COPI, gamma subunit | Subunit |
| [R] | COG5573 | Predicted nucleic-acid-binding protein, contains PIN domain | Subunit |
| [B] | COG5648 | Chromatin-associated proteins containing the HMG domain | Subunit |
| [C] | COG2142 | Succinate dehydrogenase, hydrophobic anchor subunit | Subunit |
| [L] | COG2255 | Holliday junction resolvasome, helicase subunit | Subunit |
| [G] | COG2731 | Beta-galactosidase, beta subunit | Subunit |
| [C] | COG3125 | Heme/copper-type cytochrome/quinol oxidase, subunit 4 | Subunit |
| [C] | COG3260 | Ni,Fe-hydrogenase III small subunit | Subunit |
| [C] | COG3261 | Ni,Fe-hydrogenase III large subunit | Subunit |
| [R] | COG3302 | DMSO reductase anchor subunit | Subunit |
| [K] | COG3343 | DNA-directed RNA polymerase, delta subunit | Subunit |
| [E] | COG4311 | Sarcosine oxidase delta subunit | Subunit |
| [F] | COG4631 | Xanthine dehydrogenase, molybdopterin-binding subunit B | Subunit |
| [O] | COG4736 | Cbb3-type cytochrome oxidase, subunit 3 | Subunit |
| [I] | COG4981 | Enoyl reductase domain of yeast-type FAS1 | Subunit |
| [Z] | COG5022 | Myosin heavy chain | Subunit |
| [U] | COG5074 | t-SNARE complex subunit, syntaxin | Subunit |
| [L] | COG5575 | Origin recognition complex, subunit 2 | Subunit |
| [E] | COG1984 | Allophanate hydrolase subunit 2 | Subunit |
| [G] | COG2301 | Citrate lyase beta subunit | Subunit |
| [R] | COG3953 | SLT domain proteins | Subunit |
| [G] | COG3958 | Transketolase, C-terminal subunit | Subunit |
| [G] | COG3959 | Transketolase, N-terminal subunit | Subunit |
| [C] | COG4231 | Indolepyruvate ferredoxin oxidoreductase, alpha and beta subunits | Subunit |
| [E] | COG4865 | Glutamate mutase epsilon subunit | Subunit |
| [DO] | COG5156 | Anaphase-promoting complex (APC), subunit 10 | Subunit |
| [J] | COG5270 | PUA domain (predicted RNA-binding domain) | Subunit |
| [C] | COG2191 | Formylmethanofuran dehydrogenase subunit E | Subunit |
| [T] | COG2770 | FOG: HAMP domain | Subunit |
| [G] | COG3429 | Glucose-6-P dehydrogenase subunit | Subunit |
| [Q] | COG4647 | Acetone carboxylase, gamma subunit | Subunit |
| [BD] | COG5098 | Chromosome condensation complex Condensin, subunit D2 | Subunit |
| [BD] | COG5218 | Chromosome condensation complex Condensin, subunit G | Subunit |
| [BD] | COG5229 | Chromosome condensation complex Condensin, subunit H | Subunit |
| [C] | COG5231 | Vacuolar H+-ATPase V1 sector, subunit H | Subunit |
| [Z] | COG5245 | Dynein, heavy chain | Subunit |
| [R] | COG5275 | BRCT domain type II | Subunit |
| [R] | COG1783 | Phage terminase large subunit | Subunit |
| [T] | COG2198 | FOG: HPt domain | Subunit |
| [T] | COG2199 | FOG: GGDEF domain | Subunit |
| [T] | COG2200 | FOG: EAL domain | Subunit |
| [T] | COG2202 | FOG: PAS/PAC domain | Subunit |
| [T] | COG2203 | FOG: GAF domain | Subunit |
| [R] | COG4492 | ACT domain-containing protein | Subunit |
| [U] | COG5096 | Vesicle coat complex, various subunits | Subunit |
| [T] | COG2206 | HD-GYP domain | Subunit |
| [U] | COG5232 | Preprotein translocase subunit Sec62 | Subunit |
| [C] | COG2864 | Cytochrome b subunit of formate dehydrogenase | Subunit |
| [A] | COG5228 | mRNA deadenylase subunit | Subunit |
| [N] | COG5603 | Subunit of TRAPP, an ER-Golgi tethering complex | Subunit |
| [I] | COG0332 | 3-oxoacyl-[acyl-carrier-protein] synthase" | Transport_container |
| [J] | COG0012 | Predicted GTPase, probable translation factor | Synthesis |
| [C] | COG0055 | F0F1-type ATP synthase, beta subunit | Synthesis |
| [C] | COG0056 | F0F1-type ATP synthase, alpha subunit | Synthesis |
| [M] | COG0275 | Predicted S-adenosylmethionine-dependent methyltransferase involved in cell envelope biogenesis | Synthesis |
| [H] | COG0311 | Predicted glutamine amidotransferase involved in pyridoxine biosynthesis | Synthesis |
| [L] | COG0323 | DNA mismatch repair enzyme (predicted ATPase) | Synthesis |
| [C] | COG0355 | F0F1-type ATP synthase, epsilon subunit (mitochondrial delta subunit) | Synthesis |
| [M] | COG0449 | Glucosamine 6-phosphate synthetase, contains amidotransferase and phosphosugar isomerase domains | Synthesis |
| [H] | COG0476 | Dinucleotide-utilizing enzymes involved in molybdopterin and thiamine biosynthesis family 2 | Synthesis |
| [L] | COG0556 | Helicase subunit of the DNA excision repair complex | Synthesis |
| [HR] | COG1060 | Thiamine biosynthesis enzyme ThiH and related uncharacterized enzymes | Synthesis |
| [MJ] | COG1208 | Nucleoside-diphosphate-sugar pyrophosphorylase involved in lipopolysaccharide biosynthesis/translation initiation factor 2B, gamma/epsilon subunits (eIF-2Bgamma/eIF-2Bepsilon) | Synthesis |
| [R] | COG1350 | Predicted alternative tryptophan synthase beta-subunit (paralog of TrpB) | Synthesis |
| [C] | COG1456 | CO dehydrogenase/acetyl-CoA synthase gamma subunit (corrinoid Fe-S protein) | Synthesis |
| [R] | COG2329 | Conserved protein involved in polyketide biosynthesis related to monooxygenase | Synthesis |
| [Q] | COG3319 | Thioesterase domains of type I polyketide synthases or non-ribosomal peptide synthetases | Synthesis |
| [Q] | COG3320 | Putative dehydrogenase domain of multifunctional non-ribosomal peptide synthetases and related enzymes | Synthesis |
| [R] | COG3491 | Isopenicillin N synthase and related dioxygenases | Synthesis |
| [L] | COG3723 | Recombinational DNA repair protein (RecE pathway) | Synthesis |
| [G] | COG3936 | Protein involved in polysaccharide intercellular adhesin (PIA) synthesis/biofilm formation | Synthesis |
| [R] | COG4262 | Predicted spermidine synthase with an N-terminal membrane domain | Synthesis |
| [L] | COG4335 | DNA alkylation repair enzyme | Synthesis |
| [Q] | COG4542 | Protein involved in propanediol utilization, and related proteins (includes coumermycin biosynthetic protein), possible kinase | Synthesis |
| [J] | COG5593 | Nucleic-acid-binding protein possibly involved in ribosomal biogenesis | Synthesis |
| [J] | COG0008 | Glutamyl- and glutaminyl-tRNA synthetases | Synthesis |
| [L] | COG0249 | Mismatch repair ATPase (MutS family) | Synthesis |
| [H] | COG0294 | Dihydropteroate synthase and related enzymes | Synthesis |
| [EF] | COG0458 | Carbamoylphosphate synthase large subunit (split gene in MJ) | Synthesis |
| [C] | COG0712 | F0F1-type ATP synthase, delta subunit (mitochondrial oligomycin sensitivity protein) | Synthesis |
| [Q] | COG1020 | Non-ribosomal peptide synthetase modules and related proteins | Synthesis |
| [H] | COG1477 | Membrane-associated lipoprotein involved in thiamine biosynthesis | Synthesis |
| [H] | COG1635 | Flavoprotein involved in thiazole biosynthesis | Synthesis |
| [H] | COG1648 | Siroheme synthase (precorrin-2 oxidase/ferrochelatase domain) | Synthesis |
| [O] | COG5054 | Mitochondrial sulfhydryl oxidase involved in the biogenesis of cytosolic Fe/S proteins | Synthesis |
| [H] | COG5424 | Pyrroloquinoline quinone (Coenzyme PQQ) biosynthesis protein C | Synthesis |
| [J] | COG0048 | Ribosomal protein S12 | Synthesis |
| [J] | COG0049 | Ribosomal protein S7 | Synthesis |
| [J] | COG0051 | Ribosomal protein S10 | Synthesis |
| [J] | COG0052 | Ribosomal protein S2 | Synthesis |
| [H] | COG0054 | Riboflavin synthase beta-chain | Synthesis |
| [E] | COG0067 | Glutamate synthase domain 1 | Synthesis |
| [E] | COG0069 | Glutamate synthase domain 2 | Synthesis |
| [E] | COG0070 | Glutamate synthase domain 3 | Synthesis |
| [J] | COG0072 | Phenylalanyl-tRNA synthetase beta subunit | Synthesis |
| [C] | COG0074 | Succinyl-CoA synthetase, alpha subunit | Synthesis |
| [J] | COG0096 | Ribosomal protein S8 | Synthesis |
| [J] | COG0097 | Ribosomal protein L6P/L9E | Synthesis |
| [J] | COG0098 | Ribosomal protein S5 | Synthesis |
| [J] | COG0099 | Ribosomal protein S13 | Synthesis |
| [J] | COG0100 | Ribosomal protein S11 | Synthesis |
| [J] | COG0102 | Ribosomal protein L13 | Synthesis |
| [J] | COG0103 | Ribosomal protein S9 | Synthesis |
| [H] | COG0108 | 3,4-dihydroxy-2-butanone 4-phosphate synthase | Synthesis |
| [H] | COG0156 | 7-keto-8-aminopelargonate synthetase and related enzymes | Synthesis |
| [IQ] | COG0318 | Acyl-CoA synthetases (AMP-forming)/AMP-acid ligases II | Synthesis |
| [H] | COG0352 | Thiamine monophosphate synthase | Synthesis |
| [E] | COG0440 | Acetolactate synthase, small (regulatory) subunit | Synthesis |
| [F] | COG0519 | GMP synthase, PP-ATPase domain/subunit | Synthesis |
| [E] | COG0646 | Methionine synthase I (cobalamin-dependent), methyltransferase domain | Synthesis |
| [O] | COG0785 | Cytochrome c biogenesis protein | Synthesis |
| [NU] | COG1298 | Flagellar biosynthesis pathway, component FlhA | Synthesis |
| [T] | COG1854 | LuxS protein involved in autoinducer AI2 synthesis | Synthesis |
| [R] | COG2151 | Predicted metal-sulfur cluster biosynthetic enzyme | Synthesis |
| [H] | COG2941 | Ubiquinone biosynthesis protein COQ7 | Synthesis |
| [L] | COG3066 | DNA mismatch repair protein | Synthesis |
| [H] | COG3161 | 4-hydroxybenzoate synthetase (chorismate lyase) | Synthesis |
| [Q] | COG3208 | Predicted thioesterase involved in non-ribosomal peptide biosynthesis | Synthesis |
| [M] | COG3754 | Lipopolysaccharide biosynthesis protein | Synthesis |
| [G] | COG4282 | Protein involved in beta-1,3-glucan synthesis | Synthesis |
| [L] | COG4294 | UV damage repair endonuclease | Synthesis |
| [PE] | COG4362 | Nitric oxide synthase, oxygenase domain | Synthesis |
| [R] | COG4851 | Protein involved in sex pheromone biosynthesis | Synthesis |
| [Q] | COG5310 | Homospermidine synthase | Synthesis |
| [L] | COG5535 | DNA repair protein RAD4 | Synthesis |
| [J] | COG0013 | Alanyl-tRNA synthetase | Synthesis |
| [J] | COG0060 | Isoleucyl-tRNA synthetase | Synthesis |
| [J] | COG0101 | Pseudouridylate synthase | Synthesis |
| [E] | COG0107 | Imidazoleglycerol-phosphate synthase | Synthesis |
| [EH] | COG0147 | Anthranilate/para-aminobenzoate synthases component I | Synthesis |
| [E] | COG0159 | Tryptophan synthase alpha chain | Synthesis |
| [H] | COG0214 | Pyridoxine biosynthesis enzyme | Synthesis |
| [I] | COG0245 | 2C-methyl-D-erythritol 2,4-cyclodiphosphate synthase | Synthesis |
| [H] | COG0303 | Molybdopterin biosynthesis enzyme | Synthesis |
| [H] | COG0307 | Riboflavin synthase alpha chain | Synthesis |
| [H] | COG0315 | Molybdenum cofactor biosynthesis enzyme | Synthesis |
| [EM] | COG0329 | Dihydrodipicolinate synthase/N-acetylneuraminate lyase | Synthesis |
| [E] | COG0337 | 3-dehydroquinate synthetase | Synthesis |
| [C] | COG0356 | F0F1-type ATP synthase, subunit a | Synthesis |
| [I] | COG0365 | Acyl-coenzyme A synthetases/AMP-(fatty) acid ligases | Synthesis |
| [L] | COG0419 | ATPase involved in DNA repair | Synthesis |
| [M] | COG0463 | Glycosyltransferases involved in cell wall biogenesis | Synthesis |
| [H] | COG0521 | Molybdopterin biosynthesis enzymes | Synthesis |
| [E] | COG1410 | Methionine synthase I, cobalamin-binding domain | Synthesis |
| [H] | COG1763 | Molybdopterin-guanine dinucleotide biosynthesis protein | Synthesis |
| [F] | COG1828 | Phosphoribosylformylglycinamidine (FGAM) synthase, PurS component | Synthesis |
| [H] | COG2104 | Sulfur transfer protein involved in thiamine biosynthesis | Synthesis |
| [H] | COG2226 | Methylase involved in ubiquinone/menaquinone biosynthesis | Synthesis |
| [M] | COG2877 | 3-deoxy-D-manno-octulosonic acid (KDO) 8-phosphate synthase | Synthesis |
| [P] | COG3131 | Periplasmic glucans biosynthesis protein | Synthesis |
| [TQ] | COG3916 | N-acyl-L-homoserine lactone synthetase | Synthesis |
| [GM] | COG4464 | Capsular polysaccharide biosynthesis protein | Synthesis |
| [H] | COG4822 | Cobalamin biosynthesis protein CbiK, Co2+ chelatase | Synthesis |
| [J] | COG0080 | Ribosomal protein L11 | Synthesis |
| [J] | COG0081 | Ribosomal protein L1 | Synthesis |
| [J] | COG0087 | Ribosomal protein L3 | Synthesis |
| [J] | COG0088 | Ribosomal protein L4 | Synthesis |
| [J] | COG0089 | Ribosomal protein L23 | Synthesis |
| [J] | COG0090 | Ribosomal protein L2 | Synthesis |
| [J] | COG0091 | Ribosomal protein L22 | Synthesis |
| [J] | COG0092 | Ribosomal protein S3 | Synthesis |
| [J] | COG0093 | Ribosomal protein L14 | Synthesis |
| [J] | COG0094 | Ribosomal protein L5 | Synthesis |
| [H] | COG0095 | Lipoate-protein ligase A | Synthesis |
| [F] | COG0150 | Phosphoribosylaminoimidazole (AIR) synthetase | Synthesis |
| [F] | COG0152 | Phosphoribosylaminoimidazolesuccinocarboxamide (SAICAR) synthase | Synthesis |
| [J] | COG0215 | Cysteinyl-tRNA synthetase | Synthesis |
| [C] | COG0224 | F0F1-type ATP synthase, gamma subunit | Synthesis |
| [G] | COG0269 | 3-hexulose-6-phosphate synthase and related proteins | Synthesis |
| [G] | COG0297 | Glycogen synthase | Synthesis |
| [IQ] | COG0304 | 3-oxoacyl-(acyl-carrier-protein) synthase | Synthesis |
| [TK] | COG0317 | Guanosine polyphosphate pyrophosphohydrolases/synthetases | Synthesis |
| [I] | COG0416 | Fatty acid/phospholipid biosynthesis enzyme | Synthesis |
| [H] | COG0422 | Thiamine biosynthesis protein ThiC | Synthesis |
| [J] | COG0423 | Glycyl-tRNA synthetase (class II) | Synthesis |
| [H] | COG0447 | Dihydroxynaphthoic acid synthase | Synthesis |
| [O] | COG0464 | ATPases of the AAA+ class | Synthesis |
| [M] | COG0510 | Predicted choline kinase involved in LPS biosynthesis | Synthesis |
| [J] | COG0525 | Valyl-tRNA synthetase | Synthesis |
| [I] | COG0821 | Enzyme involved in the deoxyxylulose pathway of isoprenoid biosynthesis | Synthesis |
| [I] | COG1022 | Long-chain acyl-CoA synthetases (AMP-forming) | Synthesis |
| [C] | COG1153 | Formylmethanofuran dehydrogenase subunit D | Synthesis |
| [C] | COG1155 | Archaeal/vacuolar-type H+-ATPase subunit A | Synthesis |
| [C] | COG1271 | Cytochrome bd-type quinol oxidase, subunit 1 | Synthesis |
| [O] | COG1333 | ResB protein required for cytochrome c biosynthesis | Synthesis |
| [F] | COG1351 | Predicted alternative thymidylate synthase | Synthesis |
| [L] | COG1484 | DNA replication protein | Synthesis |
| [I] | COG1502 | Phosphatidylserine/phosphatidylglycerophosphate/cardiolipin synthases and related enzymes | Synthesis |
| [H] | COG1797 | Cobyrinic acid a,c-diamide synthase | Synthesis |
| [C] | COG1880 | CO dehydrogenase/acetyl-CoA synthase epsilon subunit | Synthesis |
| [M] | COG1922 | Teichoic acid biosynthesis proteins | Synthesis |
| [H] | COG2073 | Cobalamin biosynthesis protein CbiG | Synthesis |
| [KNU] | COG2747 | Negative regulator of flagellin synthesis (anti-sigma28 factor) | Synthesis |
| [M] | COG2843 | Putative enzyme of poly-gamma-glutamate biosynthesis (capsule formation) | Synthesis |
| [E] | COG2876 | 3-deoxy-D-arabino-heptulosonate 7-phosphate (DAHP) synthase | Synthesis |
| [H] | COG2896 | Molybdenum cofactor biosynthesis enzyme | Synthesis |
| [I] | COG3243 | Poly(3-hydroxyalkanoate) synthetase | Synthesis |
| [G] | COG3280 | Maltooligosyl trehalose synthase | Synthesis |
| [M] | COG3475 | LPS biosynthesis protein | Synthesis |
| [G] | COG4632 | Exopolysaccharide biosynthesis protein related to N-acetylglucosamine-1-phosphodiester alpha-N-acetylglucosaminidase | Synthesis |
| [L] | COG5627 | DNA repair protein MMS21 | Synthesis |
| [F] | COG0046 | Phosphoribosylformylglycinamidine (FGAM) synthase, synthetase domain | Synthesis |
| [E] | COG0082 | Chorismate synthase | Synthesis |
| [H] | COG0192 | S-adenosylmethionine synthetase | Synthesis |
| [H] | COG0196 | FAD synthase | Synthesis |
| [F] | COG0207 | Thymidylate synthase | Synthesis |
| [H] | COG0320 | Lipoate synthase | Synthesis |
| [E] | COG0367 | Asparagine synthase (glutamine-hydrolyzing) | Synthesis |
| [J] | COG0441 | Threonyl-tRNA synthetase | Synthesis |
| [J] | COG0442 | Prolyl-tRNA synthetase | Synthesis |
| [L] | COG0470 | ATPase involved in DNA replication | Synthesis |
| [E] | COG0709 | Selenophosphate synthase | Synthesis |
| [C] | COG0711 | F0F1-type ATP synthase, subunit b | Synthesis |
| [H] | COG0854 | Pyridoxal phosphate biosynthesis protein | Synthesis |
| [C] | COG1042 | Acyl-CoA synthetase (NDP forming) | Synthesis |
| [J] | COG1187 | 16S rRNA uridine-516 pseudouridylate synthase and related pseudouridylate synthases | Synthesis |
| [J] | COG1384 | Lysyl-tRNA synthetase (class I) | Synthesis |
| [C] | COG1614 | CO dehydrogenase/acetyl-CoA synthase beta subunit | Synthesis |
| [NU] | COG1684 | Flagellar biosynthesis pathway, component FliR | Synthesis |
| [E] | COG1812 | Archaeal S-adenosylmethionine synthetase | Synthesis |
| [R] | COG2516 | Biotin synthase-related enzyme | Synthesis |
| [L] | COG2965 | Primosomal replication protein N | Synthesis |
| [E] | COG3200 | 3-deoxy-D-arabino-heptulosonate 7-phosphate (DAHP) synthase | Synthesis |
| [Q] | COG3321 | Polyketide synthase modules and related proteins | Synthesis |
| [M] | COG3944 | Capsular polysaccharide biosynthesis protein | Synthesis |
| [Q] | COG4264 | Siderophore synthetase component | Synthesis |
| [Q] | COG4693 | Oxidoreductase (NAD-binding), involved in siderophore biosynthesis | Synthesis |
| [GM] | COG5039 | Exopolysaccharide biosynthesis protein | Synthesis |
| [M] | COG5653 | Protein involved in cellulose biosynthesis (CelD) | Synthesis |
| [C] | COG0045 | Succinyl-CoA synthetase, beta subunit | Synthesis |
| [E] | COG0133 | Tryptophan synthase beta chain | Synthesis |
| [H] | COG0285 | Folylpolyglutamate synthase | Synthesis |
| [H] | COG0368 | Cobalamin-5-phosphate synthase | Synthesis |
| [H] | COG0414 | Panthothenate synthetase | Synthesis |
| [E] | COG0421 | Spermidine synthase | Synthesis |
| [L] | COG0497 | ATPase involved in DNA repair | Synthesis |
| [H] | COG0502 | Biotin synthase and related enzymes | Synthesis |
| [I] | COG0558 | Phosphatidylglycerophosphate synthase | Synthesis |
| [J] | COG0564 | Pseudouridylate synthases, 23S RNA-specific | Synthesis |
| [E] | COG0620 | Methionine synthase II (cobalamin-independent) | Synthesis |
| [C] | COG0636 | F0F1-type ATP synthase, subunit c/Archaeal/vacuolar-type H+-ATPase, subunit K | Synthesis |
| [E] | COG0722 | 3-deoxy-D-arabino-heptulosonate 7-phosphate (DAHP) synthase | Synthesis |
| [H] | COG1270 | Cobalamin biosynthesis protein CobD/CbiB | Synthesis |
| [NU] | COG1338 | Flagellar biosynthesis pathway, component FliP | Synthesis |
| [H] | COG1429 | Cobalamin biosynthesis protein CobN and related Mg-chelatases | Synthesis |
| [H] | COG1441 | O-succinylbenzoate synthase | Synthesis |
| [H] | COG1492 | Cobyric acid synthase | Synthesis |
| [G] | COG1803 | Methylglyoxal synthase | Synthesis |
| [M] | COG2089 | Sialic acid synthase | Synthesis |
| [C] | COG2225 | Malate synthase | Synthesis |
| [Q] | COG3207 | Pyoverdine/dityrosine biosynthesis protein | Synthesis |
| [C] | COG3312 | F0F1-type ATP synthase, subunit I | Synthesis |
| [Q] | COG3315 | O-Methyltransferase involved in polyketide biosynthesis | Synthesis |
| [H] | COG4547 | Cobalamin biosynthesis protein CobT (nicotinate-mononucleotide:5, 6-dimethylbenzimidazole phosphoribosyltransferase) | Synthesis |
| [Q] | COG5285 | Protein involved in biosynthesis of mitomycin antibiotics/polyketide fumonisin | Synthesis |
| [E] | COG5630 | Acetylglutamate synthase | Synthesis |
| [J] | COG0016 | Phenylalanyl-tRNA synthetase alpha subunit | Synthesis |
| [E] | COG0119 | Isopropylmalate/homocitrate/citramalate synthases | Synthesis |
| [J] | COG0124 | Histidyl-tRNA synthetase | Synthesis |
| [E] | COG0134 | Indole-3-glycerol phosphate synthase | Synthesis |
| [H] | COG0142 | Geranylgeranyl pyrophosphate synthase | Synthesis |
| [J] | COG0162 | Tyrosyl-tRNA synthetase | Synthesis |
| [H] | COG0171 | NAD synthase | Synthesis |
| [J] | COG0172 | Seryl-tRNA synthetase | Synthesis |
| [J] | COG0173 | Aspartyl-tRNA synthetase | Synthesis |
| [E] | COG0174 | Glutamine synthetase | Synthesis |
| [H] | COG0452 | Phosphopantothenoylcysteine synthetase/decarboxylase | Synthesis |
| [FE] | COG0462 | Phosphoribosylpyrophosphate synthetase | Synthesis |
| [F] | COG0504 | CTP synthase (UTP-ammonia lyase) | Synthesis |
| [EF] | COG0505 | Carbamoylphosphate synthase small subunit | Synthesis |
| [EH] | COG0512 | Anthranilate/para-aminobenzoate synthases component II | Synthesis |
| [E] | COG0626 | Cystathionine beta-lyases/cystathionine gamma-synthases | Synthesis |
| [M] | COG1083 | CMP-N-acetylneuraminic acid synthetase | Synthesis |
| [H] | COG1179 | Dinucleotide-utilizing enzymes involved in molybdopterin and thiamine biosynthesis family 1 | Synthesis |
| [I] | COG1260 | Myo-inositol-1-phosphate synthase | Synthesis |
| [E] | COG1364 | N-acetylglutamate synthase (N-acetylornithine aminotransferase) | Synthesis |
| [E] | COG1465 | Predicted alternative 3-dehydroquinate synthase | Synthesis |
| [H] | COG1541 | Coenzyme F390 synthetase | Synthesis |
| [H] | COG3572 | Gamma-glutamylcysteine synthetase | Synthesis |
| [Q] | COG3882 | Predicted enzyme involved in methoxymalonyl-ACP biosynthesis | Synthesis |
| [M] | COG3955 | Exopolysaccharide biosynthesis protein | Synthesis |
| [L] | COG5145 | DNA excision repair protein | Synthesis |
| [I] | COG0020 | Undecaprenyl pyrophosphate synthase | Synthesis |
| [E] | COG0031 | Cysteine synthase | Synthesis |
| [J] | COG0042 | tRNA-dihydrouridine synthase | Synthesis |
| [E] | COG0128 | 5-enolpyruvylshikimate-3-phosphate synthase | Synthesis |
| [J] | COG0130 | Pseudouridine synthase | Synthesis |
| [H] | COG0132 | Dethiobiotin synthetase | Synthesis |
| [E] | COG0137 | Argininosuccinate synthase | Synthesis |
| [J] | COG0143 | Methionyl-tRNA synthetase | Synthesis |
| [J] | COG0180 | Tryptophanyl-tRNA synthetase | Synthesis |
| [I] | COG0575 | CDP-diglyceride synthetase | Synthesis |
| [J] | COG0621 | 2-methylthioadenine synthetase | Synthesis |
| [H] | COG0720 | 6-pyruvoyl-tetrahydropterin synthase | Synthesis |
| [H] | COG0746 | Molybdopterin-guanine dinucleotide biosynthesis protein A | Synthesis |
| [M] | COG0769 | UDP-N-acetylmuramyl tripeptide synthase | Synthesis |
| [M] | COG0770 | UDP-N-acetylmuramyl pentapeptide synthase | Synthesis |
| [I] | COG1562 | Phytoene/squalene synthetase | Synthesis |
| [J] | COG1736 | Diphthamide synthase subunit DPH2 | Synthesis |
| [H] | COG1767 | Triphosphoribosyl-dephospho-CoA synthetase | Synthesis |
| [L] | COG2003 | DNA repair proteins | Synthesis |
| [R] | COG2144 | Selenophosphate synthetase-related proteins | Synthesis |
| [R] | COG2872 | Predicted metal-dependent hydrolases related to alanyl-tRNA synthetase HxxxH domain | Synthesis |
| [L] | COG3344 | Retron-type reverse transcriptase | Synthesis |
| [Q] | COG3424 | Predicted naringenin-chalcone synthase | Synthesis |
| [I] | COG3425 | 3-hydroxy-3-methylglutaryl CoA synthase | Synthesis |
| [J] | COG0017 | Aspartyl/asparaginyl-tRNA synthetases | Synthesis |
| [J] | COG0018 | Arginyl-tRNA synthetase | Synthesis |
| [J] | COG0495 | Leucyl-tRNA synthetase | Synthesis |
| [E] | COG0498 | Threonine synthase | Synthesis |
| [J] | COG0751 | Glycyl-tRNA synthetase, beta subunit | Synthesis |
| [J] | COG0752 | Glycyl-tRNA synthetase, alpha subunit | Synthesis |
| [R] | COG0820 | Predicted Fe-S-cluster redox enzyme | Synthesis |
| [O] | COG1899 | Deoxyhypusine synthase | Synthesis |
| [G] | COG2273 | Beta-glucanase/Beta-glucan synthetase | Synthesis |
| [F] | COG2759 | Formyltetrahydrofolate synthetase | Synthesis |
| [J] | COG5192 | GTP-binding protein required for 40S ribosome biogenesis | Synthesis |
| [J] | COG1190 | Lysyl-tRNA synthetase (class II) | Synthesis |
| [C] | COG0372 | Citrate synthase | Synthesis |
| [H] | COG0379 | Quinolinate synthase | Synthesis |
| [G] | COG0380 | Trehalose-6-phosphate synthase | Synthesis |
| [E] | COG0754 | Glutathionylspermidine synthase | Synthesis |
| [H] | COG1424 | Pimeloyl-CoA synthetase | Synthesis |
| [H] | COG1587 | Uroporphyrinogen-III synthase | Synthesis |
| [H] | COG1731 | Archaeal riboflavin synthase | Synthesis |
| [I] | COG1183 | Phosphatidylserine synthase | Synthesis |
| [E] | COG1166 | Arginine decarboxylase (spermidine biosynthesis) | Synthesis |
| [E] | COG3962 | Acetolactate synthase | Synthesis |
| [H] | COG1165 | 2-succinyl-6-hydroxy-2,4-cyclohexadiene-1-carboxylate synthase | Synthesis |
| [HQ] | COG1169 | Isochorismate synthase | Synthesis |
| [J] | COG0064 | Asp-tRNAAsn/Glu-tRNAGln amidotransferase B subunit (PET112 homolog) | Transport_container |
| [E] | COG0079 | Histidinol-phosphate/aromatic aminotransferase and cobyric acid decarboxylase | Transport_container |
| [J] | COG0154 | Asp-tRNAAsn/Glu-tRNAGln amidotransferase A subunit and related amidases | Transport_container |
| [EH] | COG0175 | 3'-phosphoadenosine 5'-phosphosulfate sulfotransferase (PAPS reductase)/FAD synthetase and related enzymes | Transport_container |
| [E] | COG0346 | Lactoylglutathione lyase and related lyases | Transport_container |
| [E] | COG0410 | ABC-type branched-chain amino acid transport systems, ATPase component | Transport_container |
| [E] | COG0411 | ABC-type branched-chain amino acid transport systems, ATPase component | Transport_container |
| [E] | COG0559 | Branched-chain amino acid ABC-type transport system, permease components | Transport_container |
| [E] | COG0683 | ABC-type branched-chain amino acid transport systems, periplasmic component | Transport_container |
| [O] | COG0719 | ABC-type transport system involved in Fe-S cluster assembly, permease component | Transport_container |
| [Q] | COG0767 | ABC-type transport system involved in resistance to organic solvents, permease component | Transport_container |
| [P] | COG0803 | ABC-type metal ion transport system, periplasmic component/surface adhesin | Transport_container |
| [Q] | COG1127 | ABC-type transport system involved in resistance to organic solvents, ATPase component | Transport_container |
| [I] | COG1133 | ABC-type long-chain fatty acid transport system, fused permease and ATPase components | Transport_container |
| [KE] | COG1167 | Transcriptional regulators containing a DNA-binding HTH domain and an aminotransferase domain (MocR family) and their eukaryotic orthologs | Transport_container |
| [R] | COG1277 | ABC-type transport system involved in multi-copper enzyme maturation, permease component | Transport_container |
| [Q] | COG1463 | ABC-type transport system involved in resistance to organic solvents, periplasmic component | Transport_container |
| [G] | COG1638 | TRAP-type C4-dicarboxylate transport system, periplasmic component | Transport_container |
| [M] | COG1732 | Periplasmic glycine betaine/choline-binding (lipo)protein of an ABC-type transport system (osmoprotectant binding protein) | Transport_container |
| [M] | COG1807 | 4-amino-4-deoxy-L-arabinose transferase and related glycosyltransferases of PMT family | Transport_container |
| [M] | COG1887 | Putative glycosyl/glycerophosphate transferases involved in teichoic acid biosynthesis TagF/TagB/EpsJ/RodC | Transport_container |
| [P] | COG1914 | Mn2+ and Fe2+ transporters of the NRAMP family | Transport_container |
| [P] | COG1965 | Protein implicated in iron transport, frataxin homolog | Transport_container |
| [P] | COG2011 | ABC-type metal ion transport system, permease component | Transport_container |
| [J] | COG2117 | Predicted subunit of tRNA(5-methylaminomethyl-2-thiouridylate) methyltransferase, contains the PP-loop ATPase domain | Transport_container |
| [R] | COG2244 | Membrane protein involved in the export of O-antigen and teichoic acid | Transport_container |
| [R] | COG2358 | TRAP-type uncharacterized transport system, periplasmic component | Transport_container |
| [O] | COG2386 | ABC-type transport system involved in cytochrome c biogenesis, permease component | Transport_container |
| [J] | COG2511 | Archaeal Glu-tRNAGln amidotransferase subunit E (contains GAD domain) | Transport_container |
| [P] | COG2822 | Predicted periplasmic lipoprotein involved in iron transport | Transport_container |
| [Q] | COG2854 | ABC-type transport system involved in resistance to organic solvents, auxiliary component | Transport_container |
| [R] | COG3030 | Protein affecting phage T7 exclusion by the F plasmid | Transport_container |
| [T] | COG3109 | Activator of osmoprotectant transporter ProP | Transport_container |
| [Q] | COG3127 | Predicted ABC-type transport system involved in lysophospholipase L1 biosynthesis, permease component | Transport_container |
| [R] | COG3178 | Predicted phosphotransferase related to Ser/Thr protein kinases | Transport_container |
| [N] | COG3225 | ABC-type uncharacterized transport system involved in gliding motility, auxiliary component | Transport_container |
| [P] | COG3263 | NhaP-type Na+/H+ and K+/H+ antiporters with a unique C-terminal domain | Transport_container |
| [O] | COG3914 | Predicted O-linked N-acetylglucosamine transferase, SPINDLY family | Transport_container |
| [G] | COG3925 | N-terminal domain of the phosphotransferase system fructose-specific component IIB | Transport_container |
| [R] | COG3956 | Protein containing tetrapyrrole methyltransferase domain and MazG-like (predicted pyrophosphatase) domain | Transport_container |
| [M] | COG3967 | Short-chain dehydrogenase involved in D-alanine esterification of lipoteichoic acid and wall teichoic acid (D-alanine transfer protein) | Transport_container |
| [M] | COG3980 | Spore coat polysaccharide biosynthesis protein, predicted glycosyltransferase | Transport_container |
| [O] | COG4133 | ABC-type transport system involved in cytochrome c biogenesis, ATPase component | Transport_container |
| [E] | COG4177 | ABC-type branched-chain amino acid transport system, permease component | Transport_container |
| [Q] | COG4181 | Predicted ABC-type transport system involved in lysophospholipase L1 biosynthesis, ATPase component | Transport_container |
| [S] | COG4260 | Putative virion core protein (lumpy skin disease virus) | Transport_container |
| [U] | COG4473 | Predicted ABC-type exoprotein transport system, permease component | Transport_container |
| [M] | COG4591 | ABC-type transport system, involved in lipoprotein release, permease component | Transport_container |
| [Q] | COG4664 | TRAP-type mannitol/chloroaromatic compound transport system, large permease component | Transport_container |
| [Q] | COG4665 | TRAP-type mannitol/chloroaromatic compound transport system, small permease component | Transport_container |
| [TK] | COG4725 | Transcriptional activator, adenine-specific DNA methyltransferase | Transport_container |
| [M] | COG4750 | CTP:phosphocholine cytidylyltransferase involved in choline phosphorylation for cell surface LPS epitopes | Transport_container |
| [I] | COG4799 | Acetyl-CoA carboxylase, carboxyltransferase component (subunits alpha and beta) | Transport_container |
| [CO] | COG4987 | ABC-type transport system involved in cytochrome bd biosynthesis, fused ATPase and permease components | Transport_container |
| [P] | COG5036 | SPX domain-containing protein involved in vacuolar polyphosphate accumulation | Transport_container |
| [O] | COG5044 | RAB proteins geranylgeranyltransferase component A (RAB escort protein) | Transport_container |
| [U] | COG5058 | Protein transporter of the TRAM (translocating chain-associating membrane) superfamily, longevity assurance factor | Transport_container |
| [U] | COG5102 | Membrane protein involved in ER to Golgi transport | Transport_container |
| [JU] | COG5117 | Protein involved in the nuclear export of pre-ribosomes | Transport_container |
| [O] | COG5265 | ABC-type transport system involved in Fe-S cluster assembly, permease and ATPase components | Transport_container |
| [U] | COG5314 | Conjugal transfer/entry exclusion protein | Transport_container |
| [O] | COG5596 | Mitochondrial import inner membrane translocase, subunit TIM22 | Transport_container |
| [K] | COG5624 | Transcription initiation factor TFIID, subunit TAF12 (also component of histone acetyltransferase SAGA) | Transport_container |
| [P] | COG0053 | Predicted Co/Zn/Cd cation transporters | Transport_container |
| [R] | COG0110 | Acetyltransferase (isoleucine patch superfamily) | Transport_container |
| [EJ] | COG0252 | L-asparaginase/archaeal Glu-tRNAGln amidotransferase subunit D | Transport_container |
| [P] | COG0659 | Sulfate permease and related transporters (MFS superfamily) | Transport_container |
| [P] | COG1055 | Na+/H+ antiporter NhaD and related arsenite permeases | Transport_container |
| [R] | COG1123 | ATPase components of various ABC-type transport systems, contain duplicated ATPase | Transport_container |
| [R] | COG1444 | Predicted P-loop ATPase fused to an acetyltransferase | Transport_container |
| [GM] | COG1682 | ABC-type polysaccharide/polyol phosphate export systems, permease component | Transport_container |
| [GT] | COG1762 | Phosphotransferase system mannitol/fructose-specific IIA domain (Ntr-type) | Transport_container |
| [P] | COG2076 | Membrane transporters of cations and cationic drugs | Transport_container |
| [P] | COG2216 | High-affinity K+ transport system, ATPase chain B | Transport_container |
| [M] | COG2230 | Cyclopropane fatty acid synthase and related methyltransferases | Transport_container |
| [P] | COG2895 | GTPases - Sulfate adenylate transferase subunit 1 | Transport_container |
| [R] | COG2984 | ABC-type uncharacterized transport system, periplasmic component | Transport_container |
| [H] | COG2998 | ABC-type tungstate transport system, permease component | Transport_container |
| [G] | COG3090 | TRAP-type C4-dicarboxylate transport system, small permease component | Transport_container |
| [R] | COG3218 | ABC-type uncharacterized transport system, auxiliary component | Transport_container |
| [P] | COG3221 | ABC-type phosphate/phosphonate transport system, periplasmic component | Transport_container |
| [G] | COG3444 | Phosphotransferase system, mannose/fructose/N-acetylgalactosamine-specific component IIB | Transport_container |
| [P] | COG3638 | ABC-type phosphate/phosphonate transport system, ATPase component | Transport_container |
| [P] | COG3639 | ABC-type phosphate/phosphonate transport system, permease component | Transport_container |
| [G] | COG3730 | Phosphotransferase system sorbitol-specific component IIC | Transport_container |
| [G] | COG3731 | Phosphotransferase system sorbitol-specific component IIA | Transport_container |
| [G] | COG3732 | Phosphotransferase system sorbitol-specific component IIBC | Transport_container |
| [G] | COG3839 | ABC-type sugar transport systems, ATPase components | Transport_container |
| [H] | COG3840 | ABC-type thiamine transport system, ATPase component | Transport_container |
| [E] | COG3842 | ABC-type spermidine/putrescine transport systems, ATPase components | Transport_container |
| [R] | COG3845 | ABC-type uncharacterized transport systems, ATPase components | Transport_container |
| [M] | COG3966 | Protein involved in D-alanine esterification of lipoteichoic acid and wall teichoic acid (D-alanine transfer protein) | Transport_container |
| [R] | COG4158 | Predicted ABC-type sugar transport system, permease component | Transport_container |
| [V] | COG4167 | ABC-type antimicrobial peptide transport system, ATPase component | Transport_container |
| [V] | COG4168 | ABC-type antimicrobial peptide transport system, permease component | Transport_container |
| [V] | COG4170 | ABC-type antimicrobial peptide transport system, ATPase component | Transport_container |
| [V] | COG4171 | ABC-type antimicrobial peptide transport system, permease component | Transport_container |
| [R] | COG4172 | ABC-type uncharacterized transport system, duplicated ATPase component | Transport_container |
| [E] | COG4175 | ABC-type proline/glycine betaine transport system, ATPase component | Transport_container |
| [E] | COG4176 | ABC-type proline/glycine betaine transport system, permease component | Transport_container |
| [P] | COG4651 | Kef-type K+ transport system, predicted NAD-binding component | Transport_container |
| [Q] | COG4663 | TRAP-type mannitol/chloroaromatic compound transport system, periplasmic component | Transport_container |
| [R] | COG4666 | TRAP-type uncharacterized transport system, fused permease components | Transport_container |
| [G] | COG4668 | Mannitol/fructose-specific phosphotransferase system, IIA domain | Transport_container |
| [CO] | COG4988 | ABC-type transport system involved in cytochrome bd biosynthesis, ATPase and permease components | Transport_container |
| [TA] | COG5238 | Ran GTPase-activating protein (RanGAP) involved in mRNA processing and transport | Transport_container |
| [U] | COG5249 | Golgi protein involved in Golgi-to-ER retrieval | Transport_container |
| [E] | COG0075 | Serine-pyruvate aminotransferase/archaeal aspartate aminotransferase | Transport_container |
| [HJ] | COG0189 | Glutathione synthase/Ribosomal protein S6 modification enzyme (glutaminyl transferase) | Transport_container |
| [V] | COG0286 | Type I restriction-modification system methyltransferase subunit | Transport_container |
| [F] | COG0299 | Folate-dependent phosphoribosylglycinamide formyltransferase PurN | Transport_container |
| [G] | COG0483 | Archaeal fructose-1,6-bisphosphatase and related enzymes of inositol monophosphatase family | Transport_container |
| [R] | COG0488 | ATPase components of ABC transporters with duplicated ATPase domains | Transport_container |
| [O] | COG0555 | ABC-type sulfate transport system, permease component | Transport_container |
| [P] | COG0581 | ABC-type phosphate transport system, permease component | Transport_container |
| [R] | COG1075 | Predicted acetyltransferases and hydrolases with the alpha/beta hydrolase fold | Transport_container |
| [G] | COG1299 | Phosphotransferase system, fructose-specific IIC component | Transport_container |
| [J] | COG1670 | Acetyltransferases, including N-acetylases of ribosomal proteins | Transport_container |
| [G] | COG1869 | ABC-type ribose transport system, auxiliary component | Transport_container |
| [P] | COG1930 | ABC-type cobalt transport system, periplasmic component | Transport_container |
| [C] | COG2025 | Electron transfer flavoprotein, alpha subunit | Transport_container |
| [I] | COG2031 | Short chain fatty acids transporter | Transport_container |
| [I] | COG2057 | Acyl CoA:acetate/3-ketoacid CoA transferase, beta subunit | Transport_container |
| [P] | COG2072 | Predicted flavoprotein involved in K+ transport | Transport_container |
| [V] | COG2274 | ABC-type bacteriocin/lantibiotic exporters, contain an N-terminal double-glycine peptidase domain | Transport_container |
| [R] | COG2401 | ABC-type ATPase fused to a predicted acetyltransferase domain | Transport_container |
| [L] | COG2946 | Putative phage replication protein RstA | Transport_container |
| [I] | COG3255 | Putative sterol carrier protein | Transport_container |
| [R] | COG3393 | Predicted acetyltransferase | Transport_container |
| [G] | COG3414 | Phosphotransferase system, galactitol-specific IIB component | Transport_container |
| [M] | COG3559 | Putative exporter of polyketide antibiotics | Transport_container |
| [C] | COG3630 | Na+-transporting methylmalonyl-CoA/oxaloacetate decarboxylase, gamma subunit | Transport_container |
| [G] | COG3715 | Phosphotransferase system, mannose/fructose/N-acetylgalactosamine-specific component IIC | Transport_container |
| [G] | COG3716 | Phosphotransferase system, mannose/fructose/N-acetylgalactosamine-specific component IID | Transport_container |
| [M] | COG3774 | Mannosyltransferase OCH1 and related enzymes | Transport_container |
| [G] | COG3775 | Phosphotransferase system, galactitol-specific IIC component | Transport_container |
| [R] | COG3818 | Predicted acetyltransferase, GNAT superfamily | Transport_container |
| [R] | COG4120 | ABC-type uncharacterized transport system, permease component | Transport_container |
| [P] | COG4150 | ABC-type sulfate transport system, periplasmic component | Transport_container |
| [R] | COG4152 | ABC-type uncharacterized transport system, ATPase component | Transport_container |
| [E] | COG4160 | ABC-type arginine/histidine transport system, permease component | Transport_container |
| [E] | COG4161 | ABC-type arginine transport system, ATPase component | Transport_container |
| [E] | COG4166 | ABC-type oligopeptide transport system, periplasmic component | Transport_container |
| [R] | COG4174 | ABC-type uncharacterized transport system, permease component | Transport_container |
| [F] | COG4360 | ATP adenylyltransferase (5',5'''-P-1,P-4-tetraphosphate phosphorylase II) | Transport_container |
| [E] | COG4597 | ABC-type amino acid transport system, permease component | Transport_container |
| [QP] | COG4615 | ABC-type siderophore export system, fused ATPase and permease components | Transport_container |
| [H] | COG4662 | ABC-type tungstate transport system, periplasmic component | Transport_container |
| [I] | COG4670 | Acyl CoA:acetate/3-ketoacid CoA transferase | Transport_container |
| [R] | COG4797 | Predicted regulatory domain of a methyltransferase | Transport_container |
| [R] | COG5038 | Ca2+-dependent lipid-binding protein, contains C2 domain | Transport_container |
| [B] | COG5114 | Histone acetyltransferase complex SAGA/ADA, subunit ADA2 | Transport_container |
| [U] | COG5120 | Membrane protein involved in Golgi transport | Transport_container |
| [U] | COG5122 | Transport protein particle (TRAPP) complex subunit | Transport_container |
| [C] | COG5127 | Vacuolar H+-ATPase V1 sector, subunit C | Transport_container |
| [U] | COG5128 | Transport protein particle (TRAPP) complex subunit | Transport_container |
| [O] | COG5536 | Protein prenyltransferase, alpha subunit | Transport_container |
| [M] | COG5581 | Predicted glycosyltransferase | Transport_container |
| [T] | COG5585 | NAD+asparagine ADP-ribosyltransferase | Transport_container |
| [H] | COG0001 | Glutamate-1-semialdehyde aminotransferase | Transport_container |
| [U] | COG0201 | Preprotein translocase subunit SecY | Transport_container |
| [R] | COG0220 | Predicted S-adenosylmethionine-dependent methyltransferase | Transport_container |
| [P] | COG0226 | ABC-type phosphate transport system, periplasmic component | Transport_container |
| [P] | COG0428 | Predicted divalent heavy-metal cations transporter | Transport_container |
| [KR] | COG0454 | Histone acetyltransferase HPA2 and related acetyltransferases | Transport_container |
| [J] | COG0482 | Predicted tRNA(5-methylaminomethyl-2-thiouridylate) methyltransferase, contains the PP-loop ATPase domain | Transport_container |
| [E] | COG0531 | Amino acid transporters | Transport_container |
| [V] | COG0577 | ABC-type antimicrobial peptide transport system, permease component | Transport_container |
| [U] | COG0706 | Preprotein translocase subunit YidC | Transport_container |
| [O] | COG0755 | ABC-type transport system involved in cytochrome c biogenesis, permease component | Transport_container |
| [V] | COG0842 | ABC-type multidrug transport system, permease component | Transport_container |
| [R] | COG1033 | Predicted exporters of the RND superfamily | Transport_container |
| [P] | COG1119 | ABC-type molybdenum transport system, ATPase component/photorepair protein PhrA | Transport_container |
| [E] | COG1126 | ABC-type polar amino acid transport system, ATPase component | Transport_container |
| [V] | COG1132 | ABC-type multidrug transport system, ATPase and permease components | Transport_container |
| [C] | COG1347 | Na+-transporting NADH:ubiquinone oxidoreductase, subunit NqrD | Transport_container |
| [M] | COG1368 | Phosphoglycerol transferase and related proteins, alkaline phosphatase superfamily | Transport_container |
| [G] | COG1445 | Phosphotransferase system fructose-specific component IIB | Transport_container |
| [G] | COG1447 | Phosphotransferase system cellobiose-specific component IIA | Transport_container |
| [G] | COG1455 | Phosphotransferase system cellobiose-specific component IIC | Transport_container |
| [P] | COG1464 | ABC-type metal ion transport system, periplasmic component/surface antigen | Transport_container |
| [P] | COG1563 | Predicted subunit of the Multisubunit Na+/H+ antiporter | Transport_container |
| [G] | COG1653 | ABC-type sugar transport system, periplasmic component | Transport_container |
| [C] | COG1805 | Na+-transporting NADH:ubiquinone oxidoreductase, subunit NqrB | Transport_container |
| [GC] | COG1819 | Glycosyl transferases, related to UDP-glucuronosyltransferase | Transport_container |
| [P] | COG1824 | Permease, similar to cation transporters | Transport_container |
| [P] | COG1840 | ABC-type Fe3+ transport system, periplasmic component | Transport_container |
| [P] | COG1863 | Multisubunit Na+/H+ antiporter, MnhE subunit | Transport_container |
| [G] | COG1879 | ABC-type sugar transport system, periplasmic component | Transport_container |
| [P] | COG1918 | Fe2+ transport system protein A | Transport_container |
| [H] | COG1962 | Tetrahydromethanopterin S-methyltransferase, subunit H | Transport_container |
| [F] | COG2065 | Pyrimidine operon attenuation protein/uracil phosphoribosyltransferase | Transport_container |
| [I] | COG2067 | Long-chain fatty acid transport protein | Transport_container |
| [C] | COG2086 | Electron transfer flavoprotein, beta subunit | Transport_container |
| [H] | COG2087 | Adenosyl cobinamide kinase/adenosyl cobinamide phosphate guanylyltransferase | Transport_container |
| [E] | COG2113 | ABC-type proline/glycine betaine transport systems, periplasmic components | Transport_container |
| [P] | COG2156 | K+-transporting ATPase, c chain | Transport_container |
| [J] | COG2519 | tRNA(1-methyladenosine) methyltransferase and related methyltransferases | Transport_container |
| [GE] | COG2610 | H+/gluconate symporter and related permeases | Transport_container |
| [C] | COG2811 | Archaeal/vacuolar-type H+-ATPase subunit H | Transport_container |
| [G] | COG2893 | Phosphotransferase system, mannose/fructose-specific component IIA | Transport_container |
| [R] | COG2933 | Predicted SAM-dependent methyltransferase | Transport_container |
| [O] | COG2935 | Putative arginyl-tRNA:protein arginylyltransferase | Transport_container |
| [Q] | COG2977 | Phosphopantetheinyl transferase component of siderophore synthetase | Transport_container |
| [O] | COG2994 | ACP:hemolysin acyltransferase (hemolysin-activating protein) | Transport_container |
| [M] | COG3061 | Cell envelope opacity-associated protein A | Transport_container |
| [E] | COG3138 | Arginine/ornithine N-succinyltransferase beta subunit | Transport_container |
| [R] | COG3173 | Predicted aminoglycoside phosphotransferase | Transport_container |
| [R] | COG3442 | Predicted glutamine amidotransferase | Transport_container |
| [M] | COG3524 | Capsule polysaccharide export protein | Transport_container |
| [M] | COG3562 | Capsule polysaccharide export protein | Transport_container |
| [M] | COG3563 | Capsule polysaccharide export protein | Transport_container |
| [R] | COG3683 | ABC-type uncharacterized transport system, periplasmic component | Transport_container |
| [R] | COG3822 | ABC-type sugar transport system, auxiliary component | Transport_container |
| [G] | COG3833 | ABC-type maltose transport systems, permease component | Transport_container |
| [V] | COG3896 | Chloramphenicol 3-O-phosphotransferase | Transport_container |
| [R] | COG3897 | Predicted methyltransferase | Transport_container |
| [R] | COG4178 | ABC-type uncharacterized transport system, permease and ATPase components | Transport_container |
| [T] | COG4191 | Signal transduction histidine kinase regulating C4-dicarboxylate transport system | Transport_container |
| [T] | COG4192 | Signal transduction histidine kinase regulating phosphoglycerate transport system | Transport_container |
| [R] | COG4586 | ABC-type uncharacterized transport system, ATPase component | Transport_container |
| [R] | COG4587 | ABC-type uncharacterized transport system, permease component | Transport_container |
| [R] | COG4590 | ABC-type uncharacterized transport system, permease component | Transport_container |
| [P] | COG4592 | ABC-type Fe2+-enterobactin transport system, periplasmic component | Transport_container |
| [P] | COG4594 | ABC-type Fe3+-citrate transport system, periplasmic component | Transport_container |
| [E] | COG4598 | ABC-type histidine transport system, ATPase component | Transport_container |
| [R] | COG4603 | ABC-type uncharacterized transport system, permease component | Transport_container |
| [P] | COG4604 | ABC-type enterochelin transport system, ATPase component | Transport_container |
| [P] | COG4605 | ABC-type enterochelin transport system, permease component | Transport_container |
| [P] | COG4606 | ABC-type enterochelin transport system, permease component | Transport_container |
| [R] | COG4618 | ABC-type protease/lipase transport system, ATPase and permease components | Transport_container |
| [R] | COG4671 | Predicted glycosyl transferase | Transport_container |
| [P] | COG4778 | ABC-type phosphonate transport system, ATPase component | Transport_container |
| [P] | COG4779 | ABC-type enterobactin transport system, permease component | Transport_container |
| [F] | COG0047 | Phosphoribosylformylglycinamidine (FGAM) synthase, glutamine amidotransferase domain | Transport_container |
| [E] | COG0078 | Ornithine carbamoyltransferase | Transport_container |
| [E] | COG0160 | 4-aminobutyrate aminotransferase and related aminotransferases | Transport_container |
| [J] | COG0223 | Methionyl-tRNA formyltransferase | Transport_container |
| [P] | COG0475 | Kef-type K+ transport systems, membrane components | Transport_container |
| [P] | COG0573 | ABC-type phosphate transport system, permease component | Transport_container |
| [P] | COG0600 | ABC-type nitrate/sulfonate/bicarbonate transport system, permease component | Transport_container |
| [EP] | COG0601 | ABC-type dipeptide/oligopeptide/nickel transport systems, permease components | Transport_container |
| [P] | COG0609 | ABC-type Fe3+-siderophore transport system, permease component | Transport_container |
| [P] | COG0614 | ABC-type Fe3+-hydroxamate transport system, periplasmic component | Transport_container |
| [M] | COG0707 | UDP-N-acetylglucosamine:LPS N-acetylglucosamine transferase | Transport_container |
| [P] | COG0715 | ABC-type nitrate/sulfonate/bicarbonate transport systems, periplasmic components | Transport_container |
| [P] | COG0725 | ABC-type molybdate transport system, periplasmic component | Transport_container |
| [E] | COG0765 | ABC-type amino acid transport system, permease component | Transport_container |
| [E] | COG0786 | Na+/glutamate symporter | Transport_container |
| [H] | COG1057 | Nicotinic acid mononucleotide adenylyltransferase | Transport_container |
| [R] | COG1100 | GTPase SAR1 and related small G proteins | Transport_container |
| [E] | COG1125 | ABC-type proline/glycine betaine transport systems, ATPase components | Transport_container |
| [GM] | COG1134 | ABC-type polysaccharide/polyol phosphate transport system, ATPase component | Transport_container |
| [P] | COG1135 | ABC-type metal ion transport system, ATPase component | Transport_container |
| [V] | COG1136 | ABC-type antimicrobial peptide transport system, ATPase component | Transport_container |
| [M] | COG1207 | N-acetylglucosamine-1-phosphate uridyltransferase (contains nucleotidyltransferase and I-patch acetyltransferase domains) | Transport_container |
| [M] | COG1215 | Glycosyltransferases, probably involved in cell wall biogenesis | Transport_container |
| [P] | COG1226 | Kef-type K+ transport systems, predicted NAD-binding component | Transport_container |
| [E] | COG1246 | N-acetylglutamate synthase and related acetyltransferases | Transport_container |
| [M] | COG1442 | Lipopolysaccharide biosynthesis proteins, LPS:glycosyltransferases | Transport_container |
| [H] | COG1488 | Nicotinic acid phosphoribosyltransferase | Transport_container |
| [P] | COG1613 | ABC-type sulfate transport system, periplasmic component | Transport_container |
| [C] | COG1804 | Predicted acyl-CoA transferases/carnitine dehydratase | Transport_container |
| [G] | COG1925 | Phosphotransferase system, HPr-related proteins | Transport_container |
| [O] | COG2020 | Putative protein-S-isoprenylcysteine methyltransferase | Transport_container |
| [P] | COG2046 | ATP sulfurylase (sulfate adenylyltransferase) | Transport_container |
| [P] | COG2059 | Chromate transport protein ChrA | Transport_container |
| [P] | COG2060 | K+-transporting ATPase, A chain | Transport_container |
| [O] | COG2360 | Leu/Phe-tRNA-protein transferase | Transport_container |
| [R] | COG2409 | Predicted drug exporters of the RND superfamily | Transport_container |
| [I] | COG2937 | Glycerol-3-phosphate O-acyltransferase | Transport_container |
| [G] | COG2956 | Predicted N-acetylglucosaminyl transferase | Transport_container |
| [P] | COG3067 | Na+/H+ antiporter | Transport_container |
| [C] | COG3069 | C4-dicarboxylate transporter | Transport_container |
| [R] | COG3129 | Predicted SAM-dependent methyltransferase | Transport_container |
| [C] | COG3493 | Na+/citrate symporter | Transport_container |
| [P] | COG4107 | ABC-type phosphonate transport system, ATPase component | Transport_container |
| [R] | COG4132 | ABC-type uncharacterized transport system, permease component | Transport_container |
| [R] | COG4134 | ABC-type uncharacterized transport system, periplasmic component | Transport_container |
| [R] | COG4135 | ABC-type uncharacterized transport system, permease component | Transport_container |
| [R] | COG4136 | ABC-type uncharacterized transport system, ATPase component | Transport_container |
| [R] | COG4137 | ABC-type uncharacterized transport system, permease component | Transport_container |
| [H] | COG4138 | ABC-type cobalamin transport system, ATPase component | Transport_container |
| [H] | COG4139 | ABC-type cobalamin transport system, permease component | Transport_container |
| [H] | COG4143 | ABC-type thiamine transport system, periplasmic component | Transport_container |
| [P] | COG4148 | ABC-type molybdate transport system, ATPase component | Transport_container |
| [P] | COG4149 | ABC-type molybdate transport system, permease component | Transport_container |
| [P] | COG4208 | ABC-type sulfate transport system, permease component | Transport_container |
| [G] | COG4209 | ABC-type polysaccharide transport system, permease component | Transport_container |
| [G] | COG4211 | ABC-type glucose/galactose transport system, permease component | Transport_container |
| [G] | COG4213 | ABC-type xylose transport system, periplasmic component | Transport_container |
| [G] | COG4214 | ABC-type xylose transport system, permease component | Transport_container |
| [E] | COG4215 | ABC-type arginine transport system, permease component | Transport_container |
| [E] | COG4413 | Urea transporter | Transport_container |
| [R] | COG4552 | Predicted acetyltransferase involved in intracellular survival and related acetyltransferases | Transport_container |
| [U] | COG5043 | Vacuolar protein sorting-associated protein | Transport_container |
| [O] | COG5264 | Vacuolar transporter chaperone | Transport_container |
| [I] | COG0204 | 1-acyl-sn-glycerol-3-phosphate acyltransferase | Transport_container |
| [R] | COG0313 | Predicted methyltransferases | Transport_container |
| [V] | COG0534 | Na+-driven multidrug efflux pump | Transport_container |
| [P] | COG0569 | K+ transport systems, NAD-binding component | Transport_container |
| [U] | COG0823 | Periplasmic component of the Tol biopolymer transport system | Transport_container |
| [E] | COG0833 | Amino acid transporters | Transport_container |
| [H] | COG1056 | Nicotinamide mononucleotide adenylyltransferase | Transport_container |
| [R] | COG1101 | ABC-type uncharacterized transport system, ATPase component | Transport_container |
| [P] | COG1116 | ABC-type nitrate/sulfonate/bicarbonate transport system, ATPase component | Transport_container |
| [P] | COG1117 | ABC-type phosphate transport system, ATPase component | Transport_container |
| [P] | COG1118 | ABC-type sulfate/molybdate transport systems, ATPase component | Transport_container |
| [PH] | COG1120 | ABC-type cobalamin/Fe3+-siderophores transport systems, ATPase components | Transport_container |
| [P] | COG1121 | ABC-type Mn/Zn transport systems, ATPase component | Transport_container |
| [P] | COG1122 | ABC-type cobalt transport system, ATPase component | Transport_container |
| [EP] | COG1124 | ABC-type dipeptide/oligopeptide/nickel transport system, ATPase component | Transport_container |
| [G] | COG1129 | ABC-type sugar transport system, ATPase component | Transport_container |
| [V] | COG1131 | ABC-type multidrug transport system, ATPase component | Transport_container |
| [R] | COG1137 | ABC-type (unclassified) transport system, ATPase component | Transport_container |
| [M] | COG1247 | Sortase and related acyltransferases | Transport_container |
| [G] | COG1263 | Phosphotransferase system IIC components, glucose/maltose/N-acetylglucosamine-specific | Transport_container |
| [C] | COG1301 | Na+/H+-dicarboxylate symporters | Transport_container |
| [C] | COG1390 | Archaeal/vacuolar-type H+-ATPase subunit E | Transport_container |
| [C] | COG1394 | Archaeal/vacuolar-type H+-ATPase subunit D | Transport_container |
| [C] | COG1527 | Archaeal/vacuolar-type H+-ATPase subunit C | Transport_container |
| [J] | COG1549 | Queuine tRNA-ribosyltransferases, contain PUA domain | Transport_container |
| [G] | COG1640 | 4-alpha-glucanotransferase | Transport_container |
| [C] | COG1757 | Na+/H+ antiporter | Transport_container |
| [J] | COG1798 | Diphthamide biosynthesis methyltransferase | Transport_container |
| [R] | COG1823 | Predicted Na+/dicarboxylate symporter | Transport_container |
| [J] | COG1867 | N2,N2-dimethylguanosine tRNA methyltransferase | Transport_container |
| [O] | COG1928 | Dolichyl-phosphate-mannoseprotein O-mannosyl transferase | Transport_container |
| [E] | COG2021 | Homoserine acetyltransferase | Transport_container |
| [C] | COG2037 | Formylmethanofuran:tetrahydromethanopterin formyltransferase | Transport_container |
| [H] | COG2038 | NaMN:DMB phosphoribosyltransferase | Transport_container |
| [R] | COG2071 | Predicted glutamine amidotransferases | Transport_container |
| [P] | COG2111 | Multisubunit Na+/H+ antiporter, MnhB subunit | Transport_container |
| [M] | COG2148 | Sugar transferases involved in lipopolysaccharide synthesis | Transport_container |
| [R] | COG2153 | Predicted acyltransferase | Transport_container |
| [Q] | COG2162 | Arylamine N-acetyltransferase | Transport_container |
| [P] | COG2217 | Cation transport ATPase | Transport_container |
| [P] | COG2239 | Mg/Co/Ni transporter MgtE (contains CBS domain) | Transport_container |
| [J] | COG2265 | SAM-dependent methyltransferases related to tRNA (uracil-5-)-methyltransferase | Transport_container |
| [R] | COG2521 | Predicted archaeal methyltransferase | Transport_container |
| [R] | COG2704 | Anaerobic C4-dicarboxylate transporter | Transport_container |
| [M] | COG2943 | Membrane glycosyltransferase | Transport_container |
| [P] | COG3004 | Na+/H+ antiporter | Transport_container |
| [M] | COG3306 | Glycosyltransferase involved in LPS biosynthesis | Transport_container |
| [G] | COG3594 | Fucose 4-O-acetylase and related acetyltransferases | Transport_container |
| [E] | COG3633 | Na+/serine symporter | Transport_container |
| [R] | COG3694 | ABC-type uncharacterized transport system, permease component | Transport_container |
| [E] | COG3705 | ATP phosphoribosyltransferase involved in histidine biosynthesis | Transport_container |
| [R] | COG3981 | Predicted acetyltransferase | Transport_container |
| [H] | COG4059 | Tetrahydromethanopterin S-methyltransferase, subunit E | Transport_container |
| [H] | COG4060 | Tetrahydromethanopterin S-methyltransferase, subunit D | Transport_container |
| [H] | COG4061 | Tetrahydromethanopterin S-methyltransferase, subunit C | Transport_container |
| [H] | COG4062 | Tetrahydromethanopterin S-methyltransferase, subunit B | Transport_container |
| [H] | COG4063 | Tetrahydromethanopterin S-methyltransferase, subunit A | Transport_container |
| [H] | COG4064 | Tetrahydromethanopterin S-methyltransferase, subunit G | Transport_container |
| [M] | COG4092 | Predicted glycosyltransferase involved in capsule biosynthesis | Transport_container |
| [G] | COG4468 | Galactose-1-phosphate uridyltransferase | Transport_container |
| [R] | COG4589 | Predicted CDP-diglyceride synthetase/phosphatidate cytidylyltransferase | Transport_container |
| [P] | COG4607 | ABC-type enterochelin transport system, periplasmic component | Transport_container |
| [E] | COG4608 | ABC-type oligopeptide transport system, ATPase component | Transport_container |
| [R] | COG4619 | ABC-type uncharacterized transport system, ATPase component | Transport_container |
| [E] | COG4812 | Ethanolamine utilization cobalamin adenosyltransferase | Transport_container |
| [B] | COG5027 | Histone acetyltransferase (MYST family) | Transport_container |
| [TG] | COG5037 | Gluconate transport-inducing protein | Transport_container |
| [U] | COG5073 | Vacuolar import and degradation protein | Transport_container |
| [M] | COG5597 | Alpha-N-acetylglucosamine transferase | Transport_container |
| [H] | COG5598 | Trimethylamine:corrinoid methyltransferase | Transport_container |
| [O] | COG5656 | Importin, protein involved in nuclear import | Transport_container |
| [F] | COG0027 | Formate-dependent phosphoribosylglycinamide formyltransferase (GAR transformylase) | Transport_container |
| [R] | COG0121 | Predicted glutamine amidotransferase | Transport_container |
| [IQ] | COG0236 | Acyl carrier protein | Transport_container |
| [R] | COG0390 | ABC-type uncharacterized transport system, permease component | Transport_container |
| [P] | COG0471 | Di- and tricarboxylate transporters | Transport_container |
| [M] | COG0472 | UDP-N-acetylmuramyl pentapeptide phosphotransferase/UDP-N-acetylglucosamine-1-phosphate transferase | Transport_container |
| [P] | COG0598 | Mg2+ and Co2+ transporters | Transport_container |
| [M] | COG0682 | Prolipoprotein diacylglyceryltransferase | Transport_container |
| [H] | COG0684 | Demethylmenaquinone methyltransferase | Transport_container |
| [J] | COG0721 | Asp-tRNAAsn/Glu-tRNAGln amidotransferase C subunit | Transport_container |
| [R] | COG0733 | Na+-dependent transporters of the SNF family | Transport_container |
| [C] | COG0822 | NifU homolog involved in Fe-S cluster formation | Transport_container |
| [M] | COG0836 | Mannose-1-phosphate guanylyltransferase | Transport_container |
| [V] | COG0841 | Cation/multidrug efflux pump | Transport_container |
| [U] | COG0848 | Biopolymer transport protein | Transport_container |
| [F] | COG0856 | Orotate phosphoribosyltransferase homologs | Transport_container |
| [R] | COG1019 | Predicted nucleotidyltransferase | Transport_container |
| [E] | COG1045 | Serine acetyltransferase | Transport_container |
| [R] | COG1054 | Predicted sulfurtransferase | Transport_container |
| [G] | COG1264 | Phosphotransferase system IIB components | Transport_container |
| [P] | COG1276 | Putative copper export protein | Transport_container |
| [OT] | COG1391 | Glutamine synthetase adenylyltransferase | Transport_container |
| [E] | COG1448 | Aspartate/tyrosine/aromatic aminotransferase | Transport_container |
| [M] | COG1519 | 3-deoxy-D-manno-octulosonic-acid transferase | Transport_container |
| [G] | COG1593 | TRAP-type C4-dicarboxylate transport system, large permease component | Transport_container |
| [R] | COG1669 | Predicted nucleotidyltransferases | Transport_container |
| [R] | COG1926 | Predicted phosphoribosyltransferases | Transport_container |
| [HE] | COG1932 | Phosphoserine aminotransferase | Transport_container |
| [P] | COG2116 | Formate/nitrite family of transporters | Transport_container |
| [E] | COG2171 | Tetrahydrodipicolinate N-succinyltransferase | Transport_container |
| [R] | COG2215 | ABC-type uncharacterized transport system, permease component | Transport_container |
| [P] | COG2223 | Nitrate/nitrite transporter | Transport_container |
| [O] | COG2518 | Protein-L-isoaspartate carboxylmethyltransferase | Transport_container |
| [R] | COG2520 | Predicted methyltransferase | Transport_container |
| [P] | COG2897 | Rhodanese-related sulfurtransferase | Transport_container |
| [H] | COG2978 | Putative p-aminobenzoyl-glutamate transporter | Transport_container |
| [I] | COG3154 | Putative lipid carrier protein | Transport_container |
| [H] | COG3201 | Nicotinamide mononucleotide transporter | Transport_container |
| [F] | COG3613 | Nucleoside 2-deoxyribosyltransferase | Transport_container |
| [E] | COG3643 | Glutamate formiminotransferase | Transport_container |
| [L] | COG3695 | Predicted methylated DNA-protein cysteine methyltransferase | Transport_container |
| [R] | COG4122 | Predicted O-methyltransferase | Transport_container |
| [R] | COG4123 | Predicted O-methyltransferase | Transport_container |
| [H] | COG4218 | Tetrahydromethanopterin S-methyltransferase, subunit F | Transport_container |
| [R] | COG4261 | Predicted acyltransferase | Transport_container |
| [R] | COG4798 | Predicted methyltransferase | Transport_container |
| [R] | COG4801 | Predicted acyltransferase | Transport_container |
| [V] | COG4845 | Chloramphenicol O-acetyltransferase | Transport_container |
| [R] | COG4914 | Predicted nucleotidyltransferase | Transport_container |
| [P] | COG4986 | ABC-type anion transport system, duplicated permease component | Transport_container |
| [O] | COG5029 | Prenyltransferase, beta subunit | Transport_container |
| [P] | COG5065 | Protein involved in inorganic phosphate transport | Transport_container |
| [P] | COG5266 | ABC-type Co2+ transport system, periplasmic component | Transport_container |
| [I] | COG5379 | S-adenosylmethionine:diacylglycerol 3-amino-3-carboxypropyl transferase | Transport_container |
| [R] | COG5628 | Predicted acetyltransferase | Transport_container |
| [J] | COG0030 | Dimethyladenosine transferase (rRNA methylation) | Transport_container |
| [F] | COG0034 | Glutamine phosphoribosylpyrophosphate amidotransferase | Transport_container |
| [E] | COG0112 | Glycine/serine hydroxymethyltransferase | Transport_container |
| [E] | COG0118 | Glutamine amidotransferase | Transport_container |
| [H] | COG0161 | Adenosylmethionine-8-amino-7-oxononanoate aminotransferase | Transport_container |
| [L] | COG0178 | Excinuclease ATPase subunit | Transport_container |
| [C] | COG0280 | Phosphotransacetylase | Transport_container |
| [F] | COG0461 | Orotate phosphoribosyltransferase | Transport_container |
| [P] | COG0474 | Cation transport ATPase | Transport_container |
| [I] | COG0511 | Biotin carboxyl carrier protein | Transport_container |
| [I] | COG0764 | 3-hydroxymyristoyl/3-hydroxydecanoyl-(acyl carrier protein) dehydratases | Transport_container |
| [R] | COG0857 | BioD-like N-terminal domain of phosphotransacetylase | Transport_container |
| [P] | COG1006 | Multisubunit Na+/H+ antiporter, MnhC subunit | Transport_container |
| [R] | COG1040 | Predicted amidophosphoribosyltransferases | Transport_container |
| [R] | COG1092 | Predicted SAM-dependent methyltransferases | Transport_container |
| [KB] | COG1243 | Histone acetyltransferase | Transport_container |
| [P] | COG1320 | Multisubunit Na+/H+ antiporter, MnhG subunit | Transport_container |
| [M] | COG1596 | Periplasmic protein involved in polysaccharide export | Transport_container |
| [I] | COG1788 | Acyl CoA:acetate/3-ketoacid CoA transferase, alpha subunit | Transport_container |
| [I] | COG1835 | Predicted acyltransferases | Transport_container |
| [J] | COG1859 | RNA:NAD 2'-phosphotransferase | Transport_container |
| [O] | COG2192 | Predicted carbamoyl transferase, NodU family | Transport_container |
| [C] | COG2209 | Na+-transporting NADH:ubiquinone oxidoreductase, subunit NqrE | Transport_container |
| [G] | COG2211 | Na+/melibiose symporter and related transporters | Transport_container |
| [P] | COG2212 | Multisubunit Na+/H+ antiporter, MnhF subunit | Transport_container |
| [G] | COG2213 | Phosphotransferase system, mannitol-specific IIBC component | Transport_container |
| [R] | COG2384 | Predicted SAM-dependent methyltransferase | Transport_container |
| [R] | COG2391 | Predicted transporter component | Transport_container |
| [O] | COG2844 | UTP:GlnB (protein PII) uridylyltransferase | Transport_container |
| [U] | COG3114 | Heme exporter protein D | Transport_container |
| [R] | COG3541 | Predicted nucleotidyltransferase | Transport_container |
| [P] | COG3696 | Putative silver efflux pump | Transport_container |
| [HI] | COG3697 | Phosphoribosyl-dephospho-CoA transferase (holo-ACP synthetase) | Transport_container |
| [O] | COG3823 | Glutamine cyclotransferase | Transport_container |
| [P] | COG4531 | ABC-type Zn2+ transport system, periplasmic component/surface adhesin | Transport_container |
| [CP] | COG4555 | ABC-type Na+ transport system, ATPase component | Transport_container |
| [P] | COG4558 | ABC-type hemin transport system, periplasmic component | Transport_container |
| [P] | COG4559 | ABC-type hemin transport system, ATPase component | Transport_container |
| [R] | COG4827 | Predicted transporter | Transport_container |
| [R] | COG4976 | Predicted methyltransferase (contains TPR repeat) | Transport_container |
| [P] | COG4985 | ABC-type phosphate transport system, auxiliary component | Transport_container |
| [U] | COG5215 | Karyopherin (importin) beta | Transport_container |
| [F] | COG0035 | Uracil phosphoribosyltransferase | Transport_container |
| [E] | COG0040 | ATP phosphoribosyltransferase | Transport_container |
| [I] | COG0183 | Acetyl-CoA acetyltransferase | Transport_container |
| [R] | COG0456 | Acetyltransferases | Transport_container |
| [R] | COG0663 | Carbonic anhydrases/acetyltransferases, isoleucine patch superfamily | Transport_container |
| [I] | COG0736 | Phosphopantetheinyl transferase (holo-ACP synthase) | Transport_container |
| [R] | COG1078 | HD superfamily phosphohydrolases | Transport_container |
| [C] | COG1085 | Galactose-1-phosphate uridylyltransferase | Transport_container |
| [M] | COG1213 | Predicted sugar nucleotidyltransferases | Transport_container |
| [G] | COG1440 | Phosphotransferase system cellobiose-specific component IIB | Transport_container |
| [M] | COG1560 | Lauroyl/myristoyl acyltransferase | Transport_container |
| [R] | COG1568 | Predicted methyltransferases | Transport_container |
| [H] | COG1575 | 1,4-dihydroxy-2-naphthoate octaprenyltransferase | Transport_container |
| [R] | COG1708 | Predicted nucleotidyltransferases | Transport_container |
| [J] | COG1746 | tRNA nucleotidyltransferase (CCA-adding enzyme) | Transport_container |
| [H] | COG2109 | ATP:corrinoid adenosyltransferase | Transport_container |
| [G] | COG2190 | Phosphotransferase system IIA components | Transport_container |
| [R] | COG2388 | Predicted acetyltransferase | Transport_container |
| [R] | COG3153 | Predicted acetyltransferase | Transport_container |
| [P] | COG3158 | K+ transporter | Transport_container |
| [J] | COG3231 | Aminoglycoside phosphotransferase | Transport_container |
| [Q] | COG3433 | Aryl carrier domain | Transport_container |
| [R] | COG4106 | Trans-aconitate methyltransferase | Transport_container |
| [H] | COG4145 | Na+/panthothenate symporter | Transport_container |
| [R] | COG4146 | Predicted symporter | Transport_container |
| [R] | COG4147 | Predicted symporter | Transport_container |
| [R] | COG4239 | ABC-type uncharacterized transport system, permease component | Transport_container |
| [P] | COG4521 | ABC-type taurine transport system, periplasmic component | Transport_container |
| [P] | COG4525 | ABC-type taurine transport system, ATPase component | Transport_container |
| [R] | COG4533 | ABC-type uncharacterized transport system, periplasmic component | Transport_container |
| [P] | COG4535 | Putative Mg2+ and Co2+ transporter CorC | Transport_container |
| [P] | COG4536 | Putative Mg2+ and Co2+ transporter CorB | Transport_container |
| [O] | COG4745 | Predicted membrane-bound mannosyltransferase | Transport_container |
| [R] | COG4756 | Predicted cation transporter | Transport_container |
| [G] | COG5020 | Mannosyltransferase | Transport_container |
| [I] | COG5050 | sn-1,2-diacylglycerol ethanolamine- and cholinephosphotranferases | Transport_container |
| [GOU] | COG5070 | Nucleotide-sugar transporter | Transport_container |
| [OU] | COG5082 | Arginine methyltransferase-interacting protein, contains RING Zn-finger | Transport_container |
| [A] | COG5226 | mRNA capping enzyme, guanylyltransferase (alpha) subunit | Transport_container |
| [G] | COG0176 | Transaldolase | Transport_container |
| [R] | COG0385 | Predicted Na+-dependent transporter | Transport_container |
| [QR] | COG0500 | SAM-dependent methyltransferases | Transport_container |
| [L] | COG0675 | Transposase and inactivated derivatives | Transport_container |
| [E] | COG1115 | Na+/alanine symporter | Transport_container |
| [R] | COG1216 | Predicted glycosyltransferases | Transport_container |
| [C] | COG1726 | Na+-transporting NADH:ubiquinone oxidoreductase, subunit NqrA | Transport_container |
| [R] | COG2236 | Predicted phosphoribosyltransferases | Transport_container |
| [H] | COG2266 | GTP:adenosylcobinamide-phosphate guanylyltransferase | Transport_container |
| [R] | COG2413 | Predicted nucleotidyltransferase | Transport_container |
| [V] | COG2746 | Aminoglycoside N3'-acetyltransferase | Transport_container |
| [C] | COG2851 | H+/citrate symporter | Transport_container |
| [I] | COG5056 | Acyl-CoA cholesterol acyltransferase | Transport_container |
| [U] | COG0811 | Biopolymer transport proteins | Transport_container |
| [M] | COG0859 | ADP-heptose:LPS heptosyltransferase | Transport_container |
| [E] | COG1174 | ABC-type proline/glycine betaine transport systems, permease component | Transport_container |
| [E] | COG1176 | ABC-type spermidine/putrescine transport system, permease component I | Transport_container |
| [E] | COG1177 | ABC-type spermidine/putrescine transport system, permease component II | Transport_container |
| [I] | COG1182 | Acyl carrier protein phosphodiesterase | Transport_container |
| [J] | COG1185 | Polyribonucleotide nucleotidyltransferase (polynucleotide phosphorylase) | Transport_container |
| [R] | COG1323 | Predicted nucleotidyltransferase | Transport_container |
| [QP] | COG4242 | Cyanophycinase and related exopeptidases | Transport_container |
| [H] | COG0669 | Phosphopantetheine adenylyltransferase | Transport_container |
| [M] | COG0815 | Apolipoprotein N-acyltransferase | Transport_container |
| [G] | COG1172 | Ribose/xylose/arabinose/galactoside ABC-type transport systems, permease components | Transport_container |
| [EP] | COG1173 | ABC-type dipeptide/oligopeptide/nickel transport systems, permease components | Transport_container |
| [G] | COG1175 | ABC-type sugar transport systems, permease components | Transport_container |
| [P] | COG1178 | ABC-type Fe3+ transport system, permease component | Transport_container |
| [E] | COG4992 | Ornithine/acetylornithine aminotransferase | Transport_container |
| [F] | COG0634 | Hypoxanthine-guanine phosphoribosyltransferase | Transport_container |
| [C] | COG2869 | Na+-transporting NADH:ubiquinone oxidoreductase, subunit NqrC | Transport_container |
| [M] | COG2870 | ADP-heptose synthase, bifunctional sugar kinase/adenylyltransferase | Transport_container |
| [C] | COG2871 | Na+-transporting NADH:ubiquinone oxidoreductase, subunit NqrF | Transport_container |
| [P] | COG3965 | Predicted Co/Zn/Cd cation transporters | Transport_container |
| [R] | COG3969 | Predicted phosphoadenosine phosphosulfate sulfotransferase | Transport_container |
| [E] | COG3977 | Alanine-alpha-ketoisovalerate (or valine-pyruvate) aminotransferase | Transport_container |
| [C] | COG5016 | Pyruvate/oxaloacetate carboxyltransferase | Transport_container |
| [I] | COG5092 | N-myristoyl transferase | Transport_container |
| [UW] | COG5295 | Autotransporter adhesin | Transport_container |
| [I] | COG2867 | Oligoketide cyclase/lipid transport protein | Transport_container |
| [I] | COG3963 | Phospholipid N-methyltransferase | Transport_container |
| [R] | COG4258 | Predicted exporter | Transport_container |
| [M] | COG1043 | Acyl-[acyl carrier protein] UDP-N-acetylglucosamine O-acyltransferase " | Transport_container |
| [OJ] | COG5193 | La protein, small RNA-binding pol III transcript stabilizing protein and related La-motif-containing proteins involved in translation | Whole_complex |
| [R] | COG5553 | Predicted metal-dependent enzyme of the double-stranded beta helix superfamily | Whole_complex |
| [R] | COG5512 | Zn-ribbon-containing, possibly RNA-binding protein and truncated derivatives | Whole_complex |
| [C] | COG2609 | Pyruvate dehydrogenase complex, dehydrogenase (E1) component | Whole_complex |
| [S] | COG4886 | Leucine-rich repeat (LRR) protein | Whole_complex |
| [C] | COG3658 | Cytochrome b | Whole_complex |
| [V] | COG4268 | McrBC 5-methylcytosine restriction system component | Whole_complex |
| [C] | COG4314 | Predicted lipoprotein involved in nitrous oxide reduction | Whole_complex |
| [R] | COG4734 | Antirestriction protein | Whole_complex |
| [G] | COG5309 | Exo-beta-1,3-glucanase | Whole_complex |
| [R] | COG2373 | Large extracellular alpha-helical protein | Whole_complex |
| [C] | COG3245 | Cytochrome c5 | Whole_complex |
| [C] | COG3258 | Cytochrome c | Whole_complex |
| [C] | COG3783 | Soluble cytochrome b562 | Whole_complex |
| [C] | COG3909 | Cytochrome c556 | Whole_complex |
| [F] | COG4290 | Guanyl-specific ribonuclease Sa | Whole_complex |
| [D] | COG5072 | Serine/threonine kinase of the haspin family | Whole_complex |
| [N] | COG5555 | Cytolysin, a secreted calcineurin-like phosphatase | Whole_complex |
| [T] | COG5599 | Protein tyrosine phosphatase | Whole_complex |
| [L] | COG5600 | Transcription-associated recombination protein | Whole_complex |
| [S] | COG5608 | Conserved secreted protein | Whole_complex |
| [T] | COG5635 | Predicted NTPase (NACHT family) | Whole_complex |
| [Q] | COG2124 | Cytochrome P450 | Whole_complex |
| [R] | COG2374 | Predicted extracellular nuclease | Whole_complex |
| [R] | COG4469 | Competence protein | Whole_complex |
| [T] | COG5040 | 14.03.2003 family protein | Whole_complex |
| [B] | COG5262 | Histone H2A | Whole_complex |
| [T] | COG5408 | SPX domain-containing protein | Whole_complex |
| [D] | COG5537 | Cohesin | Whole_complex |
| [R] | COG5564 | Predicted TIM-barrel enzyme, possibly a dioxygenase | Whole_complex |
| [R] | COG5633 | Predicted periplasmic lipoprotein | Whole_complex |
| [P] | COG2375 | Siderophore-interacting protein | Whole_complex |
| [G] | COG2376 | Dihydroxyacetone kinase | Whole_complex |
| [C] | COG3262 | Ni,Fe-hydrogenase III component G | Whole_complex |
| [C] | COG3474 | Cytochrome c2 | Whole_complex |
| [T] | COG3830 | ACT domain-containing protein | Whole_complex |
| [R] | COG5198 | Protein tyrosine phosphatase-like protein (contains Pro instead of catalytic Arg) | Whole_complex |
| [Q] | COG5554 | Nitrogen fixation protein | Whole_complex |
| [M] | COG5632 | N-acetylmuramoyl-L-alanine amidase | Whole_complex |
| [C] | COG3038 | Cytochrome B561 | Whole_complex |
| [R] | COG5524 | Bacteriorhodopsin | Whole_complex |
| [L] | COG5142 | Oxidation resistance protein | Whole_complex |
| [R] | COG5645 | Predicted periplasmic lipoprotein | Whole_complex |
| [U] | COG5143 | Synaptobrevin/VAMP-like protein | Whole_complex |
| [A] | COG5183 | Protein involved in mRNA turnover and stability | Whole_complex |
| [Z] | COG5199 | Calponin | Whole_complex |
| [K] | COG5290 | IkappaB kinase complex, IKAP component | Whole_complex |
| [R] | COG5618 | Predicted periplasmic lipoprotein | Whole_complex |
| [N] | COG5651 | PPE-repeat proteins | Whole_complex |
| [U] | COG4537 | Competence protein ComGC | Whole_complex |
| [I] | COG4553 | Poly-beta-hydroxyalkanoate depolymerase | Whole_complex |
| [CH] | COG4635 | Flavodoxin | Whole_complex |
| [A] | COG5186 | Poly(A) polymerase | Whole_complex |
| [C] | COG2863 | Cytochrome c553 | Whole_complex |
| [D] | COG0206 | Cell division GTPase | Cycle |
| [D] | COG0445 | NAD/FAD-utilizing enzyme apparently involved in cell division | Cycle |
| [D] | COG0849 | Actin-like ATPase involved in cell division" | Cycle |
| [NT] | COG1776 | Chemotaxis protein CheC, inhibitor of MCP methylation" | Inhibition |
| [J] | COG0251 | Putative translation initiation inhibitor, yjgF family | Inhibitor |
| [O] | COG0760 | Parvulin-like peptidyl-prolyl isomerase | Metabolism |
| [K] | COG4271 | Predicted nucleotide-binding protein containing TIR domain" | Subunit |
| [NU] | COG0630 | Type IV secretory pathway, VirB11 components, and related ATPases involved in archaeal flagella biosynthesis | Pathway |
| [TK] | COG0745 | Response regulators consisting of a CheY-like receiver domain and a winged-helix DNA-binding domain Receiver" | Receiver |
| [E] | COG0347 | Nitrogen regulatory protein PII | Regulation |
| [K] | COG1420 | Transcriptional regulator of heat shock gene | Regulation |
| [K] | COG1678 | Putative transcriptional regulator | Regulation |
| [U] | COG0541 | Signal recognition particle GTPase | Sensing |
| [U] | COG0552 | Signal recognition particle GTPase | Sensing |
| [V] | COG0732 | Restriction endonuclease S subunits | Subunit |
| [F] | COG0104 | Adenylosuccinate synthase | Synthesis |
| [F] | COG0125 | Thymidylate kinase | Synthesis |
| [I] | COG1211 | 4-diphosphocytidyl-2-methyl-D-erithritol synthase | Synthesis |
| [M] | COG1212 | CMP-2-keto-3-deoxyoctulosonic acid synthetase | Synthesis |
| [F] | COG0518 | GMP synthase - Glutamine amidotransferase domain | Transport_container |
| [GER] | COG0697 | Permeases of the drug/metabolite transporter (DMT) superfamily" | Transport_container |
| [E] | COG0747 | ABC-type dipeptide transport system, periplasmic component | Transport_container |
| [N] | COG1291 | Flagellar motor component | Transportroute |
| [N] | COG1344 | Flagellin and related hook-associated proteins | Transportroute |
| [N] | COG1345 | Flagellar capping protein | Transportroute |
| [N] | COG1360 | Flagellar motor protein | Transportroute |
| [NU] | COG1377 | Flagellar biosynthesis pathway, component FlhB" | Transportroute |
| [N] | COG1419 | Flagellar GTP-binding protein" | Transportroute |
| [N] | COG1536 | Flagellar motor switch protein" | Transportroute |
| [N] | COG1558 | Flagellar basal body rod protein | Transportroute |
| [V] | COG1566 | Multidrug resistance efflux pump | Transportroute |
| [N] | COG1580 | Flagellar basal body-associated protein | Transportroute |
| [CP] | COG1668 | ABC-type Na+ efflux pump, permease component" | Transportroute |
| [NU] | COG1677 | Flagellar hook-basal body protein" | Transportroute |
| [N] | COG1706 | Flagellar basal-body P-ring protein" | Transportroute |
| [N] | COG1749 | Flagellar hook protein FlgE | Transportroute |
| [NU] | COG1766 | Flagellar biosynthesis/type III secretory pathway lipoprotein | Transportroute |
| [N] | COG1815 | Flagellar basal body protein | Transportroute |
| [EH] | COG0028 | Thiamine pyrophosphate-requiring enzymes [acetolactate synthase, pyruvate dehydrogenase (cytochrome), glyoxylate carboligase, phosphonopyruvate decarboxylase]" | Synthesis |
| [D] | COG0037 | Predicted ATPase of the PP-loop superfamily implicated in cell cycle control" | Regulation |
| [C] | COG0374 | Ni,Fe-hydrogenase I large subunit | Subunit |
| [G] | COG0395 | ABC-type sugar transport system, permease component" | Transport_container |
| [O] | COG0396 | ABC-type transport system involved in Fe-S cluster assembly, ATPase component" | Transport_container |
| [L] | COG0593 | ATPase involved in DNA replication initiation | Activation |
| [V] | COG0610 | Type I site-specific restriction-modification system, R (restriction) subunit and related helicases" | Subunit |
| [P] | COG0619 | ABC-type cobalt transport system, permease component CbiQ and related transporters" | Transport_container |
| [U] | COG0690 | Preprotein translocase subunit SecE | Subunit |
| [OU] | COG0740 | Protease subunit of ATP-dependent Clp proteases | Subunit |
| [L] | COG0749 | DNA polymerase I - 3'-5' exonuclease and polymerase domains | Subunit |
| [M] | COG0763 | Lipid A disaccharide synthetase | Synthesis |
| [I] | COG0777 | Acetyl-CoA carboxylase beta subunit | Subunit |
| [T] | COG0784 | FOG: CheY-like receiver | Receiver |
| [M] | COG0794 | Predicted sugar phosphate isomerase involved in capsule formation | Metabolism |
| [E] | COG0804 | Urea amidohydrolase (urease) alpha subunit | Subunit |
| [J] | COG0809 | S-adenosylmethionine:tRNA-ribosyltransferase-isomerase (queuine synthetase) | Synthesis |
| [M] | COG0810 | Periplasmic protein TonB, links inner and outer membranes | Barrier |
| [L] | COG0817 | Holliday junction resolvasome, endonuclease subunit | Subunit |
| [V] | COG1002 | Type II restriction enzyme, methylase subunits | Regulation |
| [C] | COG1034 | NADH dehydrogenase/NADH:ubiquinone oxidoreductase 75 kD subunit (chain G) | Subunit |
| [C] | COG1035 | Coenzyme F420-reducing hydrogenase, beta subunit | Subunit |
| [O] | COG1047 | FKBP-type peptidyl-prolyl cis-trans isomerases 2 | Metabolism |
| [C] | COG1053 | Succinate dehydrogenase/fumarate reductase, flavoprotein subunit | Subunit |
| [K] | COG1095 | DNA-directed RNA polymerase, subunit E | Subunit |
| [P] | COG1108 | ABC-type Mn2+/Zn2+ transport systems, permease components | Transport_container |
| [G] | COG1109 | Phosphomannomutase | Metabolism |
| [L] | COG1112 | Superfamily I DNA and RNA helicases and helicase subunits | Subunit |
| [C] | COG1140 | Nitrate reductase beta subunit | Subunit |
| [C] | COG1143 | Formate hydrogenlyase subunit 6/NADH:ubiquinone oxidoreductase 23 kD subunit (chain I) | Subunit |
| [C] | COG1144 | Pyruvate:ferredoxin oxidoreductase and related 2-oxoacid:ferredoxin oxidoreductases, delta subunit | Subunit |
| [C] | COG1148 | Heterodisulfide reductase, subunit A and related polyferredoxins | Subunit |
| [C] | COG1150 | Heterodisulfide reductase, subunit C | Subunit |
| [C] | COG1152 | CO dehydrogenase/acetyl-CoA synthase alpha subunit | Subunit |
| [C] | COG1156 | Archaeal/vacuolar-type H+-ATPase subunit B | Subunit |
| [NU] | COG1157 | Flagellar biosynthesis/type III secretory pathway ATPase | Transportroute |
| [K] | COG1191 | DNA-directed RNA polymerase specialized sigma subunit | Subunit |
| [C] | COG1229 | Formylmethanofuran dehydrogenase subunit A | Subunit |
| [H] | COG1239 | Mg-chelatase subunit ChlI | Subunit |
| [H] | COG1240 | Mg-chelatase subunit ChlD | Subunit |
| [C] | COG1252 | NADH dehydrogenase, FAD-containing subunit | Subunit |
| [J] | COG1258 | Predicted pseudouridylate synthase | Synthesis |
| [C] | COG1269 | Archaeal/vacuolar-type H+-ATPase subunit I | Subunit |
| [C] | COG1282 | NAD/NADP transhydrogenase beta subunit | Subunit |
| [P] | COG1283 | Na+/phosphate symporter | Transport_container |
| [C] | COG1290 | Cytochrome b subunit of the bc complex | Subunit |
| [C] | COG1294 | Cytochrome bd-type quinol oxidase, subunit 2 | Subunit |
| [E] | COG1305 | Transglutaminase-like enzymes, putative cysteine proteases | Degradation |
| [U] | COG1314 | Preprotein translocase subunit SecG | Subunit |
| [C] | COG1319 | Aerobic-type carbon monoxide dehydrogenase, middle subunit CoxM/CutM homologs | Subunit |
| [J] | COG1325 | Predicted exosome subunit | Subunit |
| [L] | COG1330 | Exonuclease V gamma subunit | Subunit |
| [P] | COG1348 | Nitrogenase subunit NifH (ATPase) | Subunit |
| [T] | COG1366 | Anti-anti-sigma regulatory factor (antagonist of anti-sigma factor) | Adjuvant |
| [J] | COG1369 | RNase P/RNase MRP subunit POP5 | Subunit |
| [L] | COG1381 | Recombinational DNA repair protein (RecF pathway) | Pathway |
| [C] | COG1436 | Archaeal/vacuolar-type H+-ATPase subunit F | Subunit |
| [K] | COG1438 | Arginine repressor | Inhibition |
| [NU] | COG1450 | Type II secretory pathway, component PulD | Pathway |
| [NU] | COG1459 | Type II secretory pathway, component PulF" | Pathway |
| [S] | COG1460 | DNA-directed RNA polymerase, subunit F | Subunit |
| [L] | COG1466 | DNA polymerase III, delta subunit | Subunit |
| [L] | COG1467 | Eukaryotic-type DNA primase, catalytic (small) subunit | Subunit |
| [J] | COG1500 | Predicted exosome subunit | Subunit |
| [G] | COG1523 | Type II secretory pathway, pullulanase PulA and related glycosidases | Pathway |
| [J] | COG1588 | RNase P/RNase MRP subunit p29 | Subunit |
| [L] | COG1599 | Single-stranded DNA-binding replication protein A (RPA), large (70 kD) subunit and related ssDNA-binding proteins" | Subunit |
| [J] | COG1603 | RNase P/RNase MRP subunit p30 | Subunit |
| [C] | COG1622 | Heme/copper-type cytochrome/quinol oxidases, subunit 2 | Subunit |
| [K] | COG1654 | Biotin operon repressor | Inhibitor |
| [I] | COG1657 | Squalene cyclase | Cycle |
| [N] | COG1681 | Archaeal flagellins | Transportroute |
| [T] | COG1734 | DnaK suppressor protein | Inhibition |
| [C] | COG1740 | Ni,Fe-hydrogenase I small subunit | Subunit |
| [K] | COG1761 | DNA-directed RNA polymerase, subunit L | Subunit |
| [E] | COG1775 | Benzoyl-CoA reductase/2-hydroxyglutaryl-CoA dehydratase subunit, BcrC/BadD/HgdB | Subunit |
| [U] | COG1826 | Sec-independent protein secretion pathway components" | Pathway |
| [C] | COG1845 | Heme/copper-type cytochrome/quinol oxidase, subunit 3 | Subunit |
| [U] | COG1862 | Preprotein translocase subunit YajC | Subunit |
| [N] | COG1868 | Flagellar motor switch protein | Transportroute |
| [NU] | COG1886 | Flagellar motor switch/type III secretory pathway protein | Transportroute |
| [H] | COG1903 | Cobalamin biosynthesis protein CbiD | Synthsis |
| [C] | COG1908 | Coenzyme F420-reducing hydrogenase, delta subunit | Subunit |
| [I] | COG1947 | 4-diphosphocytidyl-2C-methyl-D-erythritol 2-phosphate synthase | Synthesis |
| [NU] | COG1955 | Archaeal flagella assembly protein J" | Transportroute |
| [KT] | COG1974 | SOS-response transcriptional repressors (RecA-mediated autopeptidases) | Inhibition |
| [H] | COG1985 | Pyrimidine reductase, riboflavin biosynthesis | Synthesis |
| [NU] | COG1987 | Flagellar biosynthesis pathway, component FliQ | Pathway |
| [R] | COG1988 | Predicted membrane-bound metal-dependent hydrolases | Pathway |
| [NOU] | COG1989 | Type II secretory pathway, prepilin signal peptidase PulO and related peptidases | Pathway |
| [H] | COG1995 | Pyridoxal phosphate biosynthesis protein | Synthesis |
| [P] | COG2032 | Cu/Zn superoxide dismutase" | Matabolism |
| [N] | COG2063 | Flagellar basal body L-ring protein | Transportroute |
| [NU] | COG2064 | Flp pilus assembly protein TadC | Transportroute |
| [C] | COG2069 | CO dehydrogenase/acetyl-CoA synthase delta subunit (corrinoid Fe-S protein)" | Synthesis |
| [H] | COG2091 | Phosphopantetheinyl transferase" | Transport_container |
| [U] | COG2095 | Multiple antibiotic transporter" | Transport_container |
| [R] | COG2166 | SufE protein probably involved in Fe-S center assembly | Whole_complex |
| [Q] | COG2175 | Probable taurine catabolism dioxygenase | Catabolism |
| [D] | COG2184 | Protein involved in cell division | Cycle |
| [NT] | COG2201 | Chemotaxis response regulator containing a CheY-like receiver domain and a methylesterase domain" | Receiver |
| [T] | COG2204 | Response regulator containing CheY-like receiver, AAA-type ATPase, and DNA-binding domains" | Receiver |
| [J] | COG2269 | Truncated, possibly inactive, lysyl-tRNA synthetase (class II)" | Synthesis |
| [U] | COG2443 | Preprotein translocase subunit Sss1 | Subunit |
| [E] | COG2502 | Asparagine synthetase A | Synthesis |
| [NU] | COG2804 | Type II secretory pathway, ATPase PulE/Tfp pilus assembly pathway, ATPase PilB | Pathway |
| [M] | COG2829 | Outer membrane phospholipase A | Barrier |
| [U] | COG2831 | Hemolysin activation/secretion protein | Active |
| [NU] | COG2874 | Predicted ATPases involved in biogenesis of archaeal flagella | Transportroute |
| [H] | COG2918 | Gamma-glutamylcysteine synthetase x | Synthesis |
| [U] | COG2948 | Type IV secretory pathway, VirB10 components | Pathway |
| [R] | COG2961 | Protein involved in catabolism of external DNA | Catabolism |
| [L] | COG2974 | DNA recombination-dependent growth factor C | Adjuvant |
| [R] | COG3019 | Predicted metal-binding protein | Linear |
| [U] | COG3031 | Type II secretory pathway, component PulC | Pathway |
| [C] | COG3053 | Citrate lyase synthetase | Synthesis |
| [C] | COG3080 | Fumarate reductase subunit D | Subunit |
| [T] | COG3086 | Positive regulator of sigma E activity | Activation |
| [N] | COG3144 | Flagellar hook-length control protein | Transportroute |
| [U] | COG3149 | Type II secretory pathway, component PulM | Pathway |
| [U] | COG3156 | Type II secretory pathway, component PulK | Pathway |
| [NU] | COG3166 | Tfp pilus assembly protein PilN | Transportroute |
| [NU] | COG3167 | Tfp pilus assembly protein PilO" | Transportroute |
| [NU] | COG3168 | Tfp pilus assembly protein PilP" | Transportroute |
| [NU] | COG3170 | Tfp pilus assembly protein FimV" | Transportroute |
| [N] | COG3190 | Flagellar biogenesis protein" | Transportroute |
| [NU] | COG3215 | Tfp pilus assembly protein PilZ" | Transportroute |
| [U] | COG3267 | Type II secretory pathway, component ExeA (predicted ATPase) | Pathway |
| [KT] | COG3279 | Response regulator of the LytR/AlgR family | Receiver |
| [L] | COG3293 | Transposase and inactivated derivatives | Information_storage |
| [U] | COG3297 | Type II secretory pathway, component PulL | Pathway |
| [R] | COG3324 | Predicted enzyme related to lactoylglutathione lyase | Degradation |
| [K] | COG3327 | Phenylacetic acid-responsive transcriptional repressor" | Inhibition |
| [L] | COG3328 | Transposase and inactivated derivatives | Inhibition |
| [N] | COG3351 | Putative archaeal flagellar protein D/E | Transportroute |
| [N] | COG3352 | Putative archaeal flagellar protein C" | Transportroute |
| [N] | COG3353 | Putative archaeal flagellar protein F" | Transportroute |
| [N] | COG3354 | Putative archaeal flagellar protein G" | Transportroute |
| [NU] | COG3419 | Tfp pilus assembly protein, tip-associated adhesin PilY1 | Transportroute |
| [KT] | COG3437 | Response regulator containing a CheY-like receiver domain and an HD-GYP domain | Receiver |
| [U] | COG3451 | Type IV secretory pathway, VirB4 components | Pathway |
| [MU] | COG3468 | Type V secretory pathway, adhesin AidA | Pathway |
| [O] | COG3484 | Predicted proteasome-type protease | Degradation |
| [U] | COG3504 | Type IV secretory pathway, VirB9 components | Pathway |
| [U] | COG3505 | Type IV secretory pathway, VirD4 components" | Pathway |
| [NU] | COG3539 | P pilus assembly protein, pilin FimA | Transportroute |
| [T] | COG3642 | Mn2+-dependent serine/threonine protein kinase | Sensing |
| [U] | COG3701 | Type IV secretory pathway, TrbF components | Pathway |
| [U] | COG3702 | Type IV secretory pathway, VirB3 components" | Pathway |
| [U] | COG3704 | Type IV secretory pathway, VirB6 components" | Pathway |
| [T] | COG3706 | Response regulator containing a CheY-like receiver domain and a GGDEF domain" | Receiver |
| [T] | COG3707 | Response regulator with putative antiterminator output domain" | Receiver |
| [U] | COG3736 | Type IV secretory pathway, component VirB8" | Pathway |
| [U] | COG3745 | Flp pilus assembly protein CpaB | Transportroute |
| [U] | COG3838 | Type IV secretory pathway, VirB2 components (pilins) | Pathway |
| [U] | COG3843 | Type IV secretory pathway, VirD2 components (relaxase)" | Pathway |
| [U] | COG3846 | Type IV secretory pathway, TrbL components" | Pathway |
| [U] | COG3847 | Flp pilus assembly protein, pilin Flp | Transportroute |
| [U] | COG3946 | Type IV secretory pathway, VirJ component | Pathway |
| [J] | COG4108 | Peptide chain release factor RF-3 | Adjuvant |
| [D] | COG4118 | Antitoxin of toxin-antitoxin stability system | Network |
| [G] | COG4154 | Fucose dissimilation pathway protein FucU | Pathway |
| [R] | COG4416 | Mu-like prophage protein Com | Information_Storage |
| [G] | COG4421 | Capsular polysaccharide biosynthesis protein" | Information_Storage |
| [C] | COG4451 | Ribulose bisphosphate carboxylase small subunit | Subunit |
| [R] | COG4455 | Protein of avirulence locus involved in temperature-dependent protein secretion" | Information_Storage |
| [S] | COG4456 | Virulence-associated protein and related proteins" | Information_Storage |
| [C] | COG4459 | Periplasmic nitrate reductase system, NapE component" | Network |
| [D] | COG4477 | Negative regulator of septation ring formation" | Inhibition |
| [KT] | COG4565 | Response regulator of citrate/malate metabolism | Receiver |
| [TK] | COG4567 | Response regulator consisting of a CheY-like receiver domain and a Fis-type HTH domain" | Receiver |
| [R] | COG4572 | Putative cation transport regulator" | Regulation |
| [QC] | COG4576 | Carbon dioxide concentrating mechanism/carboxysome shell protein" | Network |
| [QC] | COG4577 | Carbon dioxide concentrating mechanism/carboxysome shell protein" | Network |
| [U] | COG4669 | Type III secretory pathway, lipoprotein EscJ | Pathway |
| [T] | COG4753 | Response regulator containing CheY-like receiver domain and AraC-type DNA-binding domain" | Receiver |
| [P] | COG4771 | Outer membrane receptor for ferrienterochelin and colicins" | Receiver |
| [P] | COG4772 | Outer membrane receptor for Fe3+-dicitrate" | Receiver |
| [P] | COG4773 | Outer membrane receptor for ferric coprogen and ferric-rhodotorulic acid" | Receiver |
| [P] | COG4774 | Outer membrane receptor for monomeric catechols" | Receiver |
| [N] | COG4786 | Flagellar basal body rod protein" | Transportroute |
| [N] | COG4787 | Flagellar basal body rod protein" | Transportroute |
| [U] | COG4789 | Type III secretory pathway, component EscV" | Pathway |
| [U] | COG4790 | Type III secretory pathway, component EscR" | Pathway |
| [U] | COG4791 | Type III secretory pathway, component EscT" | Pathway |
| [U] | COG4792 | Type III secretory pathway, component EscU" | Pathway |
| [U] | COG4794 | Type III secretory pathway, component EscS" | Pathway |
| [U] | COG4795 | Type II secretory pathway, component PulJ" | Pathway |
| [D] | COG4839 | Protein required for the initiation of cell division | Active |
| [OU] | COG4959 | Type IV secretory pathway, protease TraF | Pathway |
| [C] | COG5013 | Nitrate reductase alpha subunit | Subunit |
| [R] | COG5018 | Inhibitor of the KinA pathway to sporulation, predicted exonuclease | Inhibition |
| [L] | COG5055 | Recombination DNA repair protein (RAD52 pathway) | Pathway |
| [U] | COG5101 | Nuclear transport receptor CRM1/MSN5 (importin beta superfamily) | Receiver |
| [UT] | COG5130 | Prenylated rab acceptor 1 and related proteins | Receiver |
| [NU] | COG5268 | Type IV secretory pathway, TrbD component | Pathway |
